# Synthesis, Biological Evaluation, and In Silico Characterization of Novel Imidazothiadiazole–Chalcone Hybrids as Multi-Target Enzyme Inhibitors

**DOI:** 10.3390/ph18070962

**Published:** 2025-06-26

**Authors:** Hakan Alici, Senol Topuz, Kadir Demir, Parham Taslimi, Hakan Tahtaci

**Affiliations:** 1 Department of Physics, Faculty of Science, Zonguldak Bülent Ecevit University, 67100 Zonguldak, Türkiye; senol.topuz@fbe.karaelmas.edu.tr (S.T.); kadirdemir@beun.edu.tr (K.D.); 2 Department of Biotechnology, Faculty of Science, Bartin University, 74110 Bartın, Türkiye; ptaslimi@bartin.edu.tr; 3 Department of Chemistry, Faculty of Science, Karabuk University, 78050 Karabuk, Türkiye

**Keywords:** imidazothiadiazole, chalcone hybrids, carbonic anhydrase inhibitors, acetylcholinesterase inhibitors, butyrylcholinesterase inhibitors, molecular docking, molecular dynamics simulation

## Abstract

**Background/Objectives:** The need for dual-targeted enzyme inhibitors is critical in addressing complex diseases like Alzheimer’s and glaucoma. Imidazothiadiazole and chalcone moieties are known for diverse bioactivities. This study aimed to develop novel imidazothiadiazole–chalcone hybrids as potential inhibitors of acetylcholinesterase (AChE), butyrylcholinesterase (BChE), and human carbonic anhydrase isoforms (hCAs), specifically hCA I and hCA II. **Methods:** Four hybrid molecules (**8a–8d**) were synthesized and structurally confirmed via ^1^H NMR, ^13^C NMR, FT-IR, MS, and elemental analysis techniques. Their enzyme inhibitory activities were assessed using Ellman’s and Verpoorte’s methods. Molecular docking and 100 ns molecular dynamics (MD) simulations were conducted to examine binding interactions. Absorption, distribution, metabolism, excretion, and toxicity (ADMET) properties were predicted using the pkCSM platform. **Results:** All compounds showed strong enzyme inhibition: AChE (K_i_: 3.86–11.35 nM), BChE (K_i_: 1.01–1.78 nM), hCA I (K_i_: 45.13–81.24 nM), and hCA II (K_i_: 36.08–52.45 nM). Docking analyses confirmed favorable binding, particularly with active-site residues. MD simulations demonstrated stable interactions throughout 100 ns. Compound **8a** exhibited the highest cholinesterase inhibition, while compounds **8d** and **8c** were the most potent against hCA I and hCA II, respectively. The ADMET results showed high absorption and acceptable safety, with mild mutagenicity or cardiotoxicity concerns in select compounds. **Conclusions:** These findings suggest that imidazothiadiazole–chalcone hybrids are promising multi-target enzyme inhibitors. Their potent activity, structural stability, and pharmacokinetic potential support their further development for therapeutic use in neurodegenerative and ocular diseases.

## 1. Introduction

Heterocyclic compounds play a fundamental role in drug discovery due to their structural diversity and broad range of biological activities. Heterocyclic compounds are molecules with ring structures consisting of at least one atom besides carbon, such as nitrogen, oxygen, or sulfur [1]. Among the heterocyclic compounds, imidazole and 1,3,4-thiadiazole structures are among the most widely studied compounds in the field of pharmaceutical chemistry for the discovery of new drugs due to their versatile uses and especially their various biological activities [2,3,4]. Hybrid molecules in which a bridging nitrogen atom links these two heterocyclic aromatic compounds are called imidazothiadiazoles [5,6]. Imidazothiadiazoles, fused structures containing both nitrogen and sulfur, have an important place in medicinal chemistry, as they exhibit a broad spectrum of biological activities, such as antibacterial, antifungal, antiviral, anticonvulsant, antidepressant, anti-inflammatory, antihypertensive, anticancer, and enzyme inhibition activities [7,8,9,10,11,12,13,14,15,16,17]. Given their rich pharmacological potential, imidazothiadiazoles are promising scaffolds for hybridization with other bioactive moieties, such as chalcones, to enhance therapeutic efficacy and target diversity. Currently, chalcones stand out due to their flexibility and diverse therapeutic applications. These organic compounds have an *α*,*β*-unsaturated carbonyl group linking two aromatic rings and play an important role in flavonoid biosynthesis [18,19]. Their flexible structures contribute significantly to drug development [20]. These compounds show various biological activities, including antimicrobial, anti-inflammatory, anticancer, anti-Alzheimer’s, antidiabetic, enzyme inhibitory, and antioxidant effects [10,11,12,14,21]. In addition, some studies have reported that chalcone derivatives also have pancreatic lipase enzyme inhibition potential [22,23]. These findings are promising, but more research is needed to comprehensively evaluate their efficacy and potential for clinical application.

To the best of our knowledge, this is the first report that describes the synthesis of specific imidazothiadiazole–chalcone hybrid compounds and evaluates their activity against hCA I, hCA II, AChE, and BChE. Furthermore, despite extensive studies on these scaffolds individually, no prior research has systematically explored imidazothiadiazole–chalcone hybrids as dual inhibitors of cholinesterases and CAs. These complementary pharmacophores provide a rationale for designing novel hybrid inhibitors capable of targeting multiple disease-related enzymes.

To assess the potential of these hybrids as multi-target therapeutics, we investigated their potential against key metabolic enzymes implicated in neurodegenerative and ophthalmic disorders. In this respect, CAs are metalloenzymes that catalyze the reversible hydration of CO_2_ to bicarbonate (HCO_3_^−^) and a proton (H^+^) and contribute to various physiological functions. There are 15 human α-CA isoforms, of which 12 have enzymatic activity [24]. In particular, sulfonamide-based CA inhibitors have long been used to treat diseases such as glaucoma, epilepsy, and peptic ulcer [25]. Significant efforts have been made to develop isoform-specific inhibitors to expand their therapeutic scope in conditions such as neuropathic pain, arthritis, and cancer [26]. The physiological and clinical significance of hCA I and hCA II, which are among these 15 isoforms, has been thoroughly investigated. These functions include aiding in respiration, preserving the acid–base balance in the kidneys, and promoting bone metabolism [27,28]. Furthermore, a few serine hydrolase enzymes, including BChE and AChE, are crucial for the nervous system [29]. Neurotransmission requires AChE, while BChE’s physiological role is unclear but it may co-regulate cholinergic transmission [30]. Both work similarly, although AChE hydrolyzes acetylcholine more selectively and efficiently [31]. These cholinesterase enzymes are implicated in various pathological conditions, including Alzheimer’s disease [32]. AChE and BChE can serve as biochemical markers for various pathologies, including nerve gases, organophosphorus pesticides, and the overdose of drugs used in neurodegenerative disorders [33].

Based on the information obtained from the existing literature, this research aimed to develop new hybrid molecules combining imidazothiadiazole and chalcone frameworks. The structures of the synthesized compounds were confirmed using a range of analytical techniques, such as ^1^H NMR, ^13^C NMR, FT-IR, mass spectrometry, and elemental analyses. The potential of these compounds to inhibit hCA I, hCA II, AChE, and BChE was evaluated by both in vitro experiments and computational methods, including molecular docking and molecular dynamics simulations.

Despite their prominence, these compounds have not been previously investigated in combination in this therapeutic context. This study introduces previously unexplored hybrid scaffolds and contributes to the development of novel multi-target enzyme inhibitors in pharmaceutical chemistry.

## 2. Results and Discussion

### 2.1. Chemistry

**Scheme 1:**
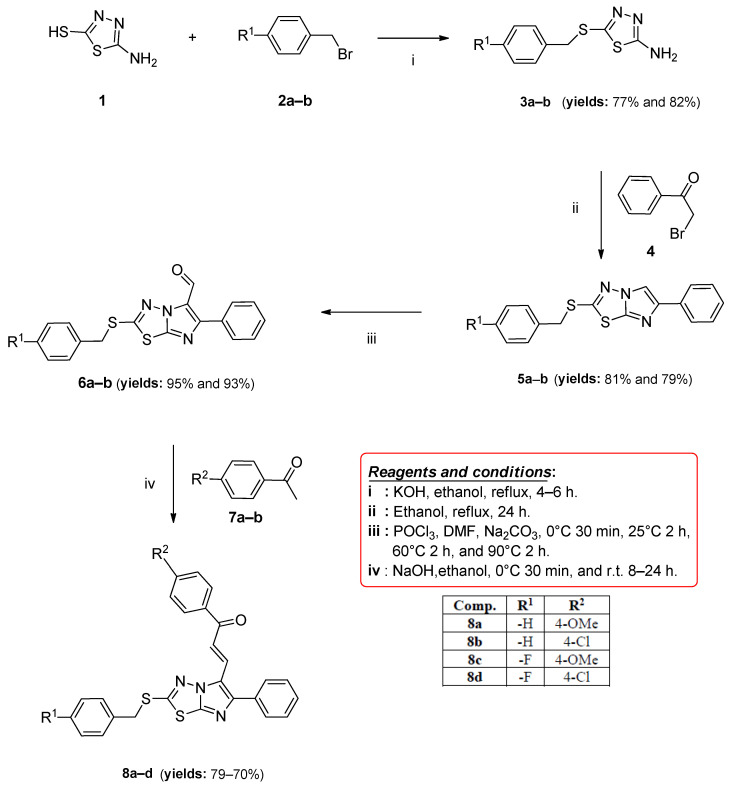
Synthetic pathways for the synthesis of chalcone-derived compounds containing the imidazothiadiazole nucleus (**8a–d**).

In this study, four novel chalcone derivatives (**8a–d**) containing the 2,5,6-trisubstituted imidazothiadiazole core were synthesized using Vilsmeier–Haack and Claisen–Schmidt reactions through the synthetic pathways shown in Scheme 1.

In the first step of the synthesis, the starting compounds, 2-amino-1,3,4-thiadiazole derivatives (**3a–b**), were synthesized. These compounds were obtained following the method reported in the literature through the reaction of 5-amino-1,3,4-thiadiazole-2-thiol (**1**) with benzyl bromide (**2a**) and 4-fluorobenzyl bromide (**2b**) in the presence of KOH, yielding 77% and 82%, respectively, in accordance with the method described in the literature [34,35,36,37,38].

In the second step, 2,6-disubstituted imidazo[2,1-*b*][1,3,4]thiadiazole compounds (**5a–b**), which contain both imidazole and 1,3,4-thiadiazole rings, were obtained. These compounds were synthesized from the starting compounds (**3a–b**) through a cyclization reaction with 2-bromoacetophenone (**4**) in absolute ethanol, yielding 81% and 79%, respectively, following the method described in the literature [34,35,36,37,38].

In the third step, an aldehyde group was introduced at the 5-position of the 2,6-disubstituted imidazo[2,1-*b*][1,3,4]thiadiazole compounds (**5a–b**) using the Vilsmeier–Haack reaction, yielding 2-(benzylthio)-6-phenylimidazo[2,1-*b*][1,3,4]thiadiazol-5-carbaldehyde derivatives (**6a,b**). For this purpose, a fresh Vilsmeier–Haack reagent was first prepared in situ using phosphoryl chloride (POCl_3_) and DMF. Then, this reagent was reacted with the 2,6-disubstituted imidazo[2,1-*b*][1,3,4]thiadiazole compounds (**5a–b**), resulting in high yields (95% and 93%, respectively), following the method described in the literature [35].

The presence of carbonyl stretching bands observed in the range of 1671–1640 cm^−1^ in the FT-IR spectra of these compounds provides strong evidence for the formation of 2-(benzylthio)-6-phenylimidazo[2,1-*b*][1,3,4]thiadiazol-5-carbaldehyde derivatives (**6a–b**).

In the ^1^H NMR spectra, the most significant confirmation of the formation of these compounds (**6a–b**) is the disappearance of the singlet signal corresponding to the C_5_-H proton, previously observed at 8.64 ppm in the starting compounds (**5a–b**), and the appearance of new singlet signals at 10.05 and 10.04 ppm, respectively, corresponding to the aldehyde proton. Similarly, the ^13^C NMR spectra show specific peaks for the carbonyl carbon (C=O) at 177.35 ppm, further supporting the formation of these compounds. These spectral data align well with the literature [4,35,39].

In the final step of the synthesis, the aldehyde derivatives (**6a–b**) obtained in step 3 were reacted with 4-methoxyacetophenone (**7a**) and 4-chloroacetophenone (**7b**) via Claisen–Schmidt reactions, yielding the target chalcone derivatives (**8a–d**) containing the imidazo[2,1-*b*][1,3,4]thiadiazole core. For this reaction, methyl ketones were first deprotonated using NaOH in ethanol under an ice bath to generate the enolate anion. The aldehyde derivatives (**6a–b**) obtained in step 3 were then slowly added to the reaction mixture, leading to the formation of the target compounds (**8a–d**) in good yields (79–70%).

In the FT-IR spectra of the target compounds, carbonyl stretching bands were observed in the range of 1649–1644 cm^−1^, similar to those of compounds **6a–b**. Additionally, an increase in the peak intensity was observed in the aromatic C-H region (3100–3000 cm^−1^), indicating the presence of newly introduced aromatic groups.

In the ^1^H NMR spectra, a key indicator confirming the formation of the target compounds is the disappearance of the singlet peaks corresponding to the aldehyde group at 10.05 and 10.04 ppm. Instead, a significant increase in the peak intensity is observed in the aromatic region (7.5–8.5 ppm) due to the presence of newly introduced aromatic groups. In the ^1^H NMR spectra of the target compounds (**8a–d**), the most important evidence that the chalcones were formed in the trans-alkene or *E*-form are the two prominent doublet peaks at 7.71–7.60 ppm and 7.35–7.26 ppm with a coupling constant (*J*) between 14.5 and 16.1 Hz. Compounds (**8a–d**), with a combined *J*-value of (14.5–16.1 Hz) for *α* and *β* hydrogen, revealed the *E*-configuration characteristics due to the large value of *J*, which undoubtedly discloses transgeometry for the chalcones. This finding aligns closely with the results of similar studies cited in the existing literature [40,41,42,43,44]. The chemical shifts, integration, and *J*-values of all the protons, influenced by the incorporation of substituted methyl ketones, further support the proposed structures.

In the ^13^C NMR spectra of the target compounds, the carbon peaks corresponding to the carbonyl group shifted slightly downfield compared to compounds **6a–b**, appearing in the range of 188.70–188.34 ppm. Similarly, the incorporation of aromatic groups led to a notable increase in the peak intensity in the aromatic region (140–110 ppm). These values are also consistent with the literature [4,39,40,45]. The number of carbon atoms in the compounds and the observed carbon peaks in the spectra fully confirm the proposed structures.

The structures of all the synthesized compounds were further verified by mass spectrometry. The base peaks and molecular peaks observed in the mass spectra were consistent with the expected values, confirming the structures through comprehensive spectroscopic analysis.

All spectroscopic data related to the synthesized compounds are provided in detail in the Experimental Synthesis Section, and the corresponding spectra are included in the Supplementary Materials Section (Figures S1–S40).

### 2.2. Biological Activity Studies

In this study, we describe how novel imidazothiadiazole derivatives (**8a–d**) decrease enzyme activity in vitro. In addition to the well-known CA inhibitor AZA, other CA inhibitors, like methazolamide, topiramate, and zonisamide, have also been approved for the treatment of epileptic disorders and epilepsy. These medications are still used in combination with other antiepileptic drugs, particularly in cases of drug-resistant epilepsy or when myoclonic, partial, and primary generalized tonic–clonic seizures are absent [46].

Here, to facilitate structural comparison and assist in the interpretation of the biological activity results, the R^1^ and R^2^ substituents of compounds **8a–8d** are summarized in Table 1.

**Figure 1:**
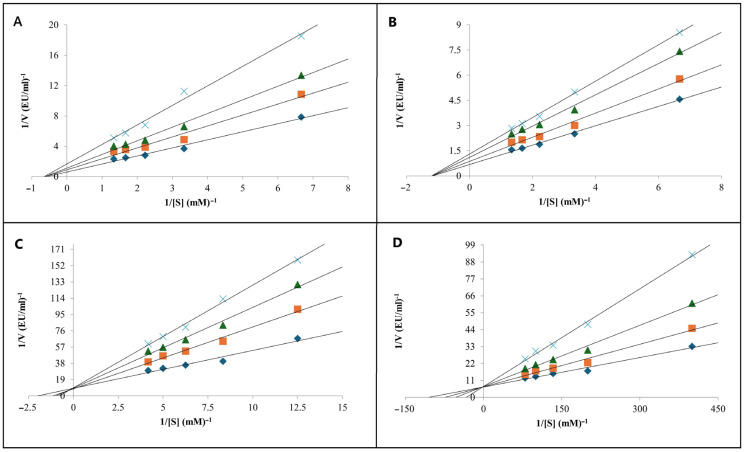
Lineweaver–Burk plots of best inhibitors for hCA I (**A**), hCA II (**B**), AChE (**C**), and BChE (**D**). The reciprocal plots (1/V vs. 1/[S]) were constructed using varying substrate concentrations in the absence and presence of different inhibitor concentrations to determine the type of inhibition and calculate the K_i_ values. In this study, the most effective enzyme inhibitors were compound **8a** for AChE and BChE, compound **8d** for hCA I, and compound **8c** for hCA II. The inhibition type was determined to be non-competitive for hCA I and hCA II, and competitive for AChE and BChE. In the figure: × I3 (the third inhibitor); 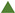 I2 (the second inhibitor); 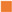 I1 (the first inhibitor); 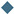 control (no inhibitor).

Also, Table 2 displays the activity results: The slow cytosolic isoform hCA I was suppressed by the investigated novel imidazothiadiazole derivatives (**8a–d**), with K_i_ values varying between 45.13 ± 3.91 and 81.24 ± 4.36 nM. Compound **8d** additionally demonstrated the greatest hCA I isoenzyme inhibitory properties, with a K_i_ value of 45.13 ± 3.91 nM. It is also widely known that all the new compounds have binding affinities for isoenzymes of hCA. AZA, a commonly used and conventional drug, has a K_i_ value of 145.73 ± 8.03 nM, as shown in Table 2 and Figure 1. Therefore, when compared to the clinically used CA inhibitor AZA, the compounds under investigation displayed superior inhibitory characteristics. The IC_50_ values of the compounds and the standards were as follows: compound **8d** (39.14 nM, r^2^: 0.961) < compound **8c** (42.16 nM, r^2^: 0.989) < compound **8b** (64.37 nM, r^2^: 0.936) < compound **8a** (73.05 nM, r^2^: 0.983) < AZA (129.03 μM, r^2^: 0.907) for hCA I. Dual-targeting inhibitors, which seek to concurrently modify two distinct targets or pathways within a disease context, hold promise as a workable therapeutic approach in drug discovery. Because it can increase drug efficacy by preventing the development of drug resistance, lowering the dosage of individual drugs, and limiting the risk of side effects, this approach is especially beneficial when redundant or compensatory pathways limit the effectiveness of single-target therapies [47]. This approach has been frequently used in the case of hCAs as well, which has resulted in the creation of compounds that can block hCA in addition to another target. Dual-targeting inhibitors of hCA enzymes have been the subject of numerous investigations in recent years, namely, for the treatment of complicated diseases like cancer, glaucoma, neurological disorders, and inflammatory ailments [48].

Altitude sickness, glaucoma, edema, epilepsy, and renal problems are all brought on by the disruption or hyperactivity of CA II. Nearly 67 million individuals worldwide suffer from glaucoma, the second most common cause of blindness and a quiet thief of sight. Inhibiting the action of CA II can diminish the release of bicarbonate ions in aqueous humor, lowering the intraocular pressure and delaying the course of glaucoma. There are about 25 CAIs that are marketed as medications, and the majority of them have the sulfonamide moiety as a necessary feature that interacts with the CAs’ active site [49]. Acetazolamide, diclofenamide, dorzolamide, methazolamide, ethoxzolamide, brinzolamide, celecoxib, valdecoxib, zonisamide, and sulthiame are some of the major commercially available CAIs that are taken via various methods. Indisulam and COU-MATE-667 are two CAIs that are currently undergoing clinical development. Regrettably, these medications have some negative side effects, including weariness, a metallic taste, numbness and tingling in the limbs, renal calculi, gastrointestinal discomfort, metabolic acidosis, and temporary myopia [50]. As a result, finding novel CAIs with fewer side effects is currently a major problem in the field of ophthalmic drug discovery. The novel compounds (**8a–d**) that were studied here also successfully inhibited hCA II. These medications’ K_i_ values, which varied from 36.08 ± 5.36 nM to 52.45 ± 6.24 nM, indicated that they significantly suppressed hCA II. These values are better than those of the commercially used drug AZA (a K_i_ of 112.63 ± 6.87 nM). With a K_i_ value of 36.08 ± 5.36 nM, compound **8c** demonstrated an excellent inhibitory profile against cytosolic hCA II (Table 2 and Figure 1). All of the investigated novel compounds (**8a–d**) showed significant inhibition against hCA II. The IC_50_ values of the compounds and the standards were as follows: compound **8c** (30.21 nM, r^2^: 0.983) < compound **8d** (34.08 nM, r^2^: 0.940) < compound **8a** (41.07 nM, r^2^: 0.947) < compound **8b** (49.32 nM, r^2^: 0.924) < AZA (102.55 nM, r^2^: 0.936) for hCA II. As a result, the compounds under investigation exhibited superior inhibitory characteristics in comparison to the currently used CA inhibitor AZA. In another study, Manasa et al. [51] conducted a similar study, in which a novel series of imidazo[2,1-*b*]thiazole-sulfonyl piperazine conjugates were synthesized and tested for their CA inhibitory potency against four isoforms: the cytosolic isozymes hCA I and II, and the transmembrane tumor-associated isoforms hCA IX and hCA XII, using acetazolamide as the standard drug. The findings indicated that while none of the compounds were active against hCA I, IX, or XII (K_i_ > 100 µM), the majority of them had selective activity against hCA II.

The synthesized compounds (**8a–d**) significantly suppressed AChE and BChE (Table 2 and Figure 1). The K_i_ values of these novel compounds ranged from 1.01 ± 0.08 to 1.78 ± 0.12 nM for BChE and from 3.86 ± 0.20 to 11.35 ± 1.02 nM for AChE. Furthermore, the conventional inhibitor TAC displayed K_i_ values of 3.57 ± 0.35 toward BChE and 14.27 ± 2.03 nM for AChE. Compound **8a** demonstrated an excellent inhibition profile against AChE and BChE, with K_i_ values of 3.86 ± 0.20 and 1.01 ± 0.08 nM, respectively. All of the compounds (**8a–d**) demonstrated strong inhibition against AChE and BChE enzymes. The IC_50_ values of the compounds and the standards were as follows: compound **8a** (4.36 nM, r^2^: 0.945) < compound **8b** (6.77 nM, r^2^: 0.929) < compound **8d** (9.30 nM, r^2^: 0.946) < compound **8c** (13.04 nM, r^2^: 0.993) < TAC (20.34 nM, r^2^: 0.968) for AChE, while for the BChE enzyme, they were as follows: compound **8** (1.37 nM, r^2^: 0.937) < compound **8b** (1.95 nM, r^2^: 0.902) < compound **8d** (1.98 nM, r^2^: 0.938) < compound **8c** (2.03 nM, r^2^: 0.915) < TAC (4.35 nM, r^2^: 0.905). Sarıkaya and his colleagues [52] carried out a similar investigation in which they synthesized novel thiazole–chalcone analogs and investigated their inhibitory effects on AChE. Every compound decreased the activity of AChE. Since our investigation involved novel imidazothiadiazole–chalcone hybrids and the compounds’ active groups, our results were at the nanomolar level, and when compared to Sarıkaya’s study, we obtained good results. To treat AD symptoms, cholinesterase (AChE and BChE) inhibitors that preserve the choline levels in the synaptic gap are crucial. AChE and BChE inhibitors have been used in several studies to combat AD. Only a small number of medications, including donepezil, galantamine, rivastigmine, and tacrine, are authorized as cholinesterase inhibitors. Cholinesterase inhibitors are therefore required to combat AD [53]. Some imidazo-thiadiazole-based chalcone compounds were created in 2022 by Abhinandan A. Alman et al. as possible EGFR inhibitors. Molecular docking studies were used to screen the proposed derivative, which was then synthesized and tested for its in vitro anticancer efficacy. The MTT assay was used to test the synthetic compounds’ in vitro cytotoxicity against MCF-7 (breast cancer) and A549 (lung cancer) cells. Every synthetic compound showed a good range of IC_50_ values between 4 and 59 µm/mL and produced cytotoxicity to MCF-7 and A549 [54]. Similarly, in 2022, a novel series of imidazo[2,1-*b*]thiazole-based chalcone derivatives were designed, synthesized, and tested for their anticancer activities by Ddaou et al. The cytotoxic abilities of these compounds were tested against four different types of cancer cell lines: colorectal adenocarcinoma (HT-29), lung carcinoma (A-549), breast adenocarcinoma (MCF-7), and mouse fibroblast (3T3-L1) cells using XTT assays. The XTT results revealed that all the test compounds exhibited much higher cytotoxic activity on cancer cells than on normal 3T3-L1 cells. Additionally, in silico molecular docking approaches were performed to confirm the experimental observations and investigate the efficacy of these compounds [55]. Therefore, after reviewing the literature, we found that the synthesized compounds exhibited significant activity, with meaningful IC_50_ values obtained for both enzymatic inhibition and anticancer effects. Supported by in vitro and in silico studies, these results suggest that our compounds may be suitable candidates for drug design and further in vivo investigations.

Although the compounds demonstrated strong enzyme inhibition and favorable pharmacokinetic profiles, further validation in relevant neuronal cell lines such as SH-SY5Y is essential to assess their functional activity, neuroprotective potential, and cellular safety. Accordingly, upcoming studies will include viability assays (e.g., MTT or LDH) to evaluate their cytotoxicity and establish the therapeutic concentration ranges.

### 2.3. Molecular Docking Studies

To evaluate the binding affinity of the synthesized compounds toward their enzymatic targets, molecular docking simulations were performed.

For an easier comparison, the molecular docking scores and IC_50_ values of the synthesized compounds (**8a–d**) against four key enzymes, AChE, BChE, hCA I, and hCA II, are listed in Table 3. As seen in the table, the results demonstrate consistent inhibitory activity in line with the predicted docking affinities, supporting the validity of the computational findings.

Although the AChE and BChE enzymes used in the in vitro assays were derived from non-human sources (Electrophorus electricus and Equus caballus, respectively), pairwise sequence alignment analyses confirmed high levels of sequence identity (76.9% for AChE and 90.1% for BChE) and the strong conservation of the catalytic triads and key binding-site residues with their human counterparts used in the docking studies. For hCA I and II, both experimental and computational studies were conducted using human isoenzymes, eliminating any source discrepancy. These findings, supported by the alignments provided in Supplementary Figures S41 and S42, affirm the structural and functional relevance of the in silico models to the experimental systems.

Molecular docking studies with the hCA I isoenzyme show a significant structural–biological correlation between the binding behavior of the four synthesized compounds and their in vitro inhibitory activity.

**Figure 2:**
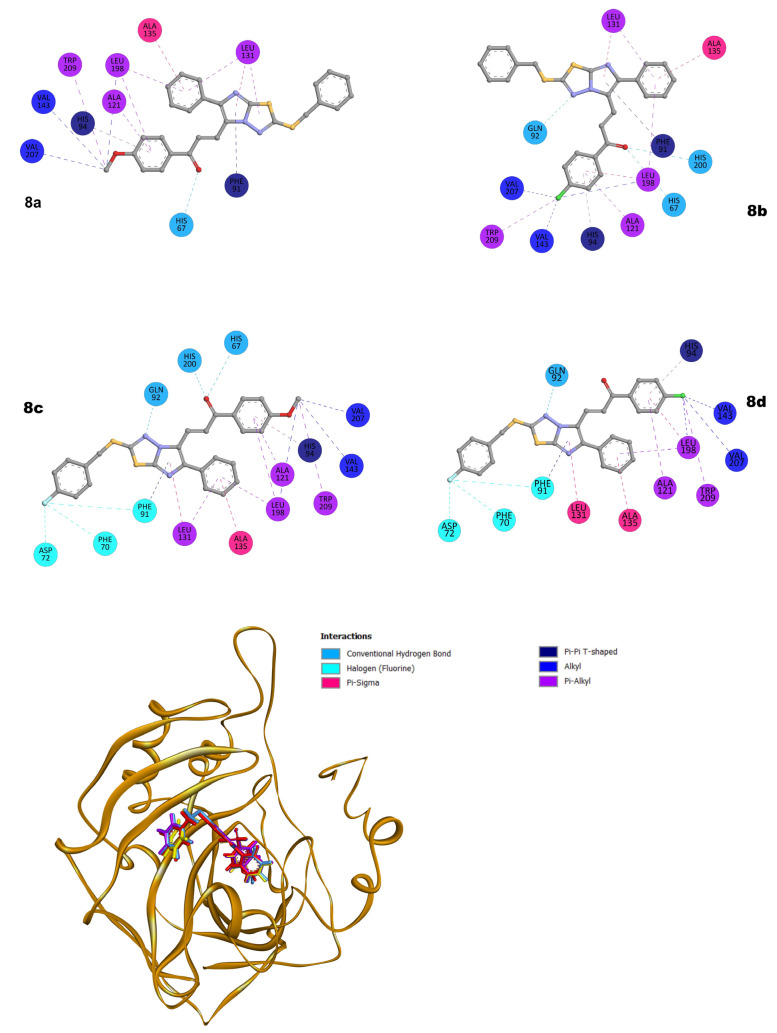
Molecular docking poses of compounds **8a–d** within the active site of hCA I.

Accordingly, as seen in Figure 2, compound **8a** showed hydrogen bonding with HIS67 and electrostatic π-cation interaction with HIS94. It also formed π-π T-shaped interactions with PHE91 and HIS94 and π-alkyl bonds with aromatic and hydrophobic residues such as TRP209 and LEU198. The binding energy is −10.1 kcal/mol, and this binding profile indicates a moderate affinity compared to the other compounds. Also, it can be said that this output is in agreement with the compound’s Kᵢ value of 81.24 nM determined for hCA I. For compound **8b**, it can be said that it increased its binding stability by forming three separate hydrogen bonds with HIS67, GLN92, and HIS200. In addition, π-cation interaction with HIS94 and π-π stacking with PHE91 support an important attachment at the binding site. Similarly, the binding diversity and orientation of this compound correlate with a Kᵢ value of 78.01 nM with an affinity of −10.6 kcal/mol. Compound **8c**, in addition to hydrogen bonds, formed triple halogen bonds with PHE70, ASP72, and PHE91, and it also performed π-cation interaction with HIS94. Also, thanks to the hydrophobic and stacking interactions with PHE91 and TRP209, it acquired an effective position in the binding site. This compound has the second most potent inhibitory profile, with a binding energy of −11.0 kcal/mol, in agreement with the Kᵢ value of 48.36 nM measured in vitro. For compound **8d**, it is observed that it shows multiple interactions with PHE70, ASP72, and PHE91 via hydrogen bonds with GLN92 and halogen groups. Furthermore, it can be stated that π-π stacking with HIS94 and additional π-alkyl interactions with PHE131 also strengthen its binding stability. In summary, this versatile binding pattern indicates a strong affinity of the molecule with hCA I and provides a direct agreement between the binding score of −11.1 kcal/mol and the Kᵢ value of 45.13 nM measured in vitro.

In conclusion, the docking analyses performed for the hCA I isoenzyme show a high structural overlap with the in vitro enzyme inhibition data. In particular, multiple binding interactions with catalytic-site residues such as HIS67, HIS94, and PHE91 play a decisive role in inhibitory activity. Accordingly, it can be inferred that compound **8d** stands out as the most potent hCA I inhibitor in terms of both the diversity of its binding profile and its in vitro activity.

**Figure 3:**
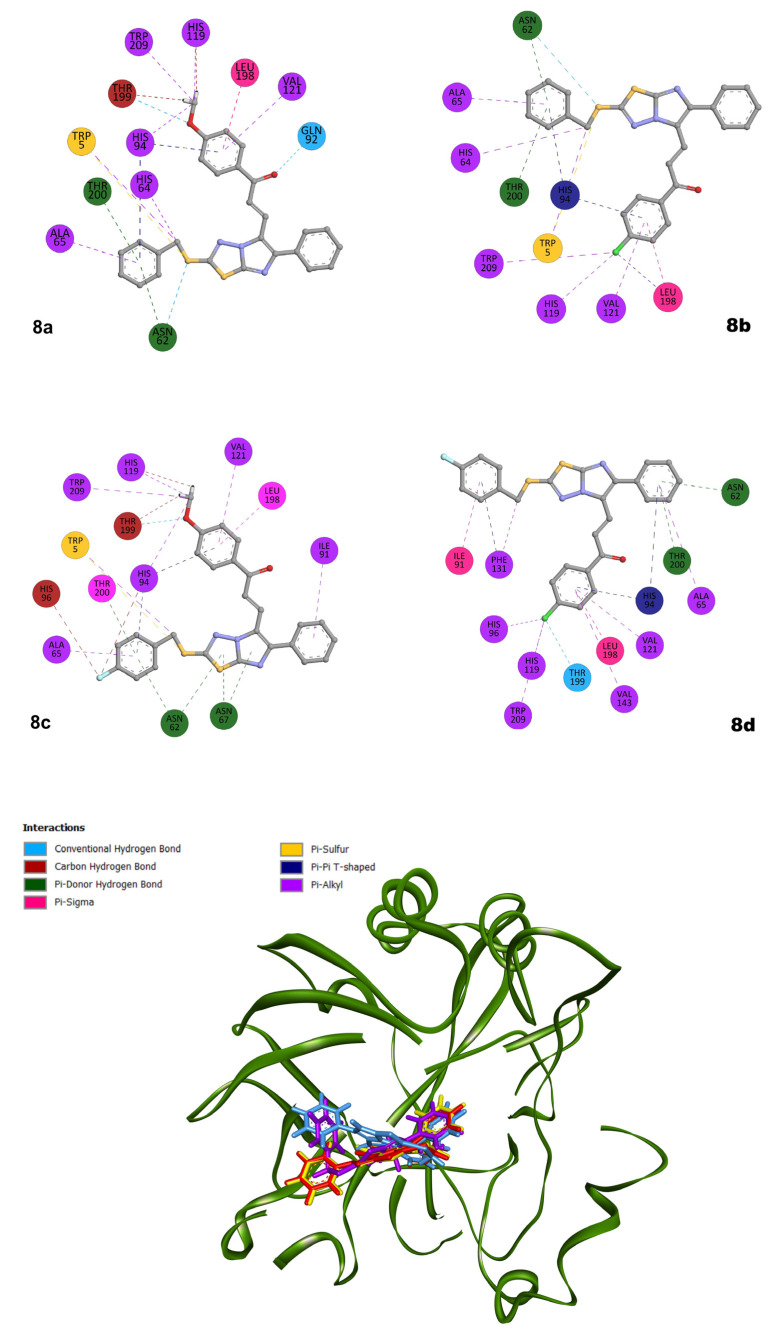
Molecular docking poses of compounds **8a–d** within the active site of hCA II.

As seen in Figure 3, the molecular docking studies with the enzyme hCA II reveal the interactions of the four novel compounds at the binding sites in detail, and these data show a remarkable correlation with the in vitro biological activity results in general. At this point, it can be said that the number of hydrogen bonds and the presence of halogen interactions and π-π bonding with aromatic residues stand out as the main structural factors determining the inhibitory activity.

When each compound was scrutinized separately, compound **8a** showed binding via π-π stacking with five different hydrogen bonds (ASN62, GLN92, THR199, HIS119, THR200) and HIS94. In addition, it can be stated that π-alkyl interactions with hydrophobic sites such as TRP5, TRP209, and HIS64 strengthened the binding position. This rich bond structure is supported by a binding energy of −11.3 kcal/mol, indicating a moderate inhibitory profile, in agreement with the Kᵢ value of 49.33 nM observed in vitro. Compound **8b** contains significant bonds, such as hydrogen bonds via ASN62 and THR200, π-sulfur interaction with TPR5, and π-π stacking with HIS94. However, it is more limited in terms of its bond diversity and number of bonds than compound **8a**. Accordingly, it is observed that this binding structure shows a significant agreement between the affinity of −10.7 kcal/mol and the Kᵢ value of 52.45 nM. For compound **8c**, it is seen that it is the compound exhibiting the most extensive binding profile with hCA II. Here, the binding pattern including five individual hydrogen bonds (THR199, HIS94, HIS96, HIS96, HIS119, ASN62), three halogen bonds (PHE70, ASP72, PHE91), and multiple π-π stacking with HIS94 is remarkable. Furthermore, it can be said that π-alkyl interactions with residues such as TRP209 and ALA95 increase the binding stability of the molecule. This rich interaction profile, with an affinity of −11.5 kcal/mol, is in agreement with the lowest Kᵢ value of 36.08 nM measured in vitro. Similarly, compound **8d** shows hydrogen bonds via GLN92 and THR199 and halogen-based interactions with PHE70, ASP72, and PHE91. The π-π stacking bonds with HIS94 and PHE131 are supported by π-alkyl interactions with TRP209 and HIS119. Accordingly, it can be said that the versatile binding properties of this compound present a structure exactly in line with its Kᵢ value of 39.57 nM, with a binding energy of −11.4 kcal/mol.

In conclusion, the molecular docking analyses performed with the hCA II enzyme are strongly consistent with the in vitro biological activities. Here, it can be inferred that, in particular, hydrogen and halogen bonds with catalytic-site residues such as THR199, HIS94, ASN62, and PHE91 play a decisive role in inhibitory activity. Accordingly, compound **8c** can be identified as the most effective inhibitor on hCA II, with a richness of structural bonding and the lowest Kᵢ value.

**Figure 4:**
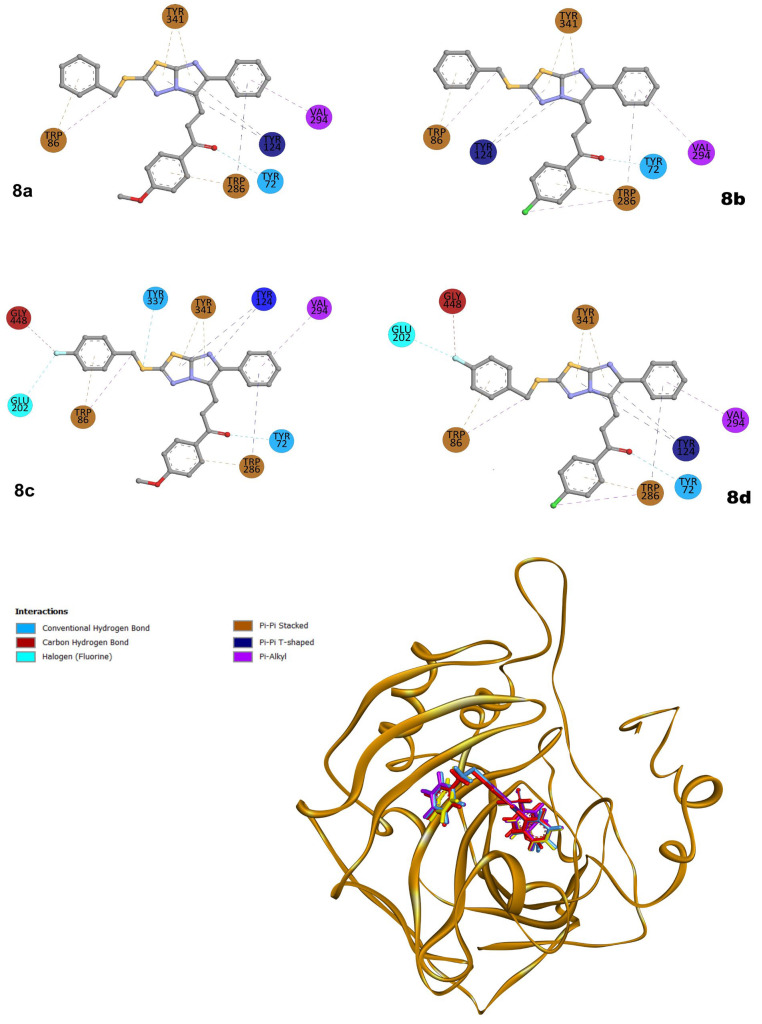
Molecular docking poses of compounds **8a–d** within the active site of AChE.

Examining the molecular interactions of the four new synthesized compounds with the AChE enzyme from Figure 4 in detail, compound **8a** stands out as the molecule that binds most strongly to the active site of AChE. In this regard, it is observed that in addition to hydrogen bonding with TYR72, it formed numerous π-π stacking and T-shaped interactions with aromatic residues in the catalytic sites of enzymes such as TRP86 and TRP286. Here, furthermore, it is seen that π-alkyl and hydrophobic contacts with TYR124, TYR341, and VAL294 increase the binding stability of the molecule. This rich and strategic binding profile has a binding affinity of −11.5 kcal/mol, and this value is in complete agreement with the molecule’s lowest Kᵢ value of 3.86 nM measured against AChE in vitro. Compound **8b** similarly establishes strong π-π stacking interactions with TRP86 and TRP286. However, herein, it can be said that the absence of hydrogen bonds and the relatively limited bond diversity slightly reduce the binding strength. It can be expressed that this is quite consistent with the affinity of −10.3 kcal/mol and the Kᵢ value of 6.01 nM. In contrast, compound **8c** has a very rich profile in terms of the bond type. In this respect, hydrogen bonds with TYR72 and TYR337, halogen bonds with GLY448 and GLU202, and stacking interactions with TRP86 and TRP286 were observed. However, it has a binding energy of −9.8 kcal/mol. The reason is that, in this situation, although the number of bonds was high, the orientation and binding geometry were not optimal, resulting in a lower than expected affinity. Moreover, this attribution can be supported by the estimated binding energy of −9.8 kcal/mol and Kᵢ value of 11.35 nM. In contrast, compound **8d** shows fewer but more efficiently positioned interactions compared to compound **8c**. It can be said that stacking with TRP86 and TRP286, halogen with GLY448, hydrogen bonding with TYR72, and hydrophobic interactions with TYR124 contribute to the structural stability of this compound. This profile is supported by an affinity of −10.2 kcal/mol and is in agreement with the Kᵢ value of 7.98 nM measured in vitro.

In summary, it can be concluded that the molecular docking analyses for the AChE enzyme reflect the experimental inhibitory potential with high accuracy. In particular, π-π stacking and T-shaped interactions with active-site residues such as TRP86 and TRP286 and the presence of hydrogen bonds stand out as the main determinants of inhibitor activity. In this framework, compound **8a** can be defined as the most potent AChE inhibitor in terms of both its structural binding properties and biological effect.

Molecular docking analyses with the BChE enzyme revealed a strong correlation between the binding behaviors of the four synthesized compounds and their in vitro biological activities.

**Figure 5:**
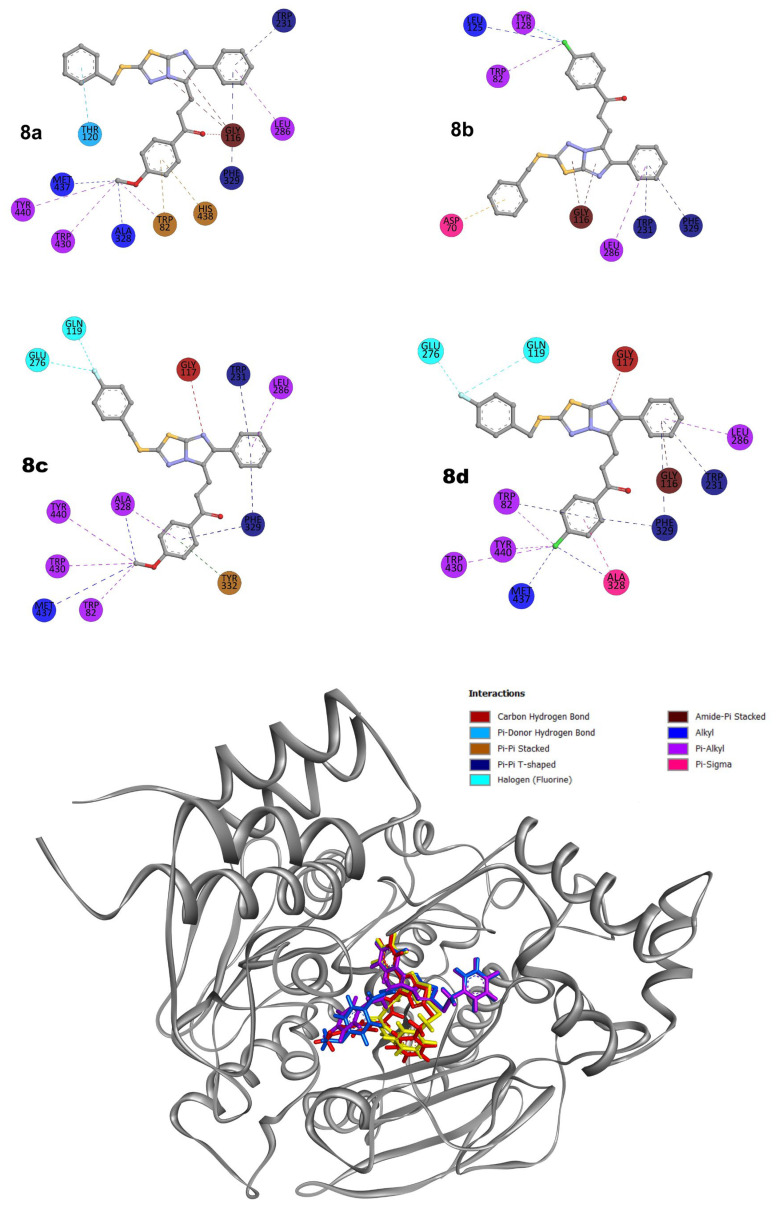
Molecular docking poses of compounds **8a–d** within the active site of BChE.

As seen in Figure 5, compound **8a** was the molecule exhibiting the strongest inhibitory profile against the BChE enzyme, with an affinity value of −11.2 kcal/mol. Also, docking analyses revealed that it formed hydrogen bonds with GLY116 and THR120, and it also showed strong π-π stacking and T-shaped interactions with TRP82, TRP231, and HIS438. Here, it can be stated that two separate amide-π stacking interactions with GLY116 and alkyl contacts with MET437 support bond stability. It has a binding energy of −11.2 kcal/mol, and it can be said that this value is highly consistent with the Kᵢ value of 1.01 nM observed in the in vitro studies. Compound **8b** establishes a halogen-based hydrogen bond via TYR128 and performs an electrostatic π-anion interaction with ASP70. It also has π-π T-shaped contacts with aromatic residues such as TRP231 and PHE329. Furthermore, it is seen that two separate amide–π stackings with GLY116 and alkyl interactions with LEU125 support the binding stability of the molecule. Compound **8b** has a binding affinity of −10.4 kcal/mol, and this binding profile coincides with an in vitro Kᵢ value of 1.70 nM. Examining compound **8c**, it shows hydrogen bonds with GLY117 and TYR332 and halogen interactions with GLN119 and GLU276, and it forms effective π-π interactions with residues such as TRP231 and PHE329. Also, the multiple π-alkyl bonds with TRP430 and TRP82 are particularly noteworthy. Here, it can be expressed that this binding pattern overlaps with the Kᵢ value of 1.56 nM, with an affinity of −10.0 kcal/mol. Compound **8d** establishes hydrogen bonds with GLY117, halogen interactions with GLN119 and GLU276, and π-π T-shaped contacts with aromatic residues such as TRP231 and PHE329. Also, amide-π stacking with GLY116 and alkyl bonds with MET437 contribute to the binding stability of the molecule. Here, it is structurally very similar to compound **8c**, and the binding affinity was similar at −10.1 kcal/mol, in agreement with the Kᵢ value of 1.78 nM measured in vitro.

The molecular docking analyses for BChE reveal a strong correlation with its in vitro inhibitory activities. In particular, π-π and π-alkyl interactions with active-site aromatic residues such as TRP82, TRP231, and PHE329 and hydrogen and stacking bonds through GLY116/117 stand out as the main determinants of the inhibitory activity. In this context, compound **8a** can be identified as the most effective BChE inhibitor in terms of both its bond diversity and positioning.

To better illustrate the binding orientations of the most active compounds, 3D representations of the predicted docking poses within the active sites of AChE, BChE, hCA I, and hCA II are also provided in Figure 2, Figure 3, Figure 4 and Figure 5. These images visualize key molecular interactions, such as hydrogen bonding and π-π stacking between the ligands and catalytic residues, providing structural context for the observed activity trends.

Despite their shared chalcone backbone, compounds **8a–d** exhibited noticeably different binding conformations across the four enzyme targets. These differences are primarily attributed to variations in the electronic distribution and the spatial orientation of the substituents, which influence their interaction with hydrophobic pockets and polar residues within the active site.

In the case of AChE, compound **8a** formed a strong hydrogen bond with the catalytic residue Ser203, along with π-π stacking interactions involving Trp86 and Phe295. Conversely, **8c** adopted a slightly different orientation, maximizing hydrophobic contacts with Tyr337, which may explain the subtle differences in the docking scores and IC_50_ values between the two compounds. For BChE, which has a wider and more flexible gorge, the binding was generally weaker; compound **8b** exhibited limited interactions with active-site residues such as Gly116, Ser198, and His438, correlating with its lower activity.

In the carbonic anhydrases hCA I and hCA II, docking revealed that the compounds positioned their chalcone moieties near critical residues such as Thr199, Glu106, and Asn62, although they adopted varied binding orientations. These differences in alignment and interaction patterns are consistent with the moderate variations in the IC_50_ values observed across the compound series.

Overall, the docking data provide valuable insights into the structure–activity relationships (SARs), emphasizing the role of substituent effects, spatial fit, and non-covalent interactions in modulating enzyme inhibition. To validate the reliability of the docking protocol, a redocking study was conducted using co-crystallized ligands. The RMSD values between the redocked and crystallographic poses were within acceptable limits (<2.0 Å), confirming the accuracy of the docking parameters. The details of this validation, including the redocked poses and RMSD values, are provided in the Supplementary Materials (Figures S43–S46).

### 2.4. MD Simulation

The binding behaviors of the four novel compounds synthesized in this study (compounds **8a–d**) against hCA I, hCA II, AChE, and BChE enzymes were evaluated by MD simulations. The RMSD, Rg, and RMSF analyses obtained from the simulations carried out for 100 ns revealed the structural compatibility and stability of the ligands with the enzymes in the dynamic process.

To further validate the dynamic effects induced by ligand binding, compound-free (WT) enzyme simulations were also conducted under identical conditions. These negative controls provided a comparative baseline to distinguish ligand-induced conformational stability from intrinsic protein fluctuations.

**Figure 6:**
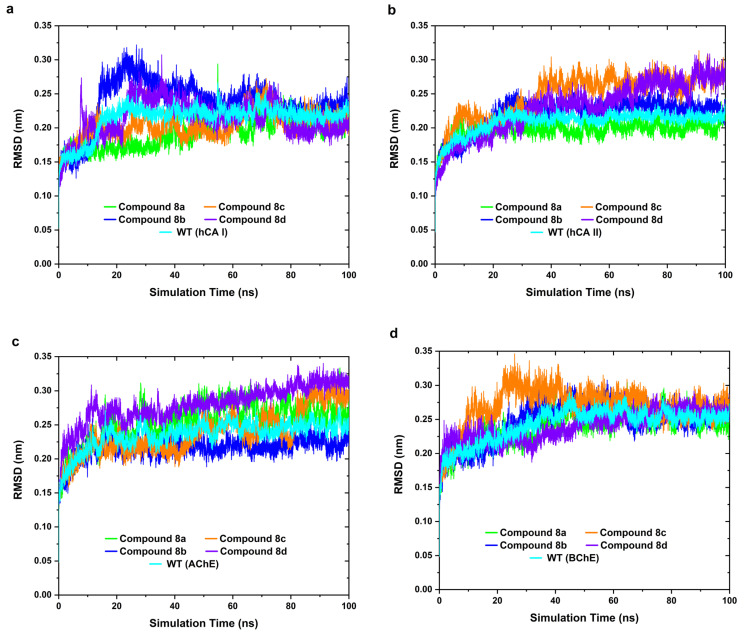
Protein RMSD profiles from 100 ns MD simulations in enzyme–ligand complexes for hCA I (**a**), hCA II (**b**), AChE (**c**), and BChE (**d**) with compounds **8a–d**, alongside their corresponding compound-free enzyme forms.

Of these, the RMSD was used to evaluate whether the protein–ligand complexes became stable over time. As seen in Figure 6, it can be said that in all the enzyme systems, a significant convergence was observed after the first 20–45 ns of the simulations. When the complexes formed with the hCA I enzyme were examined, it was seen that compound **8a** became stable in approximately 20 ns and exhibited the most stable structure with the lowest RMSD value. In the hCA II systems, similarly, the early stabilization of compound **8a** was remarkable, while this process extended up to 40 ns for compounds **8c** and **8d**. It was observed that compound **8b** reached an early equilibrium against the AChE enzyme and stood out with its low deviation value. In the BChE complexes, compound **8a** was the compound with the most stable profile not only early on but also throughout the simulation.

When compared to the apo forms of the enzymes, the ligand-bound complexes generally exhibited lower RMSD fluctuations, especially for compound **8a**, suggesting that ligand binding contributed positively to the structural stability.

These findings indicate that the structural integrity of the systems after binding was largely preserved, and the ligands developed conformations compatible with the target enzymes.

**Figure 7:**
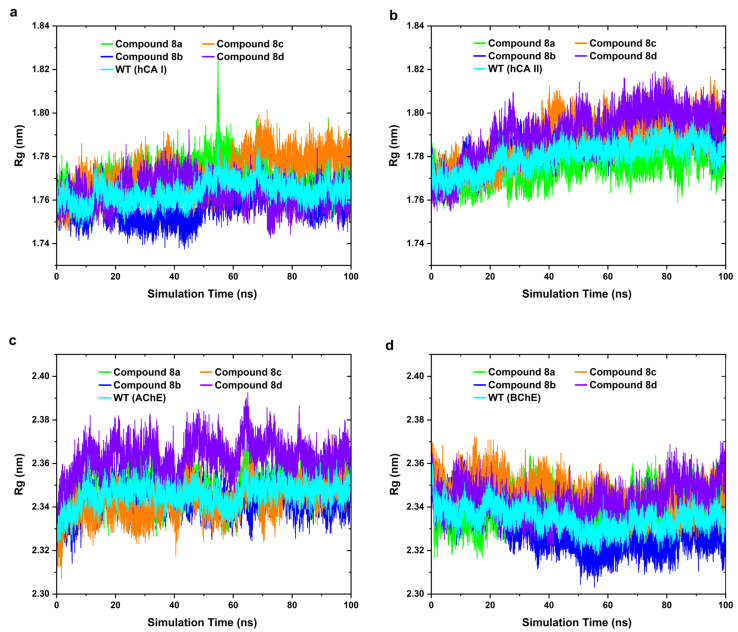
Protein Rg profiles from 100 ns MD simulations in enzyme–ligand complexes for hCA I (**a**), hCA II (**b**), AChE (**c**), and BChE (**d**) with compounds **8a–d**, alongside their corresponding compound-free enzyme forms.

Rg analyses were used to examine the compactness of the complexes and to reveal the effect of the ligand binding on the overall structural integrity of the protein. Here, the Rg values are depicted in Figure 7. Accordingly, when the hCA I systems were analyzed, the Rg values of the hCA I systems complexed with compounds **8a** and **8b** were relatively more stable and had low fluctuations in the range of 1.76–1.78 nm, while a noticeable increase was observed around 60 ns in the simulation with compound **8c**. This suggests that compound **8c** may temporarily affect the compactness of the enzyme after binding. The results obtained with compound **8d** show a significant increase and fluctuate with time, and this situation reflects the lowest profile in terms of stability. In this context, compound **8d**, which was identified as the most effective compound on hCA I in the docking data, appears to form a less compact structure in terms of structural stability in the dynamic process despite its strong binding profile. This finding may indicate that there is a balance between the flexibility of the complex and its strong binding profile.

The most compact structure in terms of the hCA II complexes was obtained with compound **8a**, and this structure became more stable over time. This situation, in line with the docking results, shows that this compound binds effectively and stably to the hCA II surface. For compounds **8c** and **8d**, the Rg values are higher, and the fluctuations in compound **8d** are more pronounced. This state shows that these compounds cause some flexibility in the structure after binding to the enzyme. In contrast, examining the simulations for the AChE enzyme complexes, the Rg values of all the compounds generally remained in the range of 2.32–2.38 nm. Compound **8d** showed the highest Rg value in this enzyme. This situation reveals that compound **8d**, which showed relatively less bond structure in the docking analyses, is weak in forming a compact structure on AChE, while the Rg values in the complexes with compounds **8a** and **8b** were relatively lower and stable. Here, especially when considering both the docking scores and the low Rg value of compound **8a**, it can be said that it binds stably and tightly to the AChE surface. From the results for the BChE, the Rg values of all the compounds were quite close and had low fluctuations. Here, it was observed that the complex with compound **8a** exhibited the most compact structure, and this situation indicates that this compound is the most effective inhibitor against the BChE enzyme both structurally and functionally. Also, it can be said in general that the docking scores and in vitro K_i_ values support these data.

**Figure 8:**
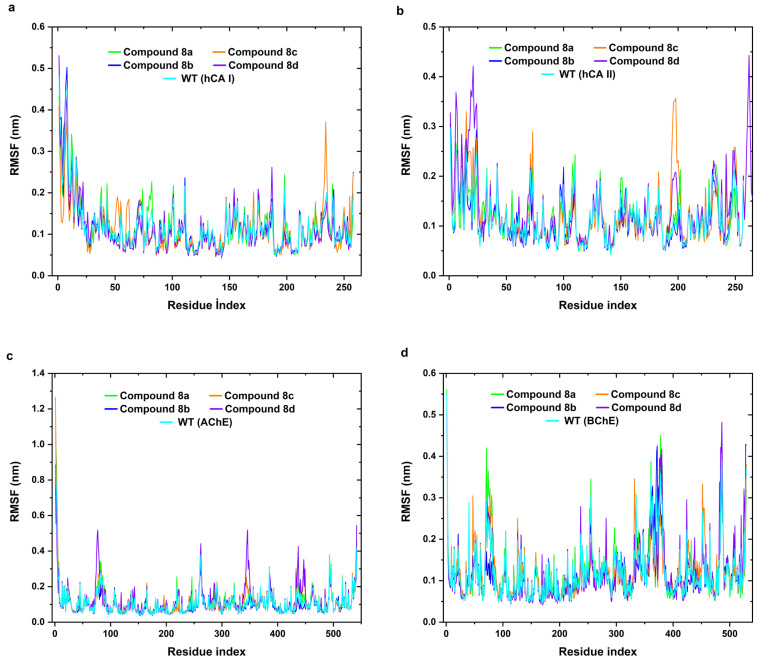
Protein RMSF profiles from 100 ns MD simulations in enzyme–ligand complexes for hCA I (**a**), hCA II (**b**), AChE (**c**), and BChE (**d**) with compounds **8a–d**, alongside their corresponding compound-free enzyme forms.

Finally, RMSF analysis was performed to evaluate how ligand binding affects local mobility by revealing the flexibility profiles of proteins at the amino acid level (Figure 8). This analysis provided the opportunity to examine the structural changes in the binding sites and surroundings of the complexes formed against the four enzymes in more detail. In addition to ligand-bound systems, compound-free enzyme trajectories were also analyzed to contextualize the fluctuation patterns.

Overall, low-level fluctuations were observed in the complexes in the hCA I system, and the RMSF values were mostly in the range of 0.10–0.25 nm. Also, it is seen that although compound **8c** exhibited a slightly increased flexibility for some amino acids in the N- and C-terminal regions, this local mobility did not affect the overall stability of the system. In addition, in the simulations performed with compounds **8a**, **8b**, and **8d**, very low fluctuations are noted, especially in the residues around the active site. This finding supports the fact that these compounds, which established many hydrogen and π-π interactions in the active site in the docking analyses, remained stable in the position where they were bound throughout the simulation.

Similar RMSF profiles were obtained for all the hCA II complexes, and the fluctuations in their RMSF values generally remained in the range of 0.10–0.20 nm. Except for the slightly increased flexibility in the 180–200th residue range of compound **8c**, all the systems exhibited very stable structures. The complex with compound **8a** had the lowest RMSF curve in all the residue regions, and these observations suggest a situation parallel to the high binding score and multiple interactions obtained in the docking study. Moreover, this low fluctuation observed in the residues corresponding to the active site indicates that the ligand maintains its structural stability by maintaining its position in the binding site.

In the simulations with AChE, although significant RMSF increases were observed in the superficial regions (~250, ~400, ~500 residues), it can be said that these increases mostly occurred in the flexible loop regions located outside the binding site. At this point, it is seen that the mobility of compound **8d** in these regions is relatively higher. In contrast, the low RMSF profile exhibited by compound **8b** around the active site is structurally consistent with the strong π-π interactions and high binding scores observed on AChE in the docking analyses. This state may indicate that the ligand maintained its position on the target site and the complex remained stable throughout the simulation.

The RMSF values obtained in the BChE complexes generally remained between 0.10 and 0.30 nm, and no significant deviations were observed. The complex with compound **8a** attracts attention since it reveals relatively lower flexibility, especially in the 200–300th residue range. In contrast, the complex with compound **8c** exhibits relatively higher fluctuations in the same region. In summary, it can be said that compound **8a**, which stands out as the strongest BChE inhibitor in the docking analyses, has the lowest RMSF value trend here, and these outputs reveal a significant correlation in terms of both the binding energy and dynamic stability.

As a result, it can be said that the MD simulations show that all the compounds established compatible and balanced interactions with enzyme surfaces. When the RMSD, Rg, and RMSF analyses are evaluated together, it is inferred that compound **8a**, especially, exhibits a structurally stable and effective profile against multiple enzymes both during and after binding. These results are in high agreement with the docking data and in vitro biological activities; they also support the potential inhibitory properties of the compounds at a dynamic level.

### 2.5. In Silico Pharmacokinetic and Toxicological Evaluation

The pharmacokinetic and toxicological profiles of the four new synthesized compounds were analyzed in silico using the pkCSM platform. This comprehensive assessment reveals the bioavailability potential, central nervous system penetration, metabolic stability, and safety of each molecule. With this in mind, our aim is that these parameters are interpreted within the framework of their relationships with biological targets, allowing for a multidimensional assessment of the therapeutic potential. In this regard, the relevant parameters are listed in Table 4. Accordingly, it can be said that all the compounds show high oral bioavailability, since the human intestinal absorption rates are over 85%. However, significant differences were observed in terms of the penetration into the central nervous system. In particular, compound **8b** (logBB: 0.383) and compound **8d** (logBB: 0.506) showed permeability at a level that can cross the brain–blood barrier, which makes them suitable for therapeutic applications for central nervous system-targeted enzymes such as AChE/BChE and hCA II. In contrast, it can be predicted that compounds such as compound **8a** and compound **8c** have lower BBB permeability, and therefore they can be potentially targeted at enzyme targets.

When examined from a metabolic perspective, interestingly, the ADMET analyses revealed that the synthesized compounds exhibited substrate and inhibitory properties for both CYP3A4 and P-glycoprotein (P-gp). This multifaceted interaction offers significant advantages in terms of pharmacokinetics and pharmacodynamics. These compounds, which undergo metabolic destruction as CYP3A4 substrates, can also slow down their own metabolism by inhibiting this enzyme. This situation may prolong their half-life and enhance their bioavailability. Similarly, the bidirectional interaction with P-gp is especially valuable in terms of multiple drug resistance (MDR). While they can pass through biological barriers (e.g., intestinal epithelium, blood–brain barrier) as P-gp substrates, they can suppress the active drug excretion of this transporter thanks to their inhibitory properties. This characteristic can help overcome MDR by allowing drugs to remain in the cell for a longer period of time. This dual regulatory effect on both CYP3A4 and P-gp is promising in terms of optimizing systemic exposure, increasing therapeutic efficacy, and reducing interindividual differences. However, this situation requires the careful evaluation of the compounds in terms of potential drug–drug interactions.

When the compounds were examined from a toxicological perspective, the hepatotoxicity and skin sensitization risks were found to be negative in all four molecules, which contributes positively to the overall safety profile. However, compound **8a** was identified as a hERG channel inhibitor, which is a preliminary warning in terms of cardiotoxicity risk. Compounds **8b** and **8d** produced positive results in terms of the AMES test; therefore, they stand out as candidates that should be carefully evaluated in terms of their mutagenicity potential.

In summary, in light of the pharmacokinetic and toxicological data, all four compounds show positive profiles in terms of high absorption and liver safety. Also, it can be said that compounds **8b** and **8d** in particular are more suitable for targeting the central nervous system, while compound **8c** stands out for its therapeutic potential towards peripheral targets. In this context, the pharmacokinetic and toxicological evaluation, in line with the biological activity data, supports the clinical relevance and development potential of the molecules.

## 3. Materials and Methods

### 3.1. Experimental Synthesis 

#### 3.1.1. General Methods

^1^H NMR and ^13^C NMR analyses of the synthesized compounds were carried out using Agilent Annual Refill (400 MHz). Chemical shift values in the ^1^H NMR spectra are given as ppm. Observed signals are expressed as follows: s: singlet; bs; broad singlet; d: doublet; dd: doublet of doublet; t: triplet; q: quartet; m: multiplet. Mass spectra were obtained using the ESI (+) method with a Thermo Scientific Q Exactive device. Elemental analyses were carried out on a LECO 932 CHNS device. FT-IR spectra of the compounds were recorded in ATR using a Thermo Scientific Nicolet iS5 device. Melting points were determined using a Thermo Scientific IA9000 device. Thin-layer chromatography (TLC) was carried out with silica gel 60 F_254_ aluminum TLC plates, and the spots were determined under UV light (254 nm).

##### General Synthesis of 2-amino-1,3,4-thiadiazole Derivatives (**3a–b**)

The starting compounds of the study (**3a–b**) were synthesized according to the literature [34,35,36,37,56].

##### General Synthesis of 2,6-disubstituted imidazo[2,1-*b*][1,3,4]thiadiazole Derivatives (**5a–d**)

Compounds **5a–b** were synthesized according to the literature [34,35,36,37,56].

##### General Synthesis of 2-(substituted-benzylthio)-6-phenylimidazo[2,1-*b*][1,3,4]thiadiazole-5-carbaldehyde Derivatives (**6a–b**)

DMF (11.01 mL, 0.1422 mol) was put into a 250 mL flask and brought to 0 °C with an ice bath. POCl_3_ (0.0278 mol, 2.55 mL) was added slowly with continuous stirring. Thus, fresh Vilsmeier–Haack reagent was prepared. Then, compounds **5a–b** (0.00618 mol) were added to the mixture, and the reaction continued at 0 °C for 30 min. Then, the reaction continued at 25 °C for 2 h and at 60 °C for 2 h. Finally, 50 mL of 5% Na_2_CO_3_ was added to the reaction, and the reaction was continued for another 2 h at 90 °C. At the end of this period, the mixture was cooled to room temperature. Then, it was extracted with water and dichloromethane. The solvent was evaporated in the evaporator. The crude substance was recrystallized from dichloromethane.

2-(Benzylthio)-6-phenylimidazo[2,1-*b*][1,3,4]thiadiazole-5-carbaldehyde (**6a**)

Yellow solid (2.07 g, 95%), mp. 108–109 °C. FT-IR (ATR) ν_max_: 3026 (Ar-CH), 2941 (Aliph. CH), 1671 (C=O), 1598 (C=N); ^1^H NMR (400 MHz, CDCl_3_), δ (ppm): 10.05 (s, 1H), 7.89–7.81 (m, 2H), 7.49–7.43 (m, 4H), 7.36 (td, *J* = 5.8, 2.7 Hz, 2H), 7.33–7.24 (m, 2H), 4.55 (s, 2H); ^13^C NMR (101 MHz, CDCl_3_) δ (ppm): 177.35, 162.39, 155.57, 150.63, 134.73, 132.20, 129.81, 129.46, 129.08, 128.89, 128.73, 128.33, 124.14, 38.64; Anal. (% calculated/found) for C_18_H_13_N_3_OS_2_ (MW: 351.44) C: 61.52/61.44; H: 3.73/3.82; N: 11.96/11.82; LC-MS/MS (ESI-*m/z*): 352.05 (M + 1, 100).

2-((4-Fluorobenzyl)thio)-6-phenylimidazo[2,1-*b*][1,3,4]thiadiazole-5-carbaldehyde (**6b**)

Yellow solid (2.12 g, 93%), mp. 85–86 °C. FT-IR (ATR) ν_max_: 3059 (Ar-CH), 2979 (Aliph. CH), 1640 (C=O), 1598 (C=N); ^1^H NMR (400 MHz, CDCl_3_), δ (ppm): 10.04 (s, 1H), 7.83 (dq, *J* = 6.1, 2.1 Hz, 2H), 7.49 (dtd, *J* = 8.6, 6.2, 1.8 Hz, 5H), 7.08–6.98 (m, 2H), 4.54 (s, 2H); ^13^C NMR (101 MHz, CDCl_3_) δ (ppm): 177.35, 163.78, 162.04, 161.32, 155.74, 150.64, 132.16, 131.36, 131.28, 130.66, 130.63, 129.84, 129.09, 128.87, 128.74, 125.01, 124.18, 115.93, 115.72, 37.78; Anal. (% calculated/found) for C_18_H_12_FN_3_OS_2_ (MW: 369.43) C: 58.52/58.44; H: 3.27/3.38; N: 11.37/11.32; LC-MS/MS (ESI-*m*/*z*): 370.04 (M + 1, 100).

##### General Synthesis of Chalcone Derivatives (**8a–d**)

Amounts of 10 mL of ethanol and 2 mL of 10% NaOH were stirred in an ice bath for a few minutes. Then, methyl ketone derivatives (**7a–b**) (0.853 mmol) were added to the mixture. Then, aldehyde derivatives (**5a–b**) (0.853 mmol) were added to the reaction mixture in portions. The reaction continued in an ice bath for 30 min. At the end of this period, the reaction was taken to room conditions and continued for 8–24 h. The progress of the reaction was controlled by TLC. When the reaction was completed, the substance in the flask was poured into the ice bath, and 1–2 mL of acetic acid was added. When precipitation occurred, the solid formed was filtered, washed with water, and dried under vacuum effect. Then, the substance was recrystallized from ethanol. Various physical properties and spectral data of the target compounds are given below.

(*E*)-3-(2-(Benzylthio)-6-phenylimidazo[2,1-*b*][1,3,4]thiadiazol-5-yl)-1-(4-methoxyphenyl)prop-2-en-1-one (**8a**)

Yellow solid (0.29 g, 71%), mp. 136–138 °C. *R_f_* = 0.52 (CHCl_3_). FT-IR (ATR) ν_max_: 3060 (Ar-CH), 2934 (Aliph. CH), 1647 (C=O), 1599 (C=N); ^1^H NMR (400 MHz, CDCl_3_), δ (ppm): 8.07–7.99 (m, 3H), 7.72 (d, *J* = 7.6 Hz, 2H), 7.60 (d, *J* = 14.5 Hz, 1H), 7.45 (t, *J* = 10.3 Hz, 6H), 7.35 (d, *J* = 15.1 Hz, 2H), 6.93 (d, *J* = 8.4 Hz, 2H), 4.58 (s, 2H), 3.86 (s, 3H); ^13^C NMR (101 MHz, CDCl_3_) δ (ppm): 188.34, 174.82, 163.33, 160.59, 150.17, 135.48, 134.90, 133.34, 131.31, 130.89, 129.46, 129.16, 128.97, 128.89, 128.66, 127.68, 125.38, 122.29, 121.65, 119.74, 55.48, 38.79; Anal. (% calculated/found) for C_27_H_21_N_3_O_2_S_2_ (MW: 483.60) C: 67.06/67.21; H: 4.38/4.45; N: 8.69/8.52; LC-MS/MS (ESI-*m*/*z*): 484.11 (M + 1, 100).

(*E*)-3-(2-(Benzylthio)-6-phenylimidazo[2,1-*b*][1,3,4]thiadiazol-5-yl)-1-(4-chlorophenyl)prop-2-en-1-one (**8b**)

Yellow solid (0.32 g, 76%), mp. 144–146 °C. *R_f_* = 0.41 (CHCl_3_); FT-IR (ATR) ν_max_: 3064 (Ar-CH), 2983 (Aliph. CH), 1649 (C=O), 1589 (C=N); ^1^H NMR (400 MHz, CDCl_3_), δ (ppm): 8.06 (d, *J* = 3.4 Hz, 2H), 7.94 (d, *J* = 8.1 Hz, 2H), 7.71 (d, *J* = 14.5 Hz, 2H), 7.52–7.33 (m, 8H), 7.32 (d, *J* = 16.1 Hz, 2H), 4.56 (s, 2H); ^13^C NMR (101 MHz, CDCl_3_) δ (ppm): 188.65, 160.86, 150.93, 148.77, 139.05, 136.72, 134.80, 133.20, 129.76, 129.28, 129.14, 129.00, 128.92, 128.84, 128.68, 128.63, 128.46, 121.44, 118.97, 38.82; Anal. (% calculated/found) for C_26_H_18_ClN_3_OS_2_ (MW: 488.02) C: 63.99/63.82; H: 3.72/3.84; N: 8.61/8.73; LC-MS/MS (ESI-*m*/*z*): 488.06 (M^+^, 100).

(*E*)-3-(2-((4-Fluorobenzyl)thio)-6-phenylimidazo[2,1-*b*][1,3,4]thiadiazol-5-yl)-1-(4-methoxyphenyl)prop-2-en-1-one (**8c**)

Yellow solid (0.34 g, 79%), mp. 127–128 °C. *R_f_* = 0.43 (CHCl_3_); FT-IR (ATR) ν_max_: 3066 (Ar-CH), 2933 (Aliph. CH), 1644 (C=O), 1599 (C=N); ^1^H NMR (400 MHz, CDCl_3_), δ (ppm): 8.06–7.98 (m, 3H), 7.65–7.58 (m, 3H), 7.54–7.36 (m, 4H), 7.26 (d, *J* = 15.6 Hz, 1H), 7.26 (s, 2H), 7.03 (t, *J* = 8.5 Hz, 2H), 4.55 (s, 2H), 3.87 (s, 3H); ^13^C NMR (101 MHz, CDCl_3_) δ (ppm): 188.39, 176.61, 174.56, 163.35, 150.20, 140.11, 133.27, 131.26, 130.96, 130.82, 130.74, 130.66, 129.22, 129.08, 128.87, 128.71, 128.66, 128.16, 125.30, 122.27, 121.65, 119.81, 116.15, 115.93, 115.64, 115.42, 113.83, 55.49, 38.06; Anal. (% calculated/found) for C_27_H_20_FN_3_O_2_S_2_ (MW: 501.59) C: 64.65/64.52; H: 4.02/4.14; N: 8.38/8.30; LC-MS/MS (ESI-*m*/*z*): 502.10 (M + 1, 100).

(*E*)-1-(4-Chlorophenyl)-3-(2-((4-fluorobenzyl)thio)-6-phenylimidazo[2,1-*b*][1,3,4]thiadiazol-5-yl)prop-2-en-1-one (**8d**)

Yellow solid (0.32 g, 70%), mp. 129–130 °C. Rf = 0.37 (CHCl_3_); FT-IR (ATR) ν_max_: 3062 (Ar-CH), 2930 (Aliph. CH), 1648 (C=O), 1589 (C=N); ^1^H NMR (400 MHz, CDCl_3_), δ (ppm): 8.04 (s, 2H), 7.94 (d, *J* = 8.2 Hz, 2H), 7.71 (d, *J* = 14.5 Hz, 2H), 7.42 (t, *J* = 7.5 Hz, 6H), 7.17 (s, 1H), 7.03 (t, *J* = 8.4 Hz, 2H), 4.53 (s, 2H); ^13^C NMR (101 MHz, CDCl_3_) δ (ppm): 188.70, 163.84, 161.37, 160.54, 150.94, 148.69, 139.09, 136.70, 133.15, 130.81, 130.72, 130.66, 130.59, 130.55, 129.73, 129.30, 129.14, 128.88, 128.68, 128.43, 121.43, 119.06, 116.17, 115.95, 115.64, 115.42, 38.11; Anal. (% calculated/found) for C_26_H_17_C_l_FN_3_OS_2_ (MW: 506.01) C: 61.72/61.59; H: 3.39/3.45; N: 8.30/8.21; LC-MS/MS (ESI-*m*/*z*): 506.05 (M^+^, 100).

### 3.2. Biological Activity

#### 3.2.1. Activities of CA I and II Isoenzymes

The activities of the CA isoenzymes were measured in accordance with Verpoorte et al.’s [57] previously described methodology. A spectrophotometer (Beckman Coulter, Germany) was used to measure the change in absorbance at 348 nm over a 30 min period at 25 °C in order to assess the hydrolysis of *p*-nitrophenylacetate. There was 400 µL of Tris–HCl buffer (pH 7.4), 360 µL of PNA (3.0 mM), 220 µL of H_2_O, and 20 µL of purified hCA isoenzyme in the reaction mixture [58]. The identical cuvette was prepared without the enzyme in order to obtain a control measurement. The standard protein was bovine serum albumin. With the exception of CA isoenzymes, all solutions used to determine enzymatic activity were included in the negative control. The positive control for hCA I and II isoenzymes was acetazolamide (AZA).

#### 3.2.2. AChE and BChE Activities

The previously published Ellman’s [59] technique was used to measure the BChE and AChE activities. AChE (C2888-500UN) from *Electrophorus electricus* and BChE (C1057-1KU), extracted from equine serum, were both obtained from Sigma-Aldrich Chemie, St. Louis, MO, USA. The BChE/AChE activities were measured using 5,5′-dithiobis(2-nitrobenzoic acid) (DTNB) and butyrylthiocholine iodide (BChI)/acetylcholine iodide (AChI). In short, 10 mL of the sample solutions with varying concentrations was dissolved in deionized water along with 100 µL of buffer (Tris–HCl, 1.0 M, pH 8.0). After adding 50 µL of BChE/AChE (5.32 × 10^−3^ EU) solution, the mixture was incubated at 25 °C for 10 min. A fraction of DTNB (50 µL, 0.5 mM) was added following incubation. Finally, 50 µL of BChI/AChI (10/10 mM) was added to initiate the reaction. The production of the yellow 5-thio-2-nitrobenzoate anion at 412 nm indicated the enzymatic hydrolysis of both substrates [60]. All enzymatic activity determination solutions, except AChE/BChE, were included in the negative control. The standard drug for the BChE and AChE enzymes was tacrine (TAC).

#### 3.2.3. IC_50_ and K_i_ Studies for Both Enzymes

To determine the IC_50_ values of the tested compounds, enzyme activity measurements were conducted at five to six different inhibitor concentrations. Percent activity values were calculated for each dose, taking into account the compounds’ molecular weights and dilution factors.

The IC_50_ study was completed prior to the Kᵢ determination. For the Kᵢ analysis, three selected concentrations of each inhibitor were used alongside a control (uninhibited) reaction. Enzyme activity data were converted to 1/V values and plotted against 1/[S] to generate Lineweaver–Burk plots.

Kᵢ values and standard deviations were calculated based on the type of inhibition (competitive or non-competitive), as inferred from the intersection pattern of the Lineweaver–Burk lines. These kinetic parameters allowed for further insight into the binding affinities and inhibition mechanisms of the compounds.

### 3.3. In Silico Studies

To evaluate the molecular basis of the enzyme-inhibitor interactions, a series of in silico studies, including molecular docking and MD simulations, were carried out. All computational analyses were designed to correlate with the in vitro inhibition profiles of the synthesized imidazothiadiazole–chalcone hybrid compounds.

#### 3.3.1. Molecular Docking Setups

The crystal structures of the target enzymes human carbonic anhydrase I (hCA I, PDB ID: 1AZM [61]), carbonic anhydrase II (hCA II, PDB ID: 3HS4 [62]), acetylcholinesterase (AChE, PDB ID: 7XN1 [63]), and butyrylcholinesterase (BChE, PDB ID: 4BDS [64]) were retrieved from the RCSB Protein Data Bank. Prior to docking, all crystallographic water molecules, ions, and co-crystallized ligands were removed using Discovery Studio Visualizer v21.1.0 [65]. Missing amino acid residues were completed by aligning sequences with their UniProt [66] and were modeled via MODELLER 10.3 [67] when necessary. Polar hydrogens were added, and Kollman charges were assigned using AutoDock Tools 1.5.7 [68].

The three-dimensional structures of the ligands were prepared using Avogadro 1.2.0, and their geometries were optimized using the MMFF94 force field [69], allowing for full torsional flexibility. The ligands were saved in PDB format and converted to PDBQT files after assigning Gasteiger charges and defining rotatable bonds in AutoDock Tools.

Molecular docking simulations were carried out in AutoDock Vina 1.2.0 [70] via the PyRx 0.8 interface [71]. For each protein, the grid box was centered around the active site, covering key catalytic residues. The box dimensions were uniformly set to 25 × 25 × 25 Å, and the exhaustiveness parameter was increased to 32 to ensure the thorough sampling of flexible ligand conformations.

Docking scores (binding affinities in kcal/mol) were recorded for each ligand–protein complex, and the top-scoring poses were selected for MD simulations. All docking simulations were conducted in triplicate to ensure consistency.

To validate the reliability of the docking protocol, a redocking study was conducted by redocking the co-crystallized ligands into the active sites of their corresponding target enzymes using the same docking parameters. The RMSD values between the redocked and crystallographic poses were calculated and found to be within acceptable limits (<2.0 Å), indicating good agreement. Details on these redocking validations and the resulting poses are provided in the Supplementary Materials (Figures S43–S46).

#### 3.3.2. Sequence Origin and Conservation Analysis

To address potential discrepancies between the enzyme origins used in the in vitro assays and those employed in molecular modeling, we conducted sequence alignment and conservation analyses. These comparisons were performed using Clustal Omega version 1.2.4, a widely used multiple sequence alignment tool, and the results are included in Supplementary Figures S41 and S42.

For AChE, the in vitro enzyme source was Electrophorus electricus (Sigma-Aldrich, Cat# C2888), whereas the computational analyses were based on the human AChE crystal structure (PDB ID: 7XN1, UniProt: P22303). Pairwise alignment revealed 76.9% sequence identity and 88.3% positives. Importantly, the catalytic triad residues (Ser203, Glu334, His447) and critical binding residues such as Trp86, Phe295, and Tyr337 were fully conserved between the two species.

For BChE, the experimental enzyme was derived from Equus caballus (horse serum, UniProt: Q9N1N9), while the human structure (UniProt: P06276) was used in the docking studies. The alignment showed a 90.1% identity and 93.4% positives, with full conservation of the catalytic triad (Ser198, Glu325, His438) and essential residues, including Trp231 and Phe329.

In the case of the CA isoforms hCA I and II, both experimental and computational studies utilized the same human recombinant enzymes, with crystal structures obtained from PDB entries 1AZM (hCA I) and 3HS4 (hCA II). Thus, no sequence variation exists. Moreover, all the key zinc-binding and catalytic residues, including His94, His96, His119, Thr199, and Glu106, were preserved.

These findings collectively demonstrate that the key active-site residues were conserved across all enzyme sources used, supporting the functional relevance and reliability of the computational modeling results.

#### 3.3.3. MD Simulations

To investigate the dynamic stability and conformational behavior of the most active compounds in the complex with their respective targets, 100 ns MD simulations were executed using GROMACS 2021.2 [72]. Initial protein–ligand complex structures were obtained from the top-ranked molecular docking poses. Protein–ligand topologies were generated using the CHARMM36 force field [73], and each complex was placed in a cubic simulation box with a 10 Å buffer, solvated using TIP3P water molecules [74]. The systems were neutralized by adding Na^+^ and Cl^−^ ions at a 0.15 M salt concentration, yielding a total of approximately 55,000–65,000 atoms per system, depending on the protein size.

Energy minimization was carried out using the steepest descent, followed by equilibration under NVT and NPT ensembles for 2 ns each. Production simulations were conducted under NPT conditions at 310 K using the v-rescale thermostat [75] and Parrinello–Rahman barostat [76].

The structural behaviors of the wild-type systems were evaluated using RMSD, Rg, and RMSF analyses, using the built-in analysis tools in GROMACS. The comparison with compound-bound systems revealed that the WT enzymes generally maintained more stable trajectories, thereby validating the significance of the ligand-induced structural fluctuations observed in complexed systems.

#### 3.3.4. ADMET Calculations

The in silico ADMET properties of the synthesized compounds (**8a–d**) were predicted using the pkCSM pharmacokinetics web server [77]. In this context, canonical SMILES strings of each compound were used as input, and accordingly, the parameters were evaluated.

## 4. Conclusions

In this study, a novel series of imidazothiadiazole–chalcone hybrid derivatives (compounds **8a–d**) was successfully synthesized using a multi-step synthetic route involving Vilsmeier–Haack and Claisen–Schmidt reactions. Structural elucidation of the target molecules was confirmed by a comprehensive set of spectroscopic and analytical techniques, including ^1^H NMR, ^13^C NMR, FT-IR, mass spectrometry, and elemental analysis.

Biological evaluations revealed that all four compounds exhibited strong inhibition against the key metabolic enzymes (AChE, BChE, hCA I, and hCA II) with Ki values in the low nanomolar range. Among these, compound **8d** showed the most potent inhibition of hCA I (K_i_ = 45.13 nM), compound **8c** was the most effective against hCA II (K_i_ = 36.08 nM), and compound **8a** emerged as the most promising dual cholinesterase inhibitor (AChE K_i_ = 3.86 nM; BChE K_i_ = 1.01 nM), surpassing the clinical reference tacrine in potency.

Molecular docking studies provided insight into the binding modes and key interactions within the active sites of the enzymes, while 100 ns MD simulations confirmed the structural stability and conformational adaptability of the ligand–enzyme complexes under physiological conditions. Furthermore, in silico pharmacokinetic and toxicity assessments indicated favorable drug-likeness properties for all the compounds, with high intestinal absorption and non-hepatotoxicity. However, some derivatives exhibited mild mutagenicity (AMES+) and hERG-I inhibition risks, suggesting that further structural refinement may be required before in vivo applications.

Overall, this study presents the first systematic investigation of imidazothiadiazole–chalcone hybrids as dual inhibitors targeting both CAs and cholinesterases. The integration of experimental and computational approaches supports their potential as lead candidates for the development of multi-target agents, particularly in the treatment of neurodegenerative and ophthalmic disorders, such as Alzheimer’s disease and glaucoma.

## Figures and Tables

**Table 1:** Substituent patterns for compounds **8a**–**8d**.

Compound	R^1^	R^2^
**8a**	-H	4-OMe
**8b**	-H	4-Cl
**8c**	-F	4-OMe
**8d**	-F	4-Cl

**Table 2:** The enzyme inhibition results of novel compounds against hCA I, hCA II, AChE, and BChE enzymes.

Compound	IC_50_ Values (nM)								K_i_ Values (nM)			
	hCA I	r^2^	hCA II	r^2^	AChE	r^2^	BChE	r^2^	hCA I	hCA II	AChE	BChE
**8a**	73.05	0.983	41.07	0.947	4.36	0.945	1.37	0.937	81.24 ± 4.36	49.33 ± 5.03	3.86 ± 0.20	1.01 ± 0.08
**8b**	64.37	0.936	49.32	0.924	6.77	0.929	1.95	0.902	78.01 ± 5.48	52.45 ± 6.24	6.01 ± 0.35	1.70 ± 0.10
**8c**	42.16	0.989	30.21	0.983	13.04	0.993	2.03	0.915	48.36 ± 6.98	36.08 ± 5.36	11.35 ± 1.02	1.56 ± 0.06
**8d**	39.14	0.961	34.08	0.940	9.30	0.946	1.98	0.938	45.13 ± 3.91	39.57 ± 4.91	7.98 ± 0.60	1.78 ± 0.12
AZA *	129.03	0.907	102.55	0.936	-	-	-	-	145.73 ± 8.03	112.63 ± 6.87	-	-
TAC *	-	-	-	-	20.34	0.968	4.35	0.905	-	-	14.27 ± 2.03	3.57 ± 0.35

* Tacrine and AZA are reference inhibitors for cholinesterase and CA, respectively.

**Table 3:** Molecular docking scores and IC_50_ values of compounds **8a**–**d** against hCA I, hCA II, AChE, and BChE enzymes.

Compound	Docking Score (AChE) [kcal/mol]	IC_50_ (AChE) [μM]	Docking Score (BChE) [kcal/mol]	IC_50_ (BChE) [μM]	Docking Score (hCA I) [kcal/mol]	IC_50_ (hCA I) [μM]	Docking Score (hCA II) [kcal/mol]	IC_50_ (hCA II) [μM]
**8a**	−10.2	2.8	−9.5	5.6	−9.1	2.41 ± 0.11	−9.3	3.18 ± 0.15
**8b**	−9.8	4.1	−8.9	7.3	−8.9	3.05 ± 0.13	−9.2	3.97 ± 0.20
**8c**	−10.0	3.5	−9.7	6.0	−9.0	2.87 ± 0.10	−9.1	3.45 ± 0.17
**8d**	−9.6	5.0	−9.1	7.8	−8.7	3.40 ± 0.16	−9.0	4.12 ± 0.21

**Table 4:** Predicted pharmacokinetic and toxicological parameters of compounds **8a–d** calculated using the pkCSM online platform.

Characteristic	Compound 8a	Compound 8b	Compound 8c	Compound 8d
Intestinal absorption (%)	87.03	85.52	86.50	84.99
BBB permeability (logBB)	0.079	0.383	0.202	0.506
CNS permeability (logPS)	–1.76	–0.95	–1.83	–0.98
AMES toxicity	No	Yes	No	Yes
hERG I inhibition	Yes	No	No	No
Hepatotoxicity	No	No	No	No
CYP3A4 substrate/inhibitor	Yes/Yes	Yes/Yes	Yes/Yes	Yes/Yes
P-gp substrate/inhibitor	Yes/Yes	Yes/Yes	Yes/Yes	Yes/Yes

## Data Availability

The original contributions presented in this study are included in the article/Supplementary Materials. Further inquiries can be directed to the corresponding authors.

## Supplementary Materials

The following supporting information can be downloaded at https://www.mdpi.com/article/10.3390/ph18070962/s1, Figure S1: ^1^H NMR spectrum (DMSO-d_6_) (**3a**); Figure S2: ^13^C NMR spectrum (DMSO-d_6_) (**3a**); Figure S3: FT-IR spectrum (**3a**); Figure S4: Mass spectrum (**3a**); Figure S5: ^1^H NMR spectrum (DMSO-d_6_) (**3b**); Figure S6: ^1^H NMR spectrum (DMSO-d_6_) (**3b**); Figure S7: FT-IR spectrum (**3b**); Figure S8: Mass spectrum (**3b**); Figure S9: ^1^H NMR spectrum (DMSO-d_6_) (**5a**); Figure S10: ^13^C NMR spectrum (DMSO-d_6_) (**5b**); Figure S11: FT-IR spectrum (**5a**); Figure S12: Mass spectrum (**5a**); Figure S13: ^1^H NMR spectrum (DMSO-d_6_) (**5b**); Figure S14: ^13^C NMR spectrum (DMSO-d_6_) (**5b**); Figure S15: FT-IR spectrum (**5b**); Figure S16: Mass spectrum (**5b**); Figure S17: ^1^H NMR spectrum (CDCl_3_) (**6a**); Figure S18: ^13^C NMR spectrum (CDCl_3_) (**6a**); Figure S19: FT-IR spectrum (**6a**); Figure S20: Mass spectrum (**6a**); Figure S21: ^1^H NMR spectrum (CDCl_3_) (**6b**); Figure S22: ^13^C NMR spectrum (CDCl_3_) (**6b**); Figure S23: FT-IR spectrum (**6b**); Figure S24: Mass spectrum (**6b**); Figure S25: ^1^H NMR spectrum (CDCl_3_) (**8a**); Figure S26: ^13^C NMR spectrum (CDCl_3_) (**8a**); Figure S27: FT-IR spectrum (**8a**); Figure S28: Mass spectrum (**8a**); Figure S29: ^1^H NMR spectrum (CDCl_3_) (**8b**); Figure S30: ^13^C NMR spectrum (CDCl_3_) (**8b**); Figure S31: FT-IR spectrum (**8b**); Figure S32: Mass spectrum (**8b**); Figure S33: ^1^H NMR spectrum (CDCl_3_) (**8c**); Figure S34: ^13^C NMR spectrum (CDCl_3_) (**8c**); Figure S35: FT-IR spectrum (**8c**); Figure S36: Mass spectrum (**8c**); Figure S37: ^1^H NMR spectrum (CDCl_3_) (**8d**); Figure S38: ^13^C NMR spectrum (CDCl_3_) (**8d**); Figure S39: FT-IR spectrum (**8d**); Figure S40: Mass spectrum (**8d**); Figure S41: Pairwise sequence alignment of human acetylcholinesterase (hAChE, UniProt ID: P22303) and Electrophorus electricus acetylcholinesterase (eAChE, UniProt ID: O42275); Figure S42: Pairwise sequence alignment of human butyrylcholinesterase (hBChE, UniProt ID: P06276) and equine butyrylcholinesterase (eBChE, UniProt ID: P81908); Figure S44: Redocking validation of acetazolamide in the active site of hCA II (PDB: 3HS4); Figure S43: Redocking validation of acetazolamide in the active site of hCA I (PDB: 1AZM); Figure S45: Redocking validation of tacrine in the active site of AChE (PDB: 7XN1); Figure S46: Redocking validation of tacrine in the active site of BChE (PDB: 4BDS).
