# Supplementary material for: Synthesis, Biological Evaluation, and In Silico Characterization of Novel Imidazothiadiazole–Chalcone Hybrids as Multi-Target Enzyme Inhibitors
Source: Pharmaceuticals (Basel). 2025 Jun 26;18(7):962. doi: 10.3390/ph18070962 (PMC12300381; doi:10.3390/ph18070962)
Supplement: Supplementary file 1 [file pharmaceuticals-18-00962-s001.zip › pharmaceuticals-3693438-supplementary-final.pdf]

Supplementary Materials

Synthesis, Biological Evaluation and In Silico Characterization of Novel Imidazothiadiazole-Chalcone Hybrids as Multi-Target Enzyme Inhibitors

Hakan Alici [1,*], Senol Topuz [1], Kadir Demir [1], Parham Taslimi [2], and Hakan Tahtaci [3,*]

1 Department of Physics, Faculty of Science, Zonguldak Bülent Ecevit University, 67100 Zonguldak, Türkiye; hakanalici@beun.edu.tr (H.A.); senol.topuz@fbe.karaelmas.edu.tr (S.T.); kadirdemir@beun.edu.tr (K.D.)
2 Bartin University, Department of Biotechnology, Faculty of Science, 74110, Bartın, Türkiye; ptaslimi@bartin.edu.tr (P.T)
3 Karabuk University, Department of Chemistry, Faculty of Science, 78050, Karabuk, Türkiye; hakantahtaci@karabuk.edu.tr (H.T)
* Correspondence: hakanalici@beun.edu.tr (H.A); hakantahtaci@karabuk.edu.tr (H.T.)

Table of Contents

^{1}H NMR, ^{13}C NMR, FT-IR, and Mass spectra copies of all compounds ……………………………………………**S2-S21**
Pairwise sequence alignment results ..…………………………………………………………………………**S22-S23**
Redocking validation of target enzymes..………………………………...……………………………...…**S24-S25**

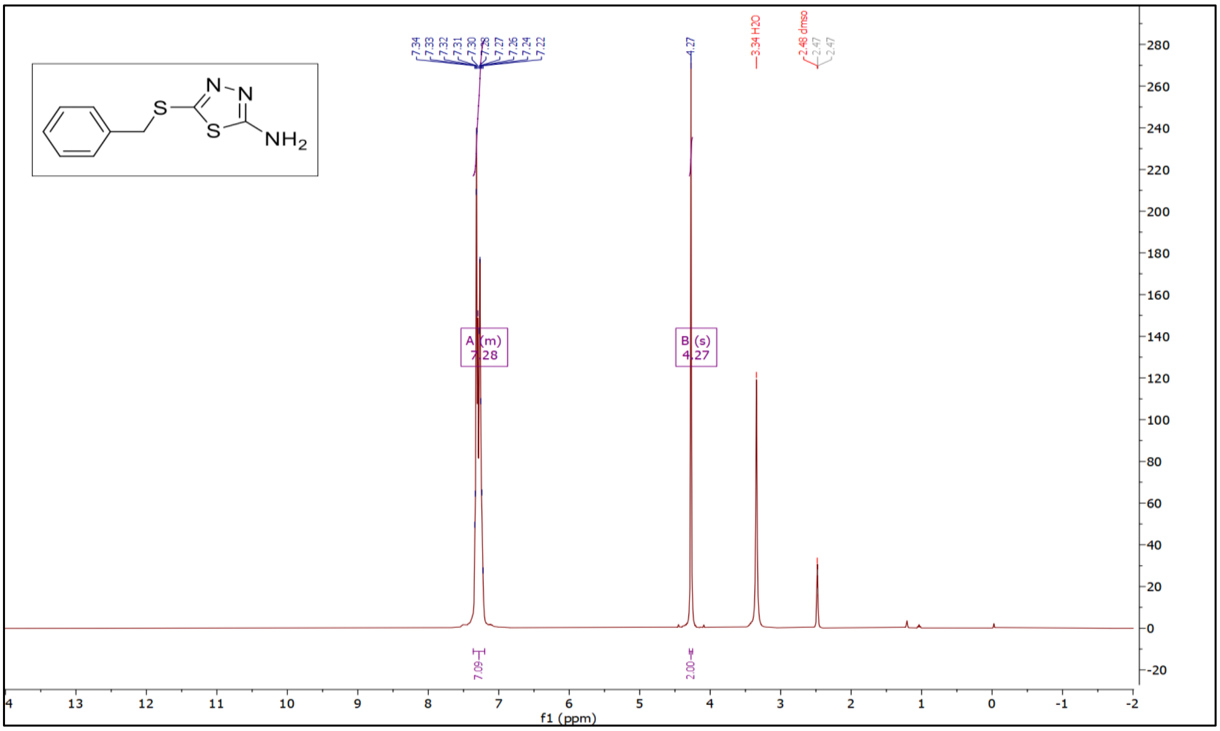

Figure S1. ^{1}H NMR Spectrum (DMSO-d$_6$) **(3a).**

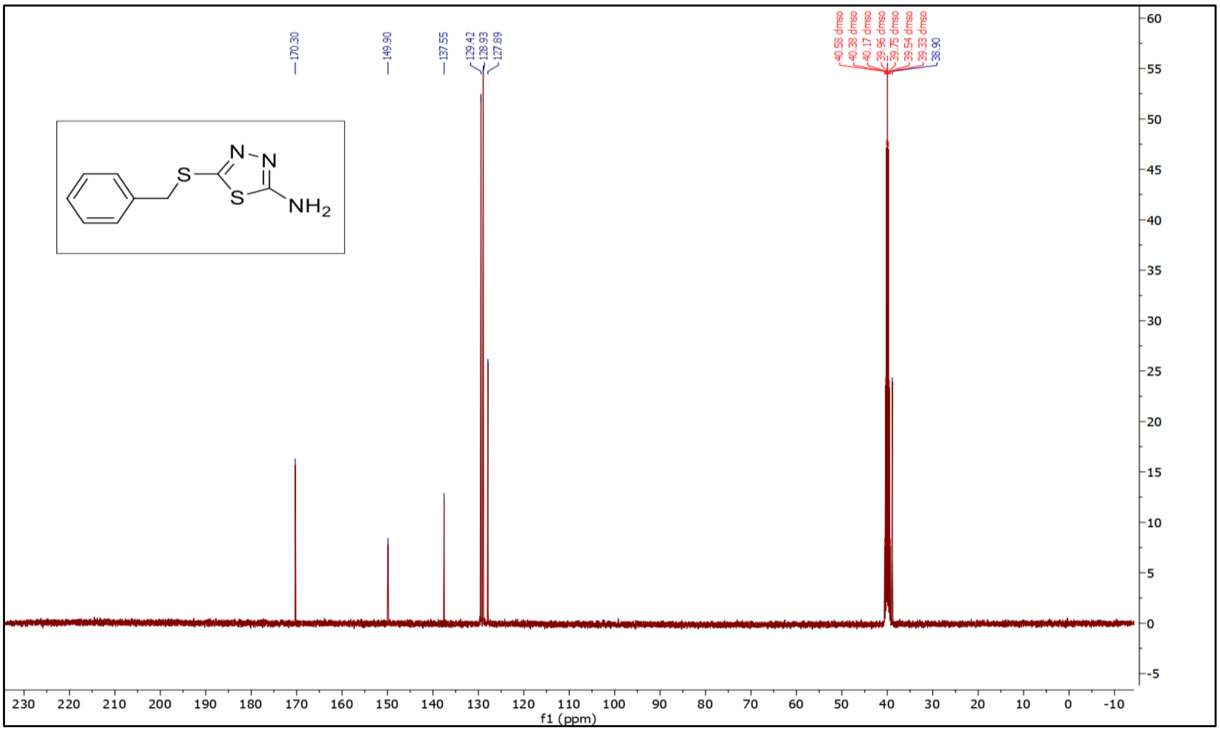

Figure S2. ^{13}C NMR Spectrum (DMSO-d$_6$) **(3a).**

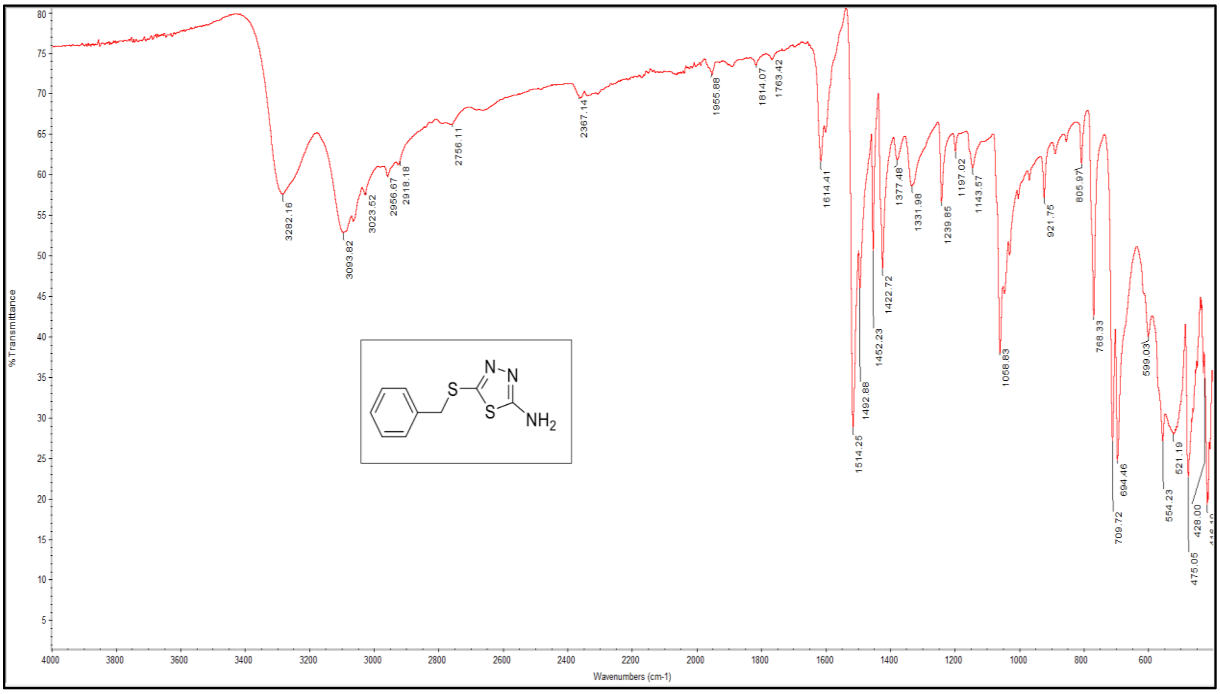

Figure S3. FT-IR Spectrum **(3a).**

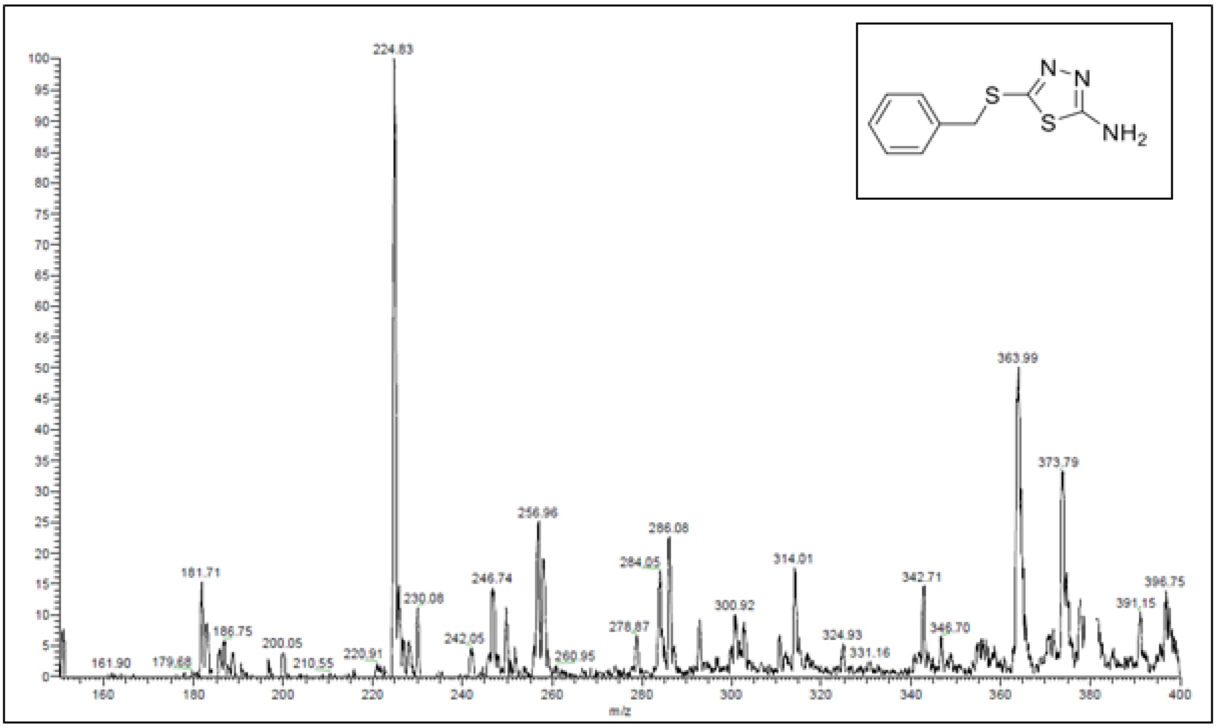

Figure S4. Mass Spectrum **(3a).**

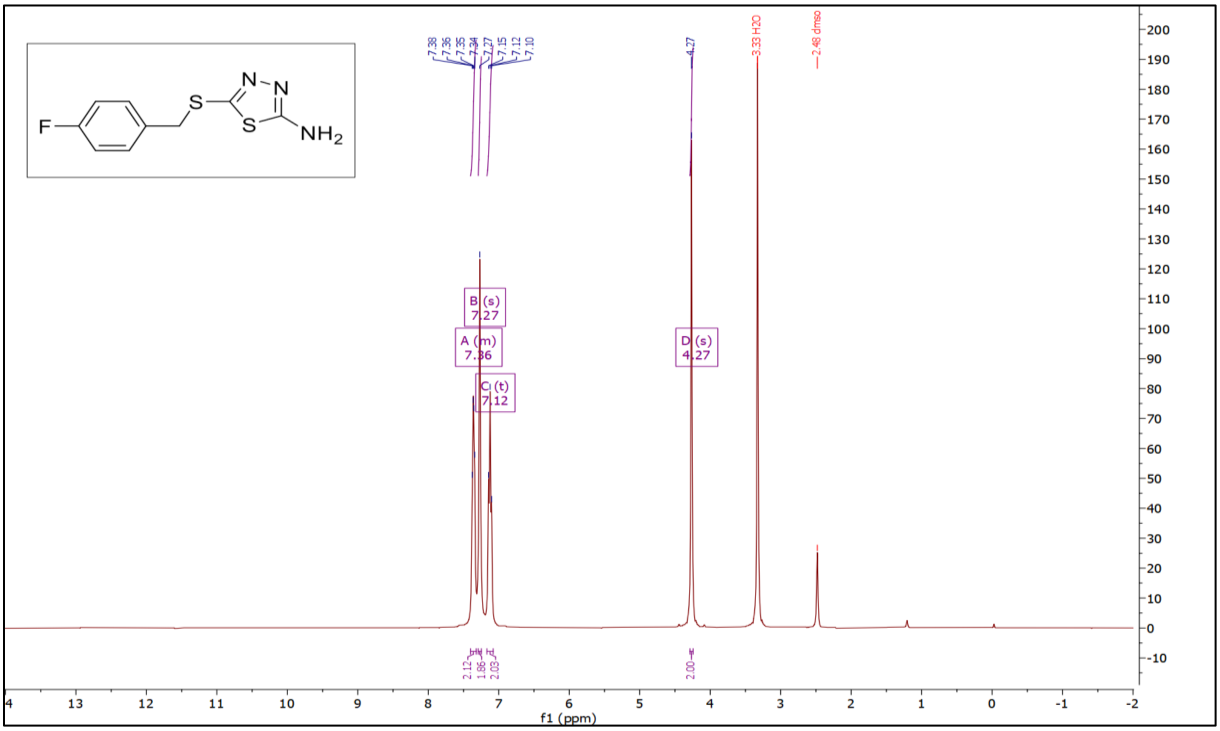

Figure S5. ^{1}H NMR Spectrum (DMSO-d$_6$) **(3b).**

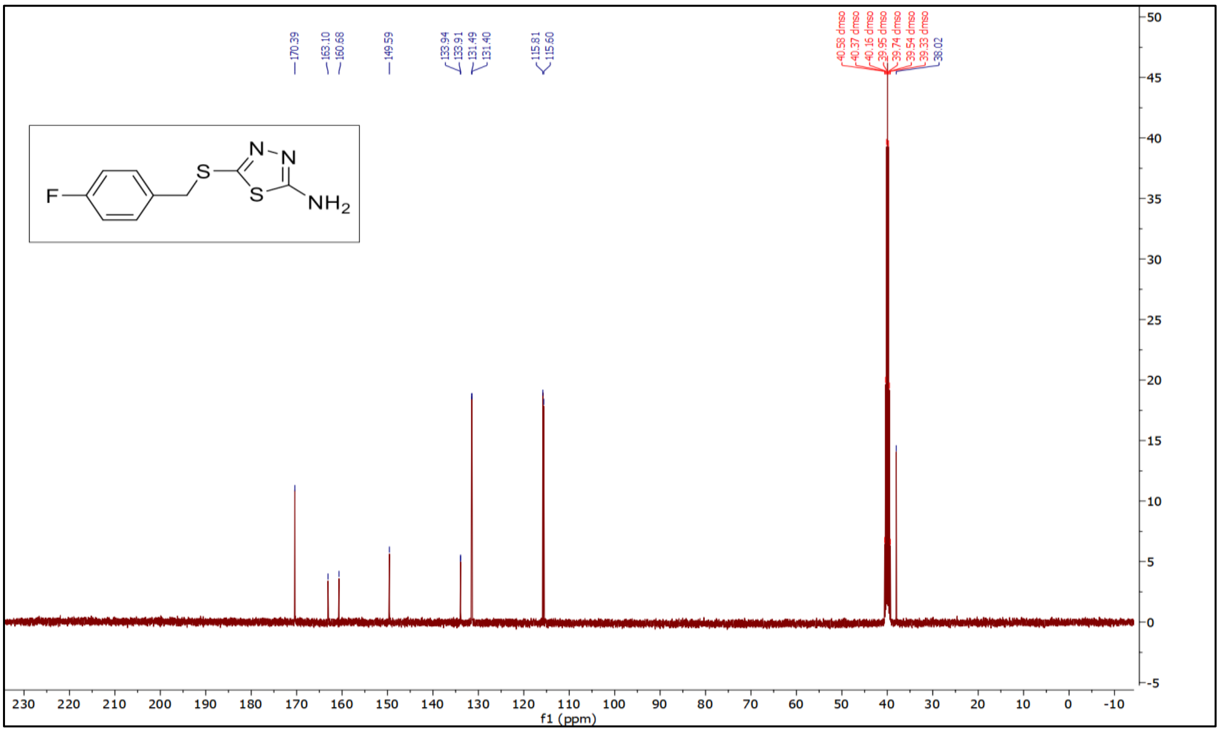

Figure S6. ^{1}H NMR Spectrum (DMSO-d$_6$) **(3b).**

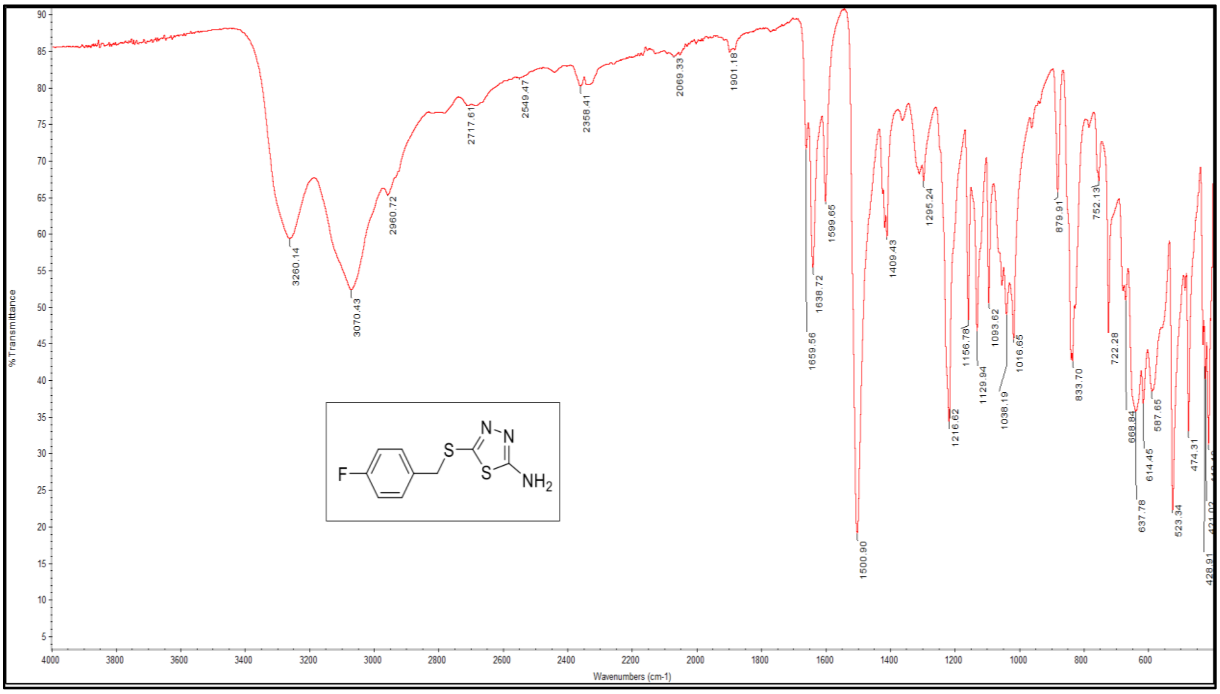

Figure S7. FT-IR Spectrum **(3b).**

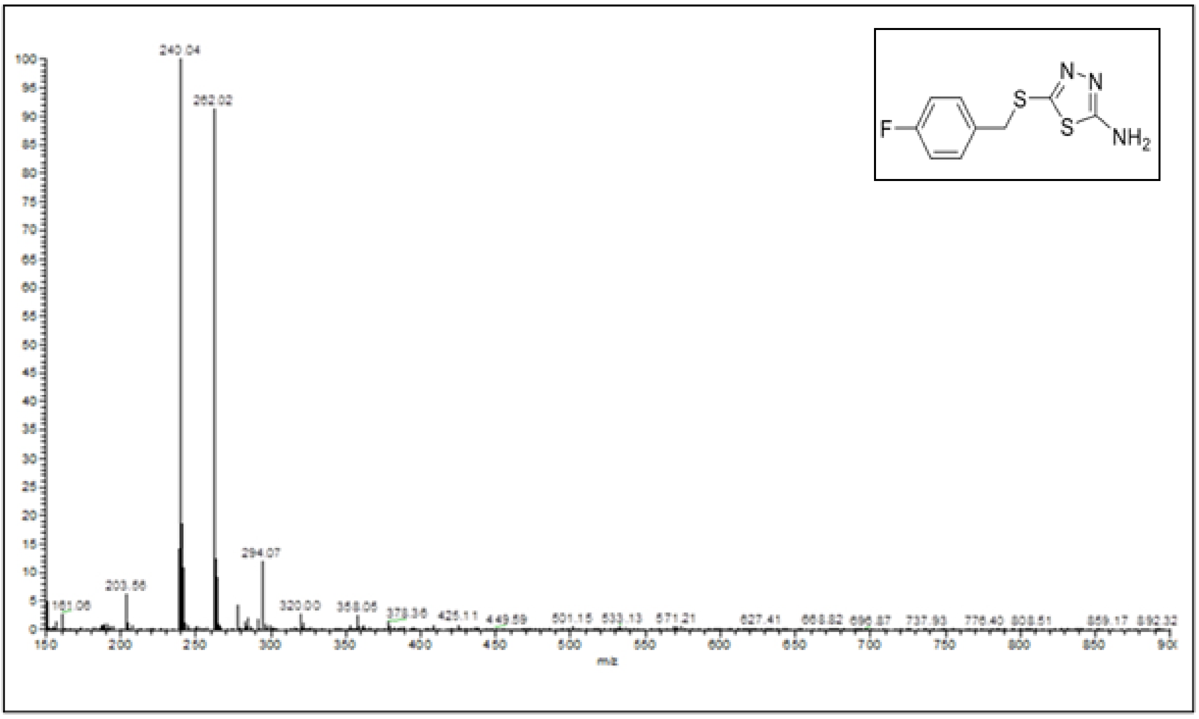

Figure S8. Mass Spectrum **(3b).**

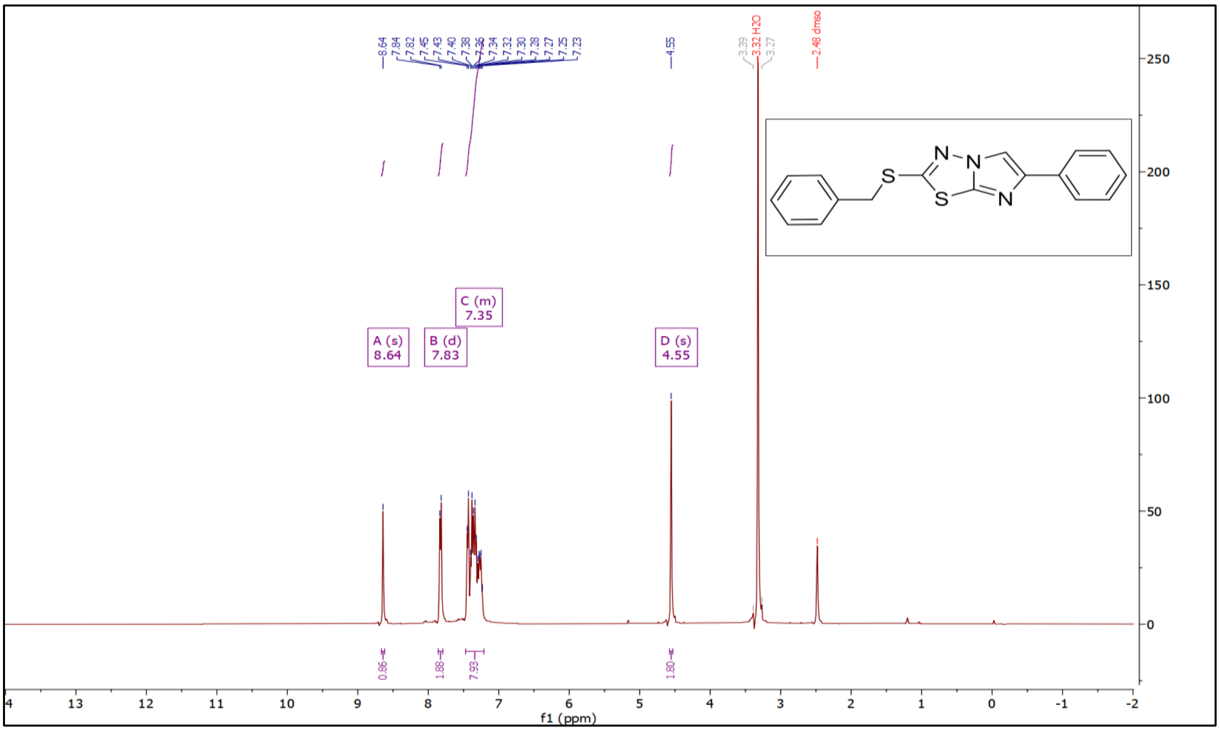

Figure S9. ^{1}H NMR Spectrum (DMSO-d$_6$) **(5a).**

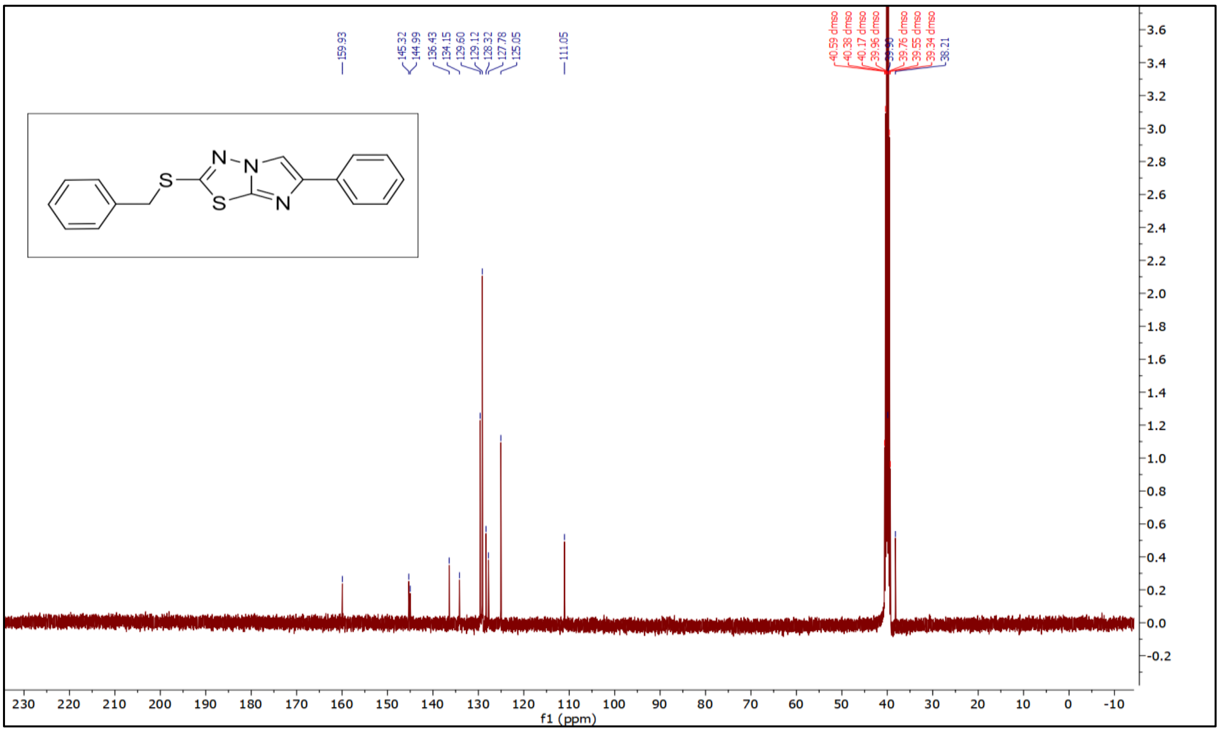

Figure S10. ^{13}C NMR Spectrum (DMSO-d$_6$) **(5b).**

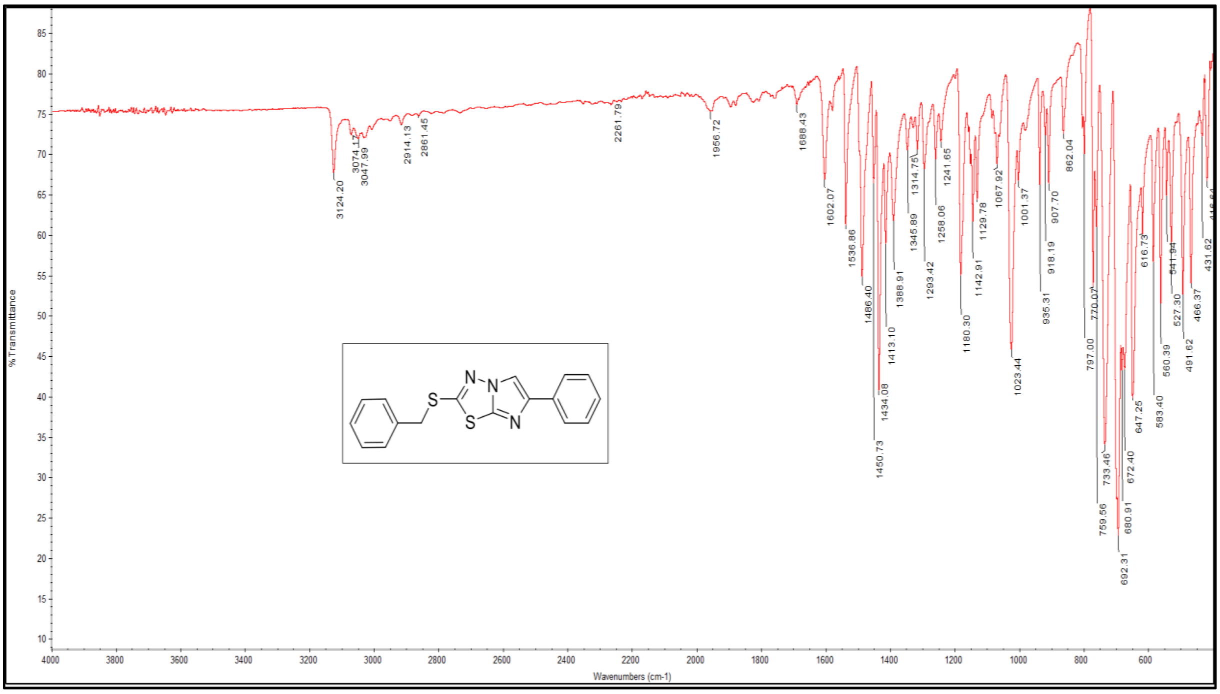

Figure S11. FT-IR Spectrum **(5a).**

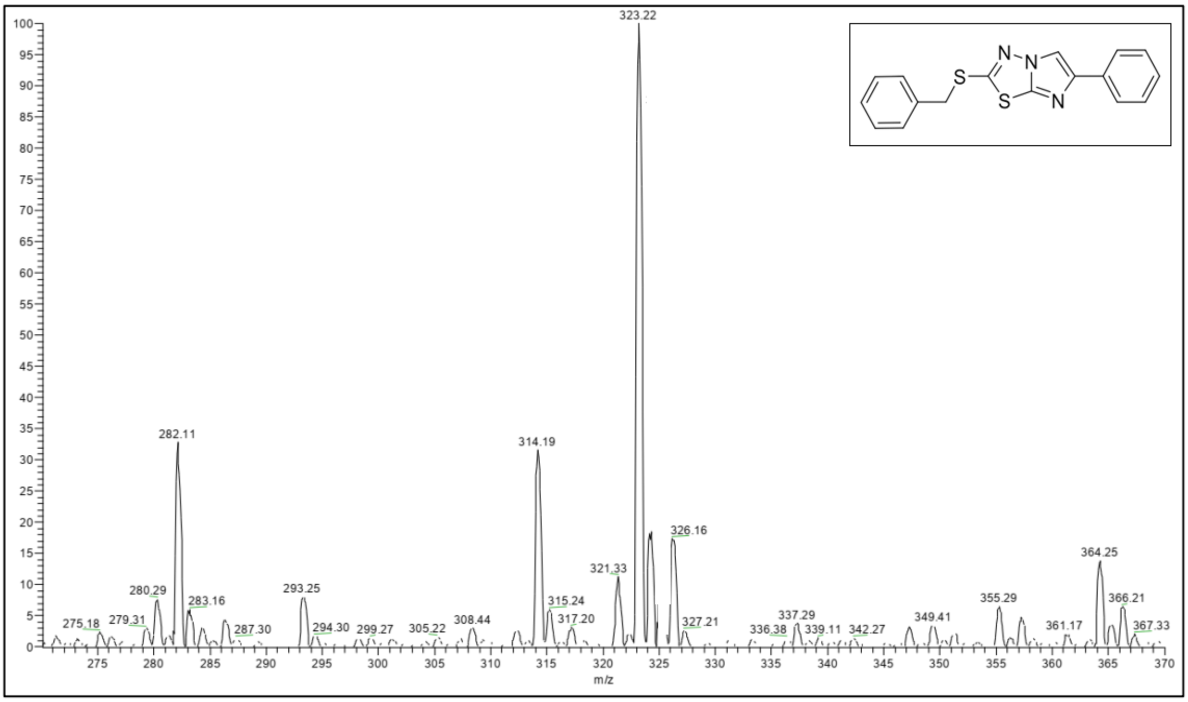

Figure S12. Mass Spectrum **(5a).**

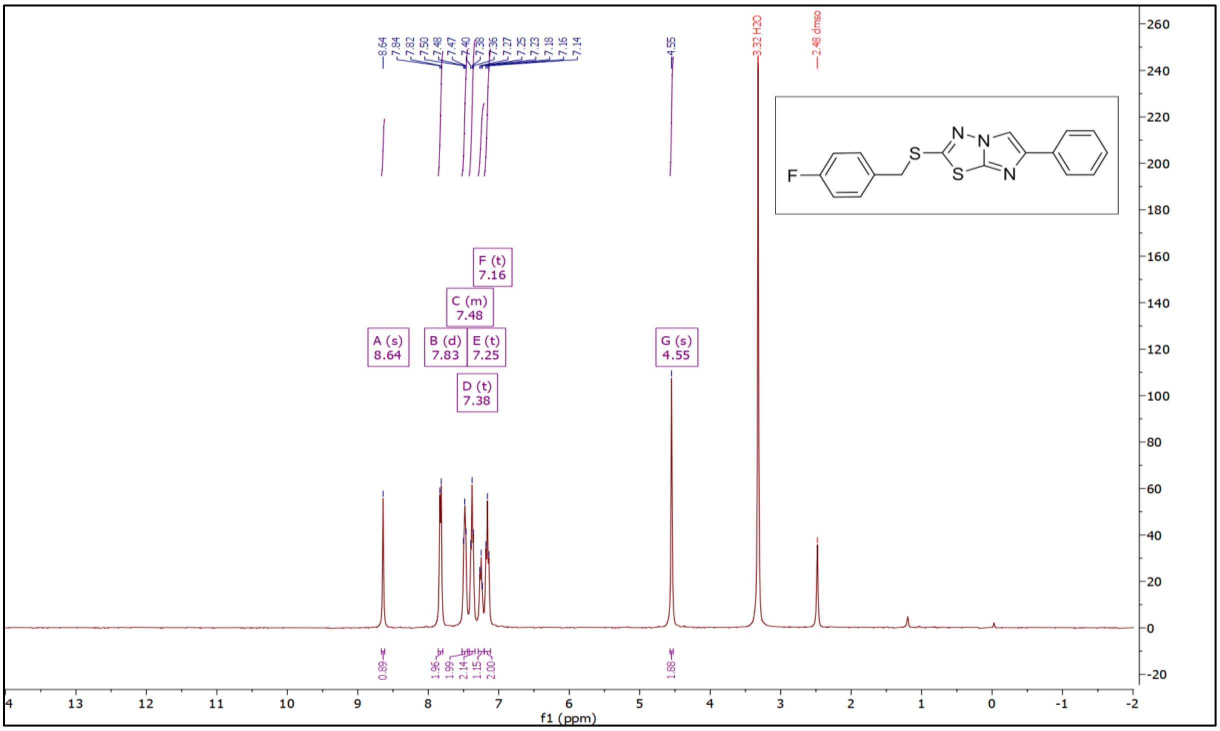

Figure S13. ^{1}H NMR Spectrum (DMSO-d_6) **(5b).**

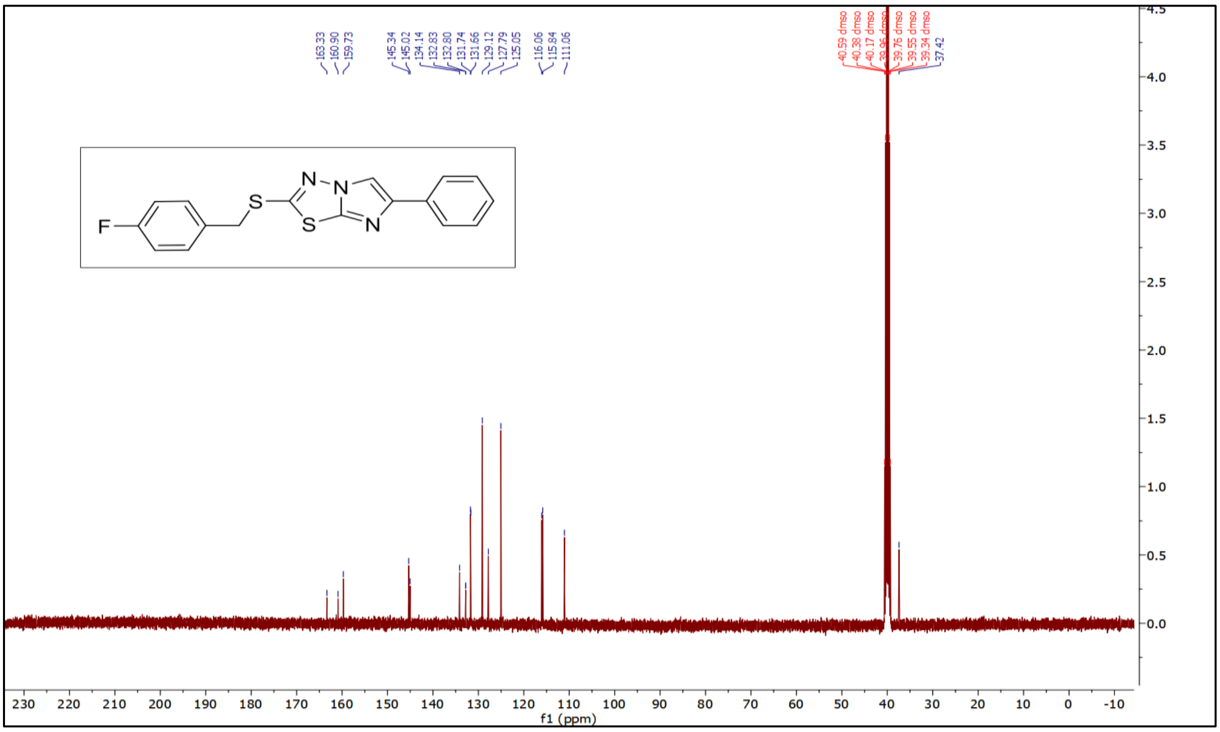

Figure S14. ^{13}C NMR Spectrum (DMSO-d_6) **(5b).**

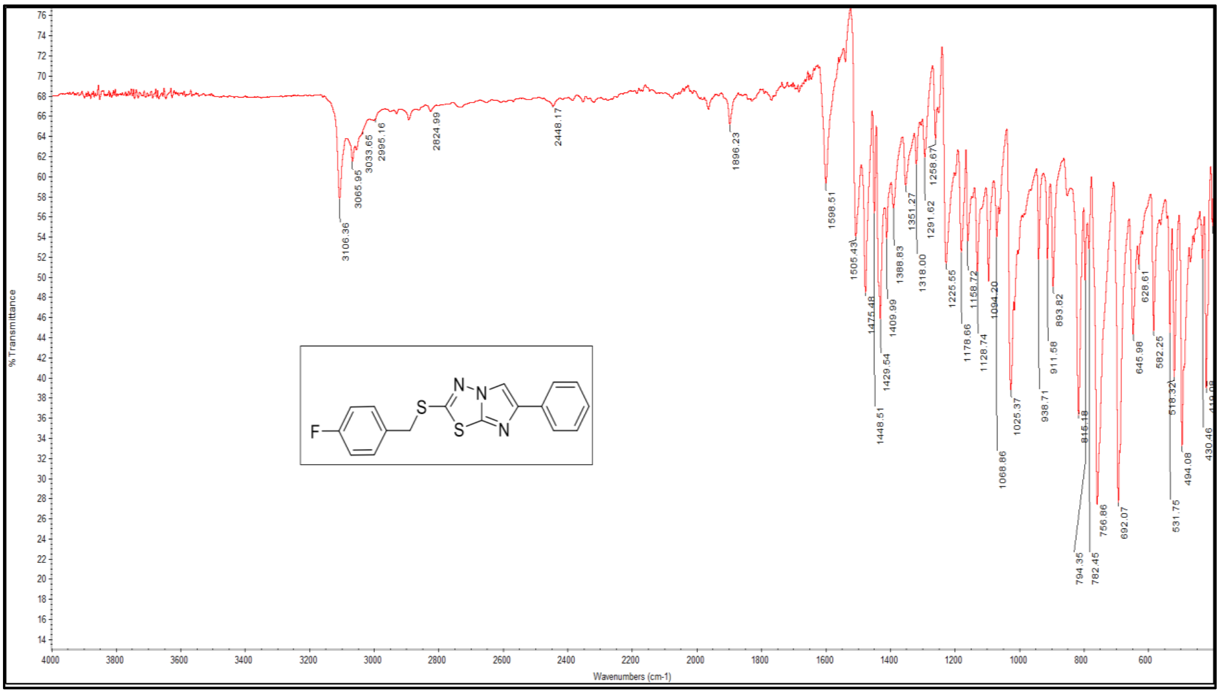

Figure S15. FT-IR Spectrum **(5b).**

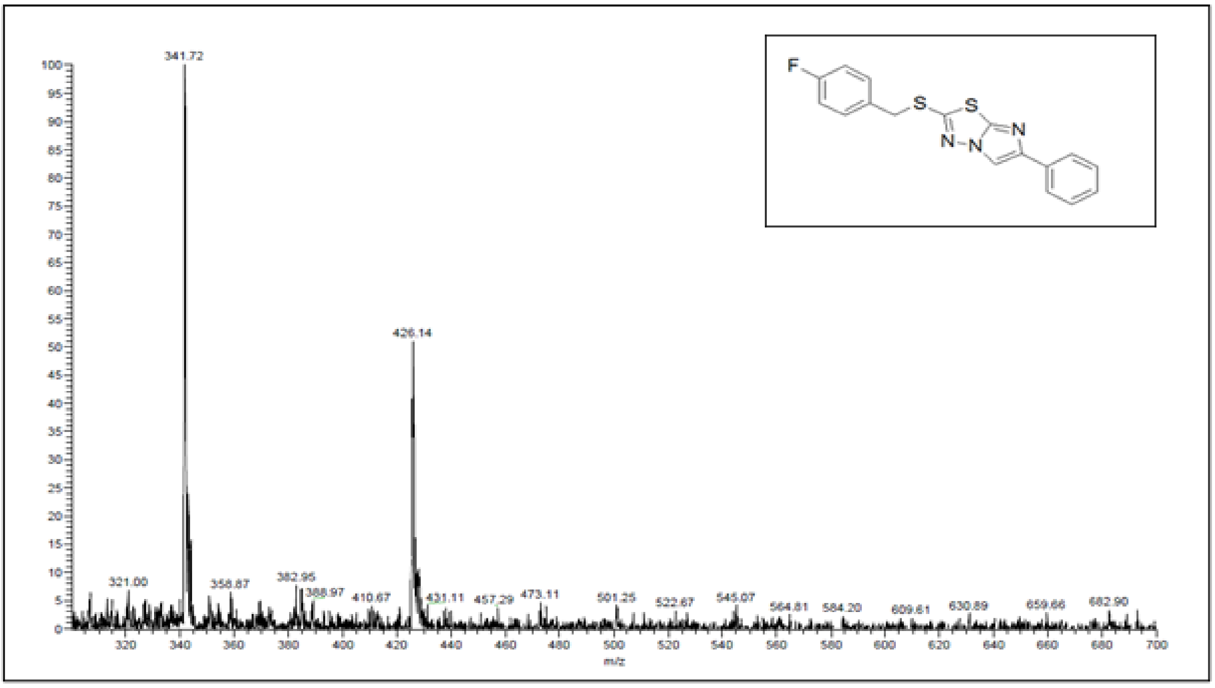

Figure S16. Mass Spectrum **(5b).**

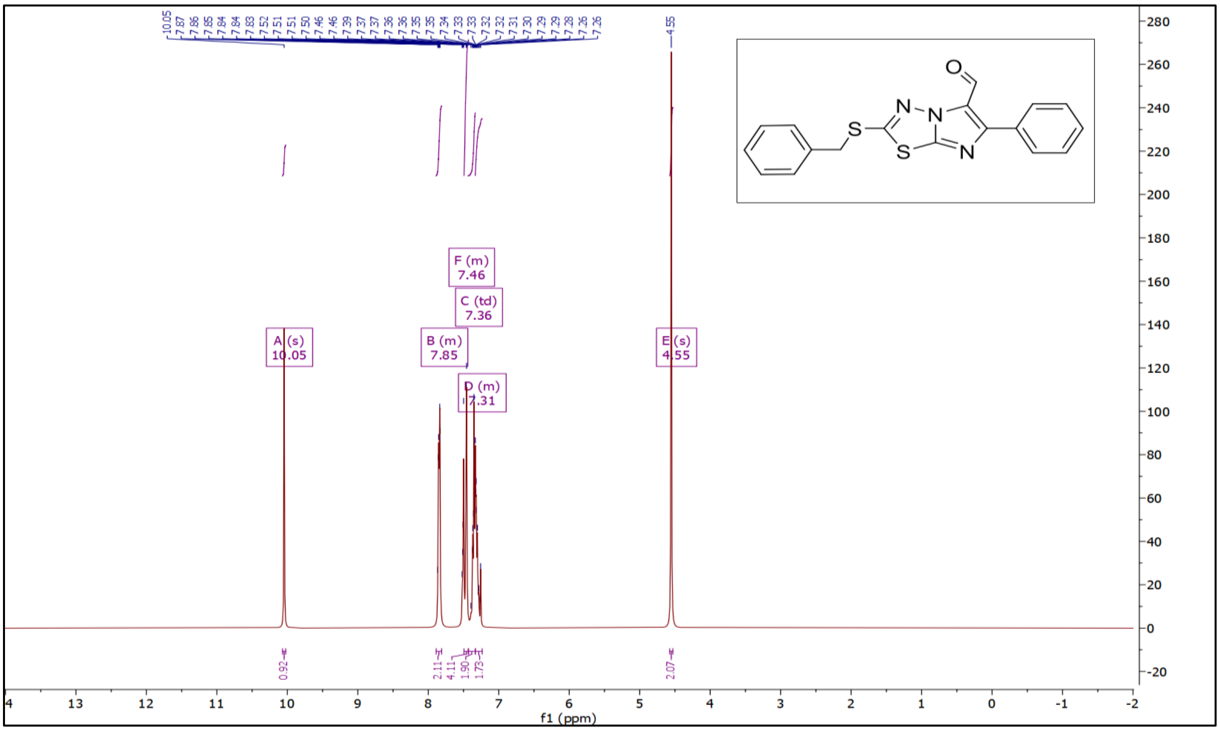

Figure S17. ^{1}H NMR Spectrum ($CDCl_3$) **(6a).**

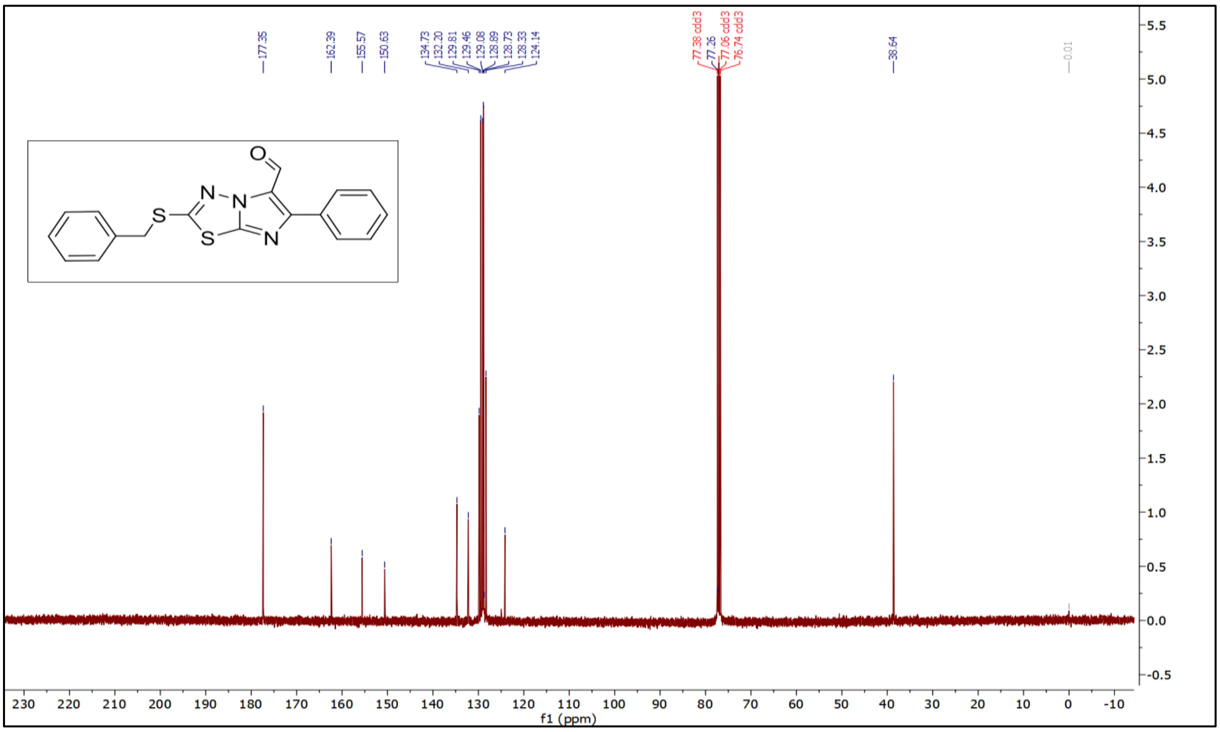

Figure S18. ^{13}C NMR Spectrum ($CDCl_3$) **(6a).**

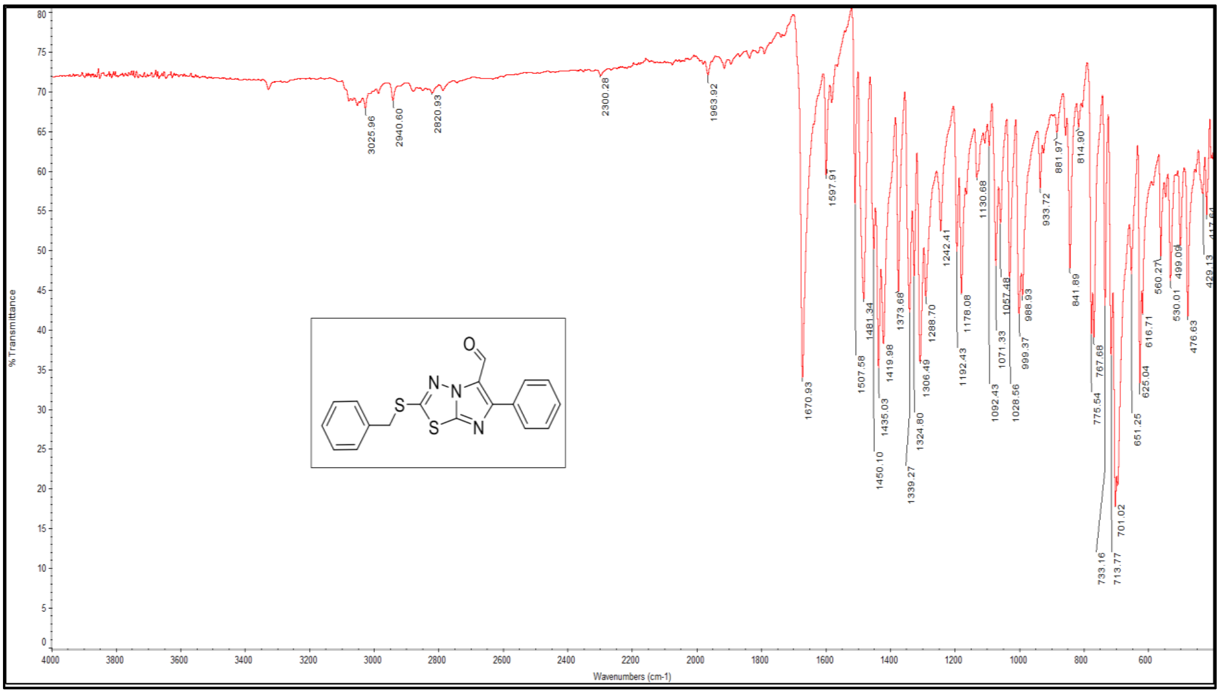

Figure S19. FT-IR Spectrum **(6a).**

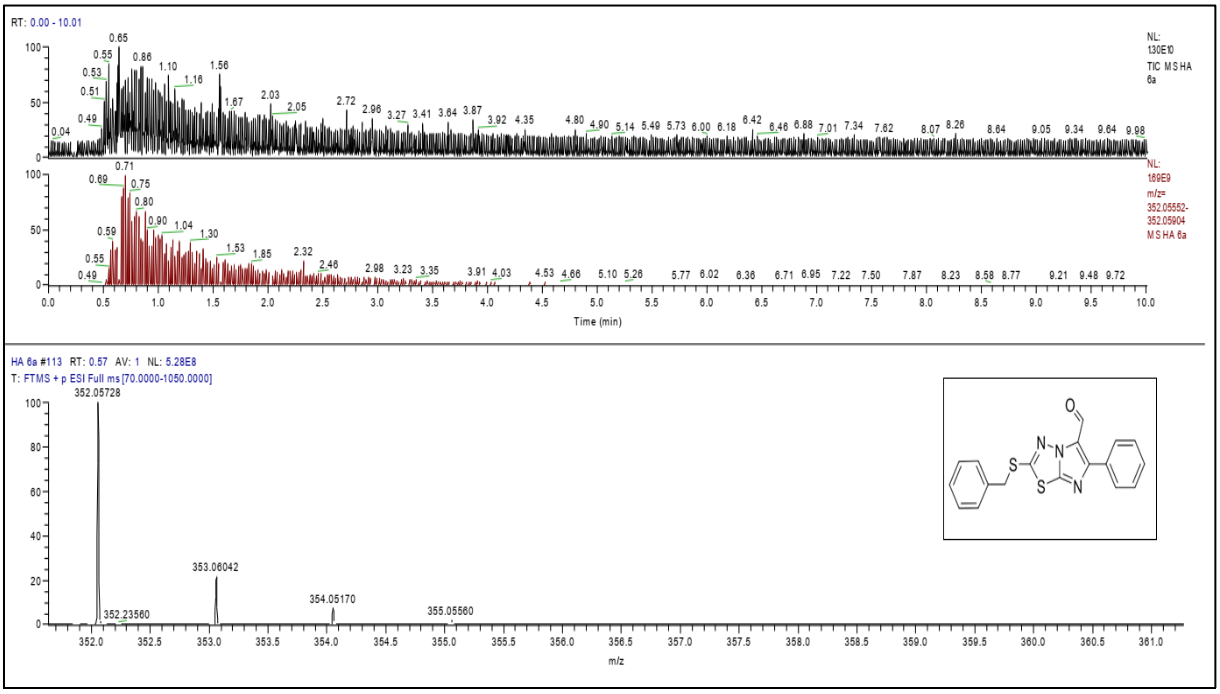

Figure S20. Mass Spectrum **(6a).**

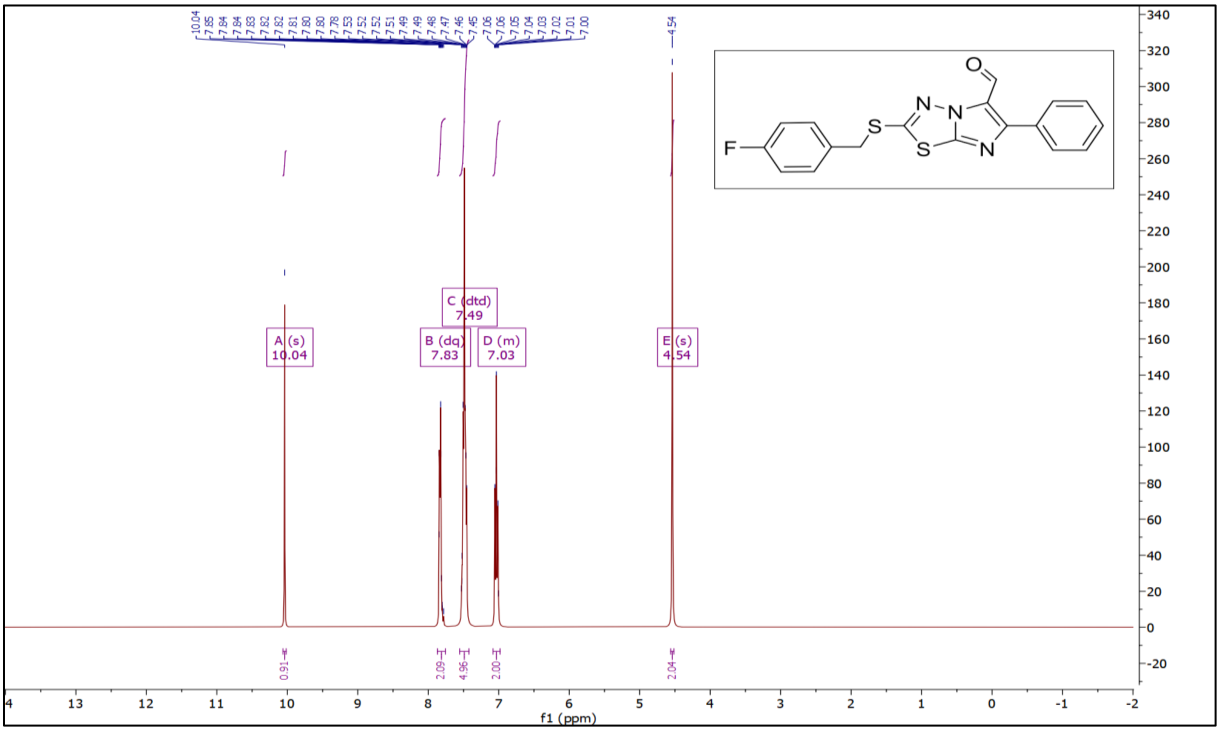

Figure S21. ^{1}H NMR Spectrum ($CDCl_3$) **(6b).**

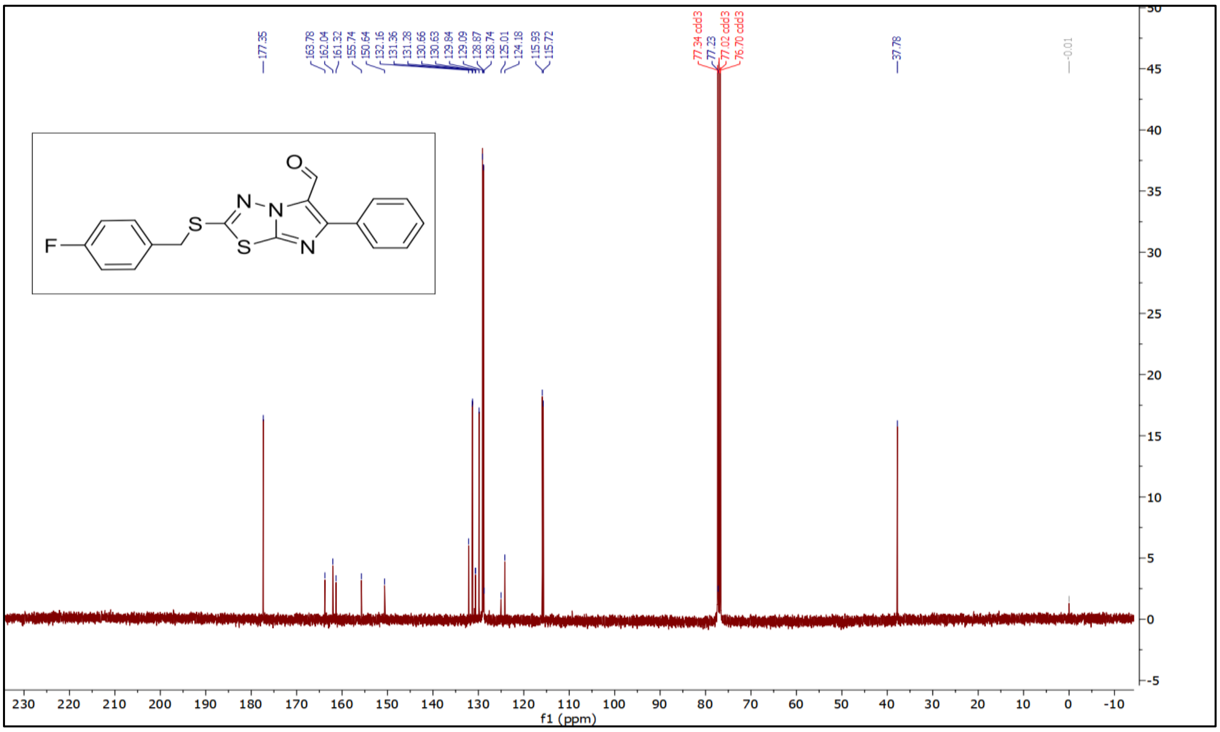

Figure S22. ^{13}C NMR Spectrum ($CDCl_3$) **(6b).**

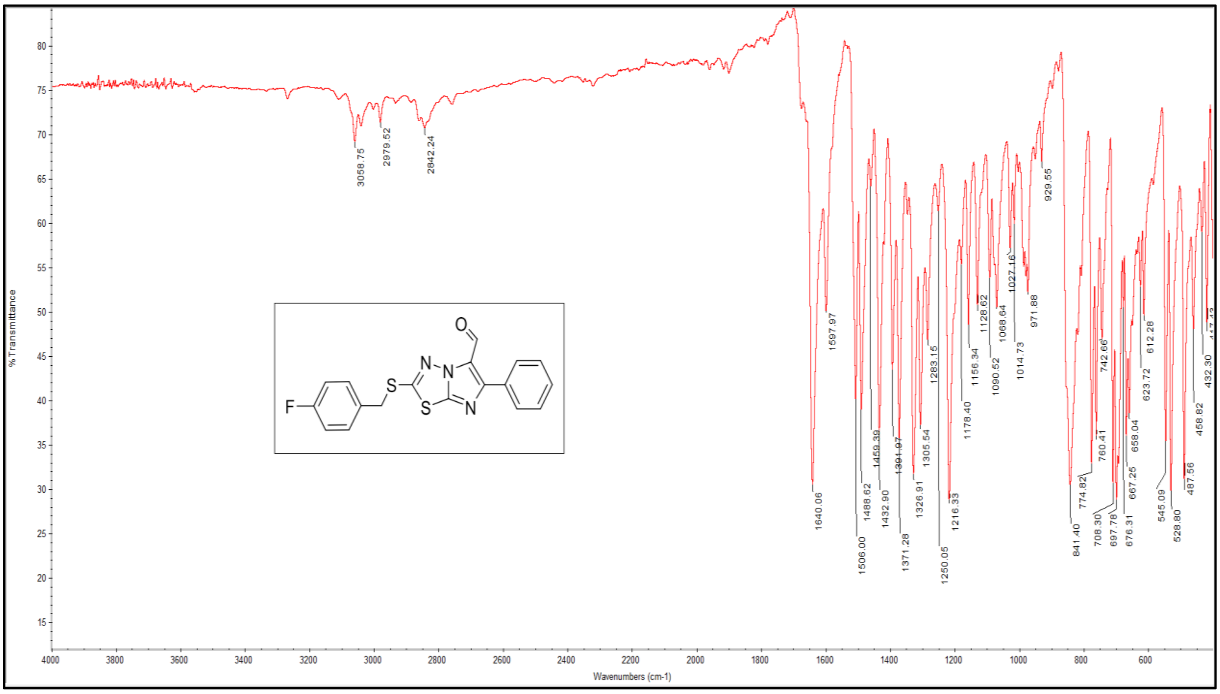

Figure S23. FT-IR Spectrum **(6b).**

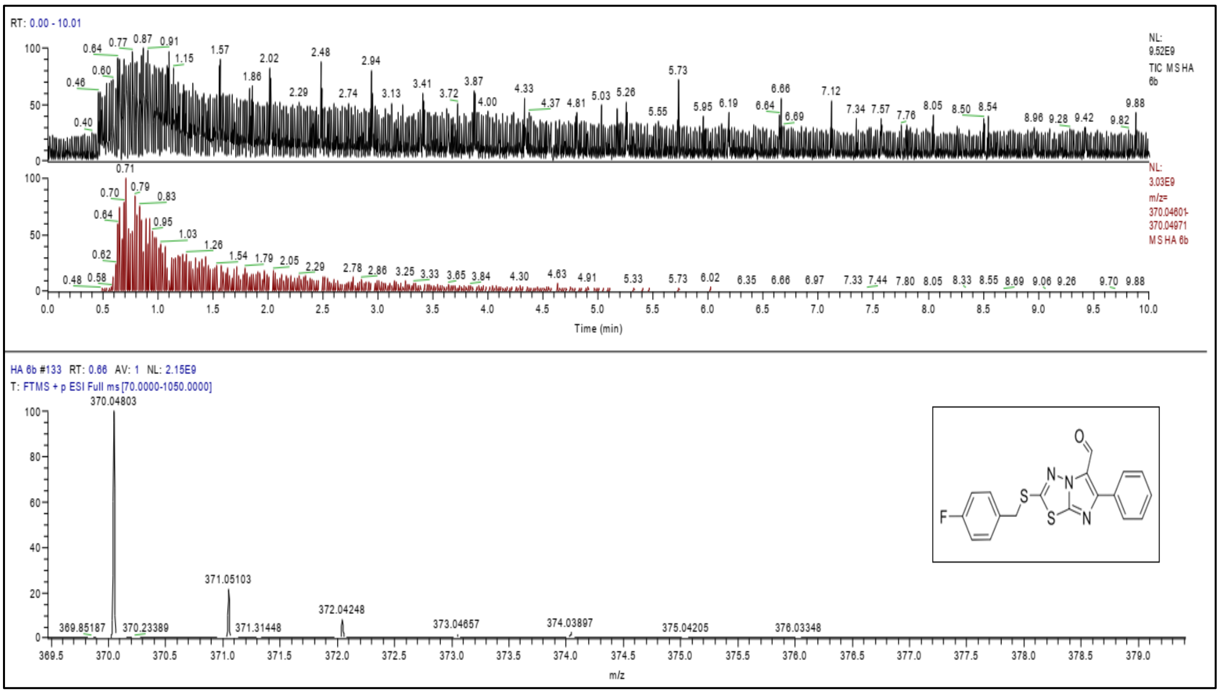

Figure S24. Mass Spectrum **(6b).**

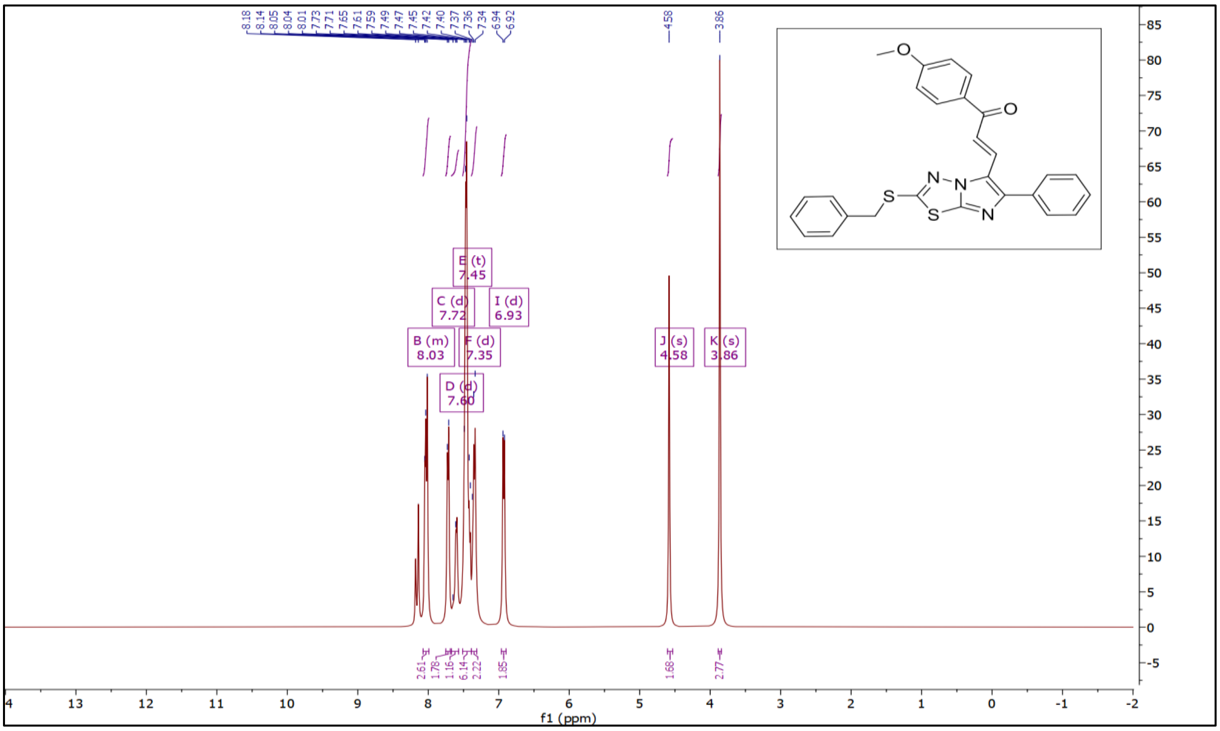

Figure S25. ^{1}H NMR Spectrum ($CDCl_3$) **(8a).**

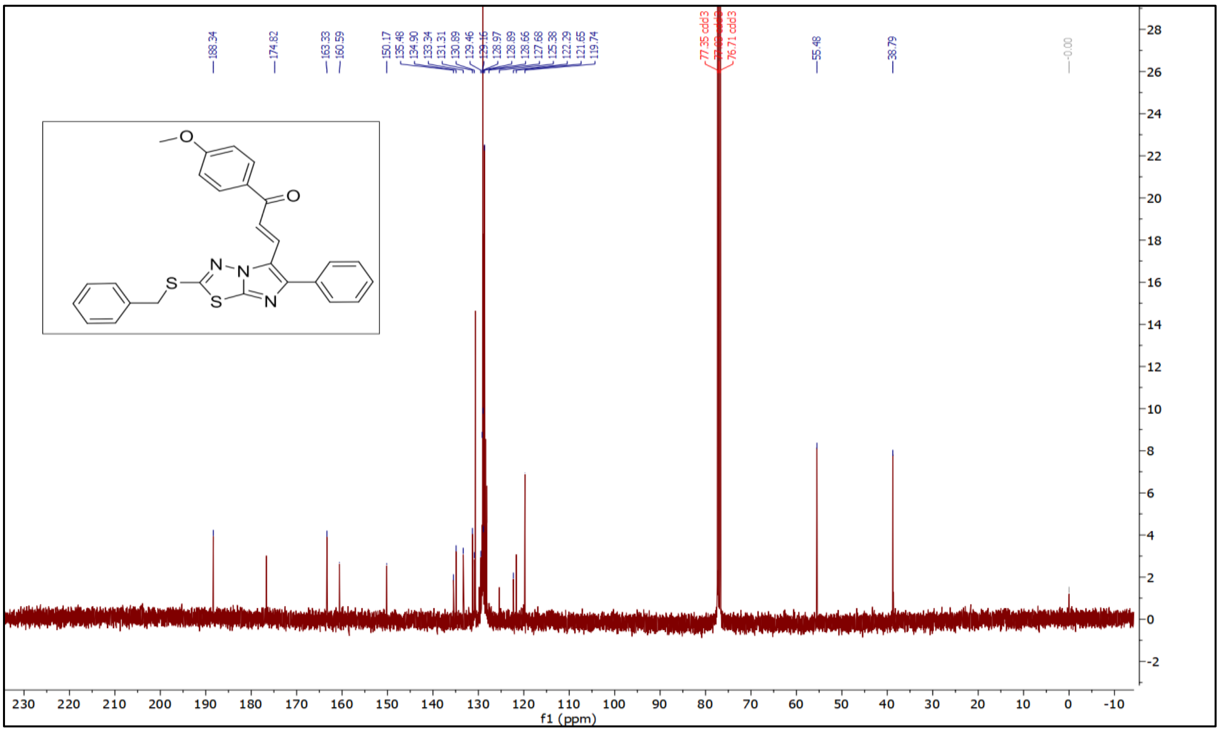

Figure S26. ^{13}C NMR Spectrum ($CDCl_3$) **(8a).**

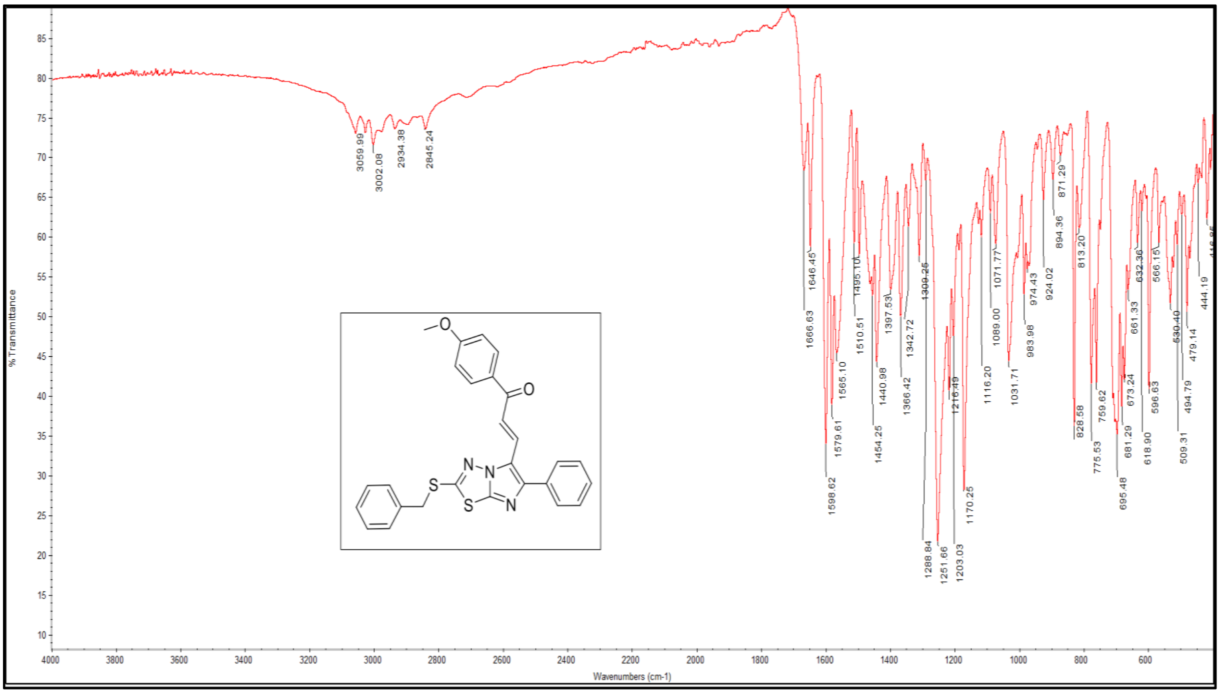

Figure S27. FT-IR Spectrum **(8a).**

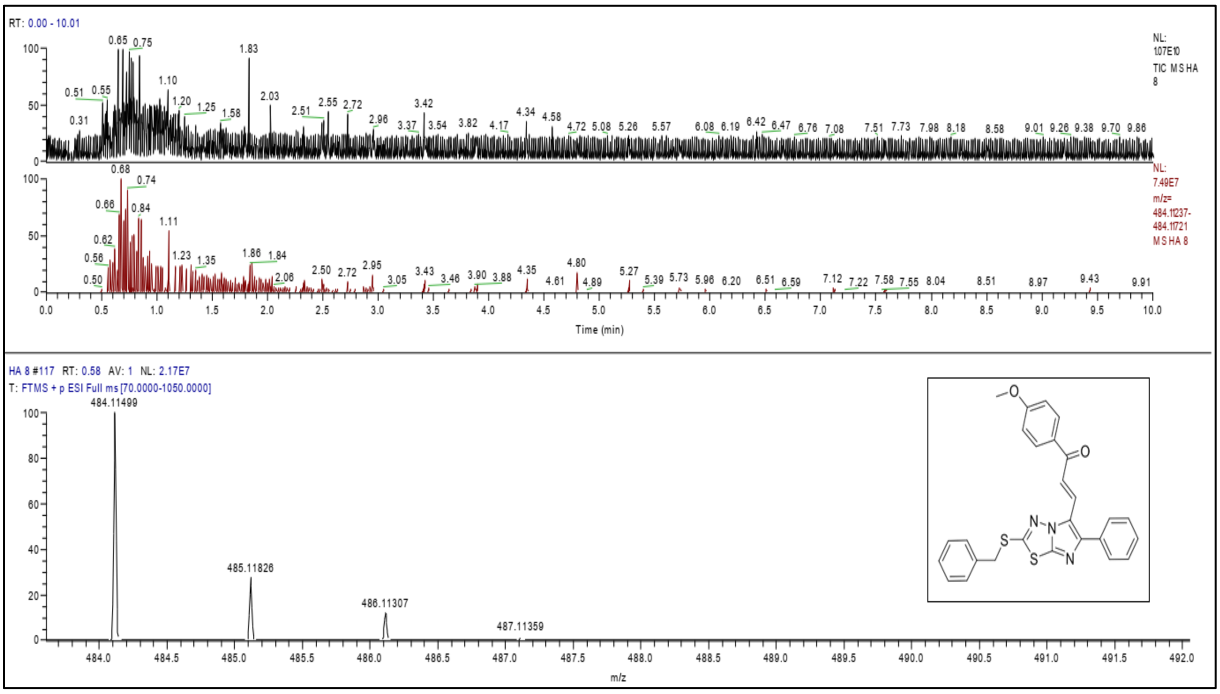

Figure S28. Mass Spectrum **(8a).**

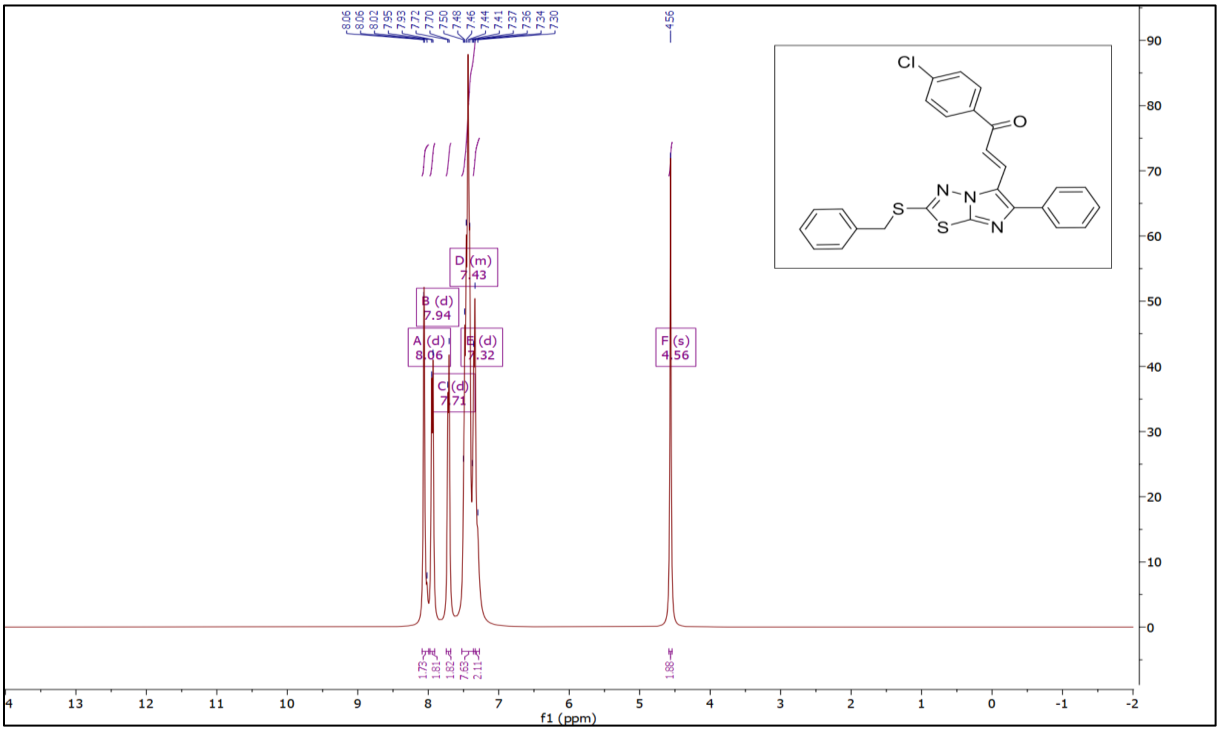

Figure S29. ^{1}H NMR Spectrum ($CDCl_3$) **(8b).**

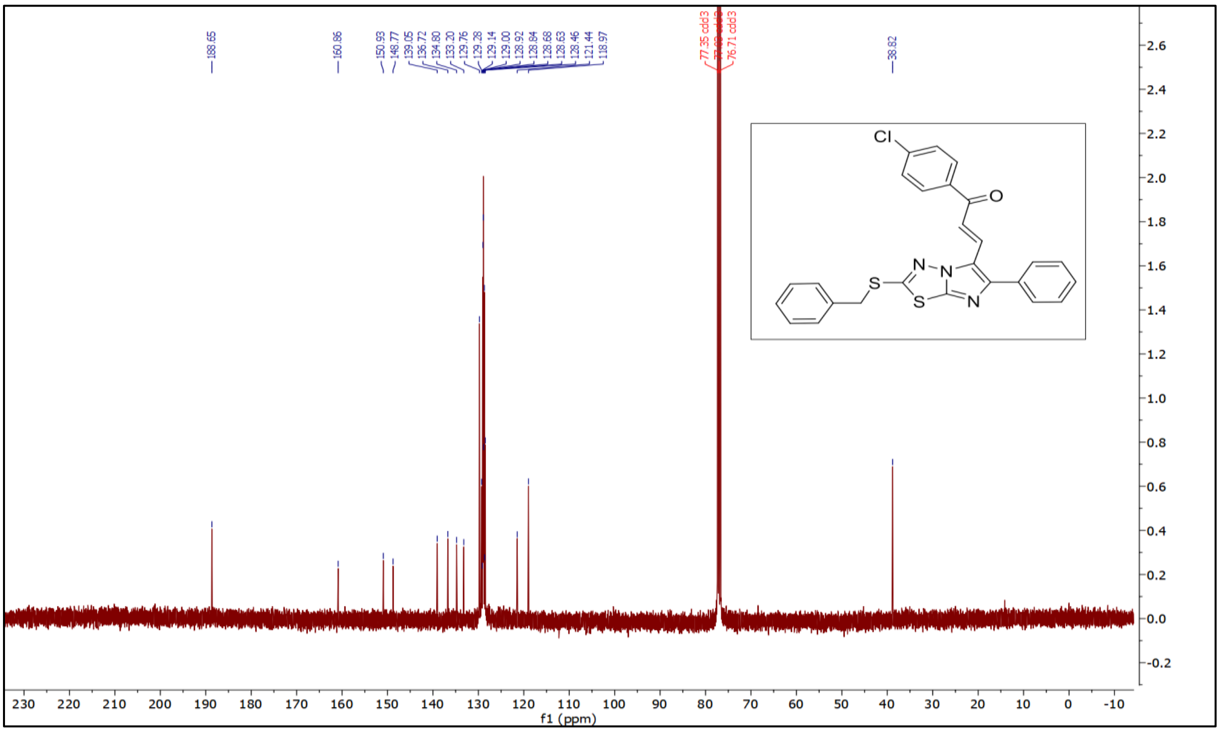

Figure S30. ^{13}C NMR Spectrum ($CDCl_3$) **(8b).**

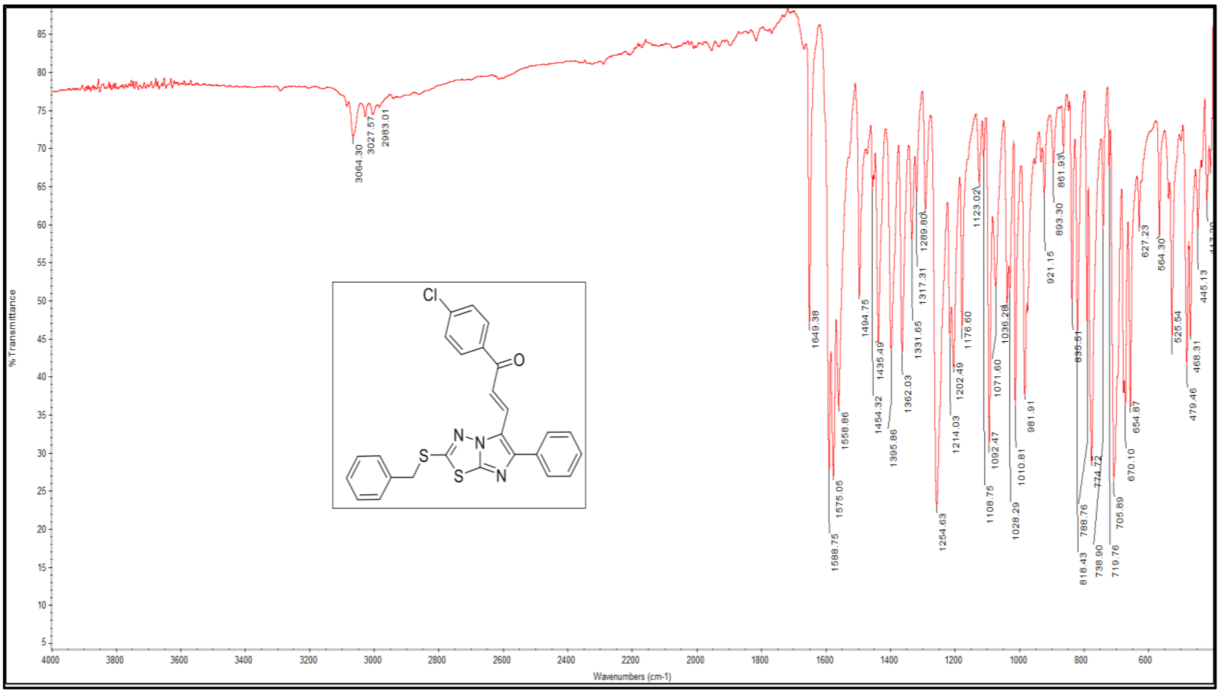

Figure S31. FT-IR Spectrum **(8b).**

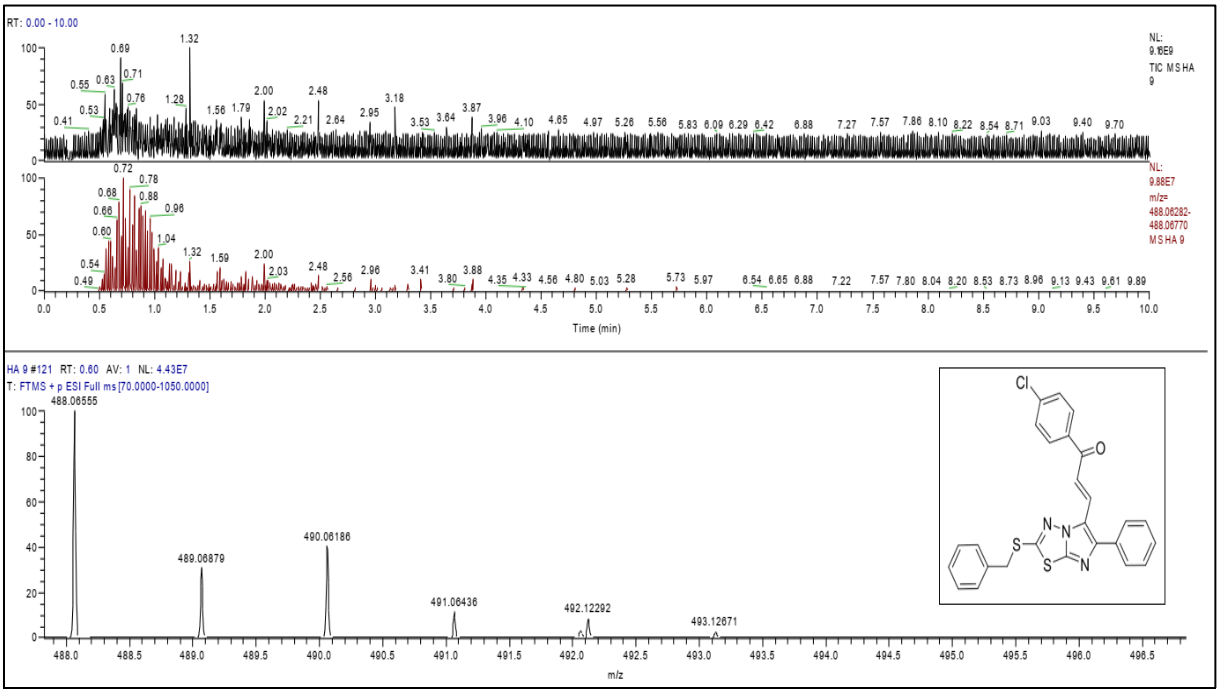

Figure S32. Mass Spectrum **(8b).**

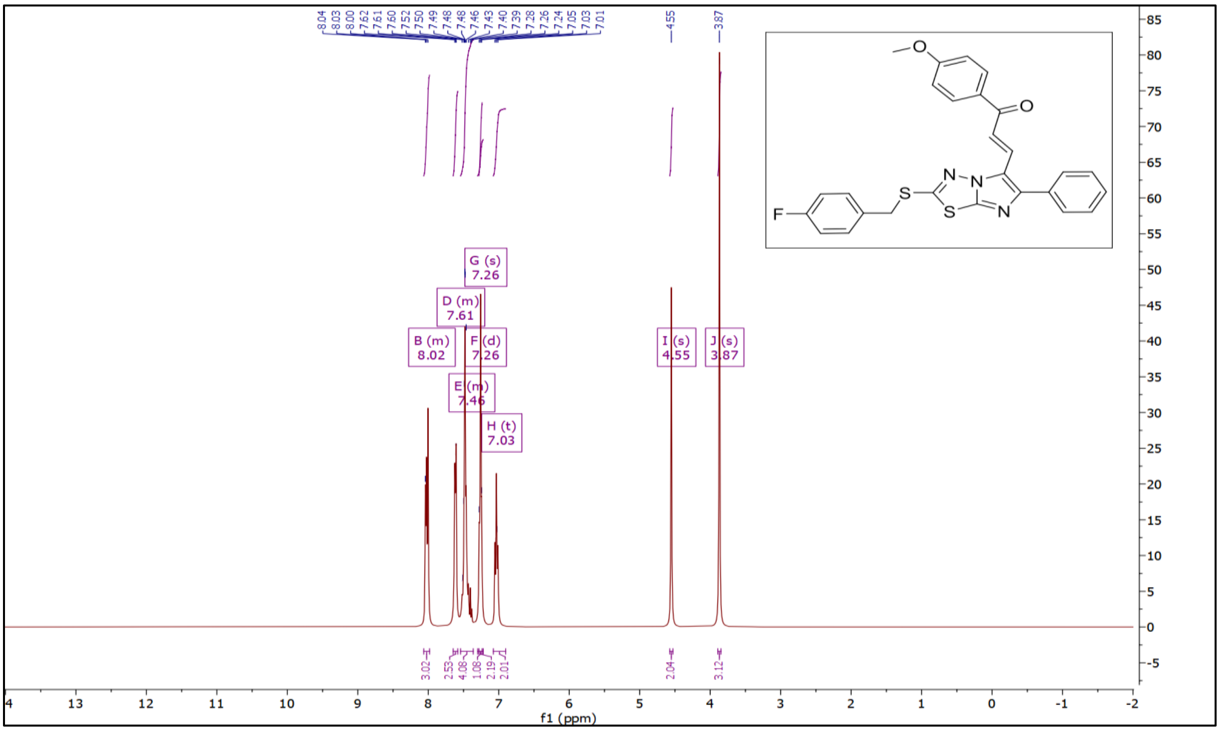

Figure S33. ^{1}H NMR Spectrum ($CDCl_3$) **(8c).**

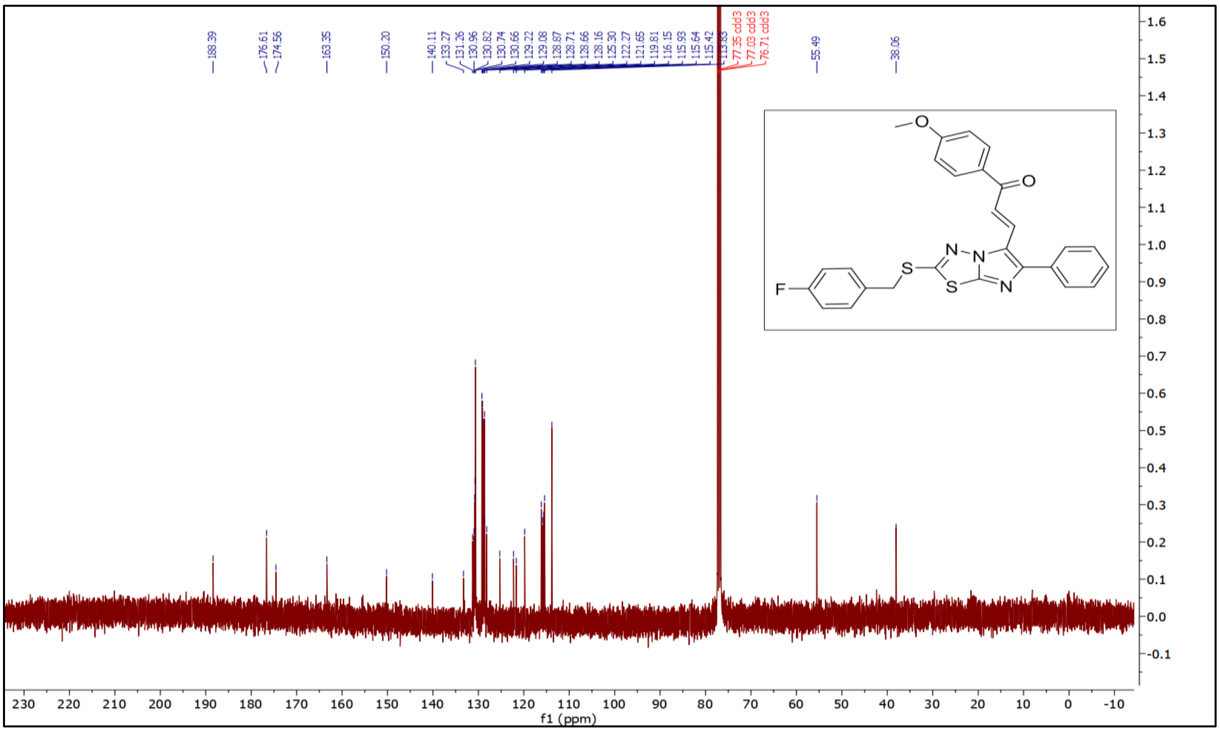

Figure S34. ^{13}C NMR Spectrum ($CDCl_3$) **(8c).**

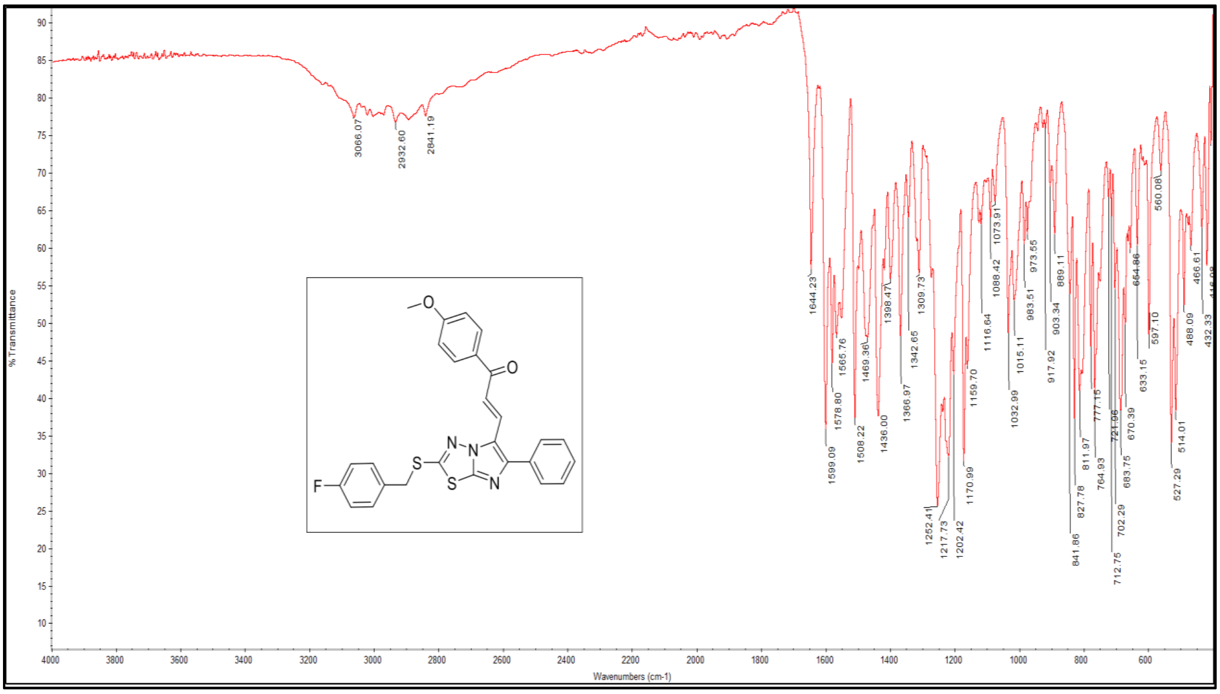

Figure S35. FT-IR Spectrum **(8c).**

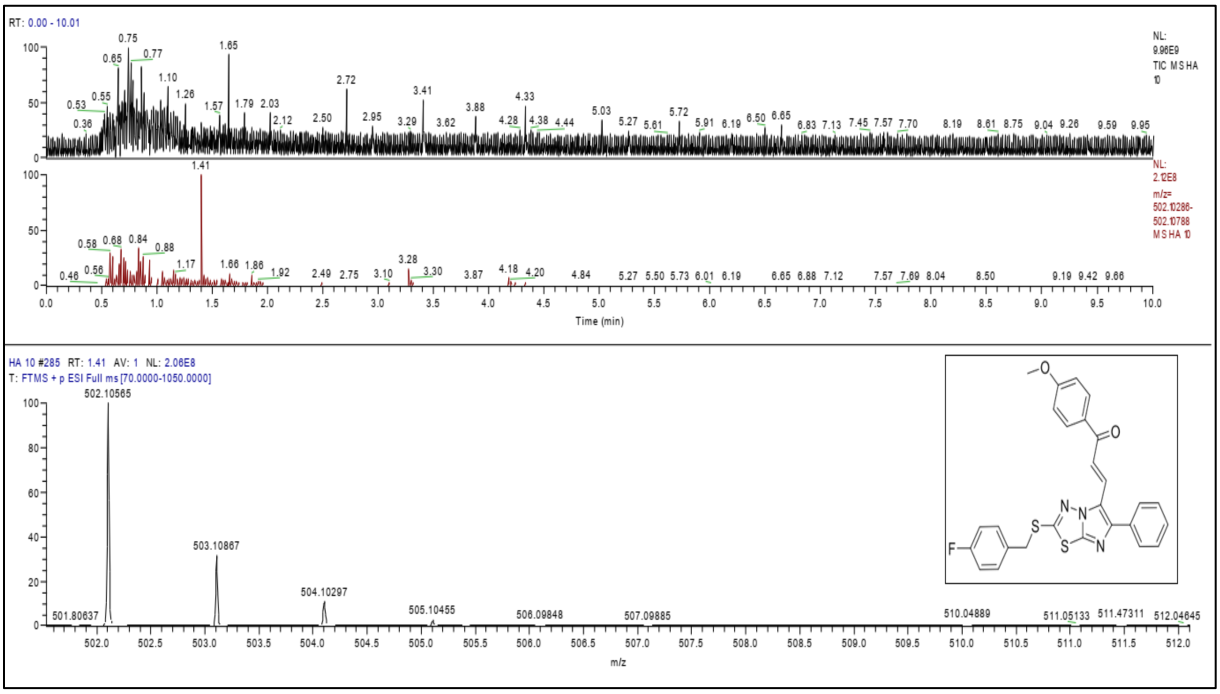

Figure S36. Mass Spectrum **(8c).**

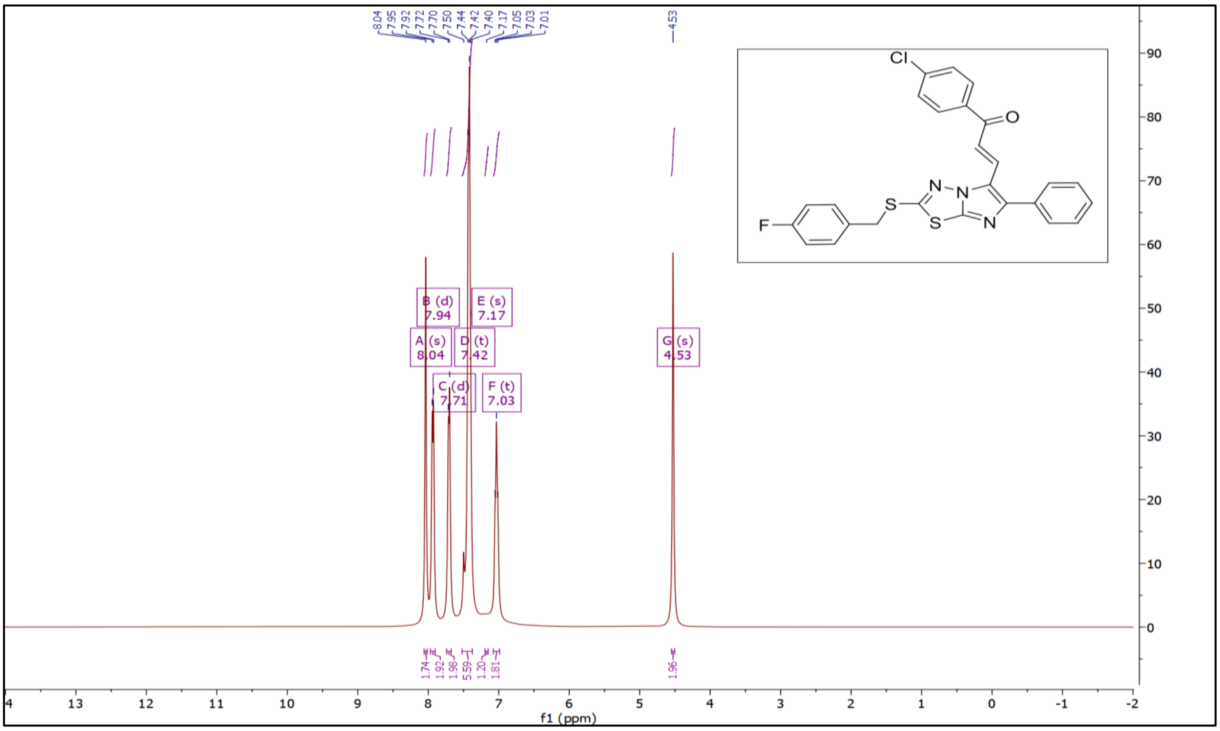

Figure S37. ^{1}H NMR Spectrum ($CDCl_3$) **(8d).**

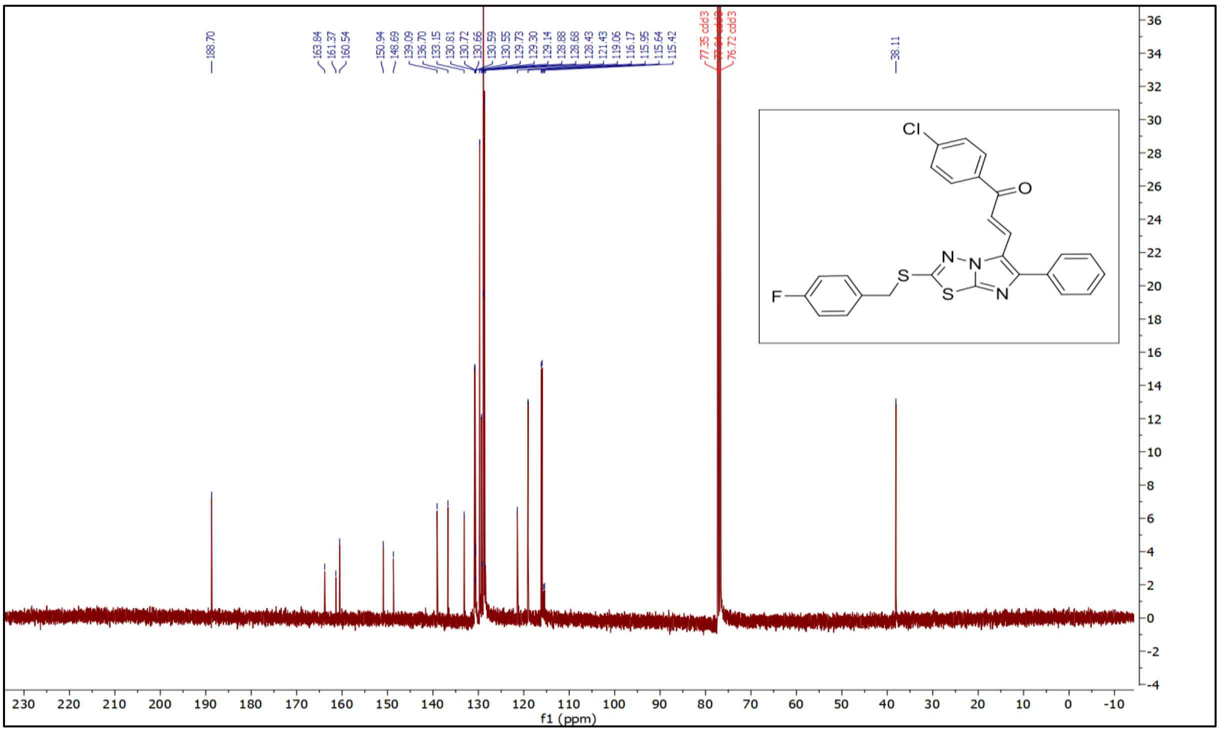

Figure S38. ^{13}C NMR Spectrum ($CDCl_3$) **(8d).**

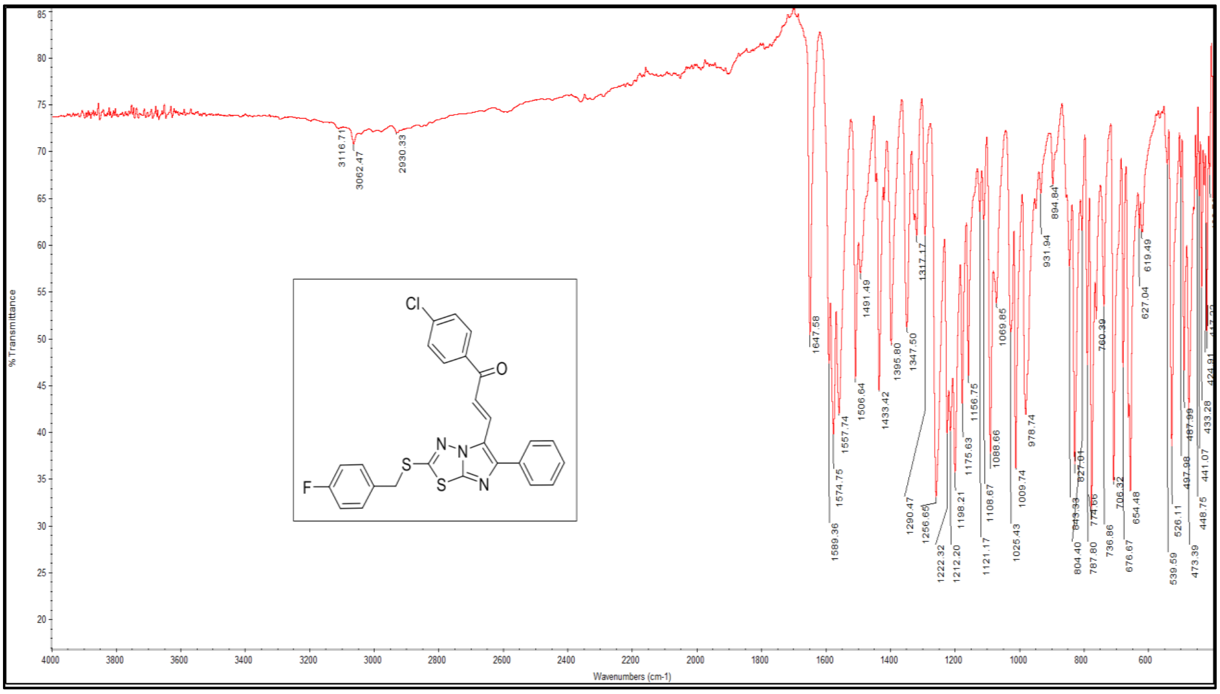

Figure S39. FT-IR Spectrum **(8d).**

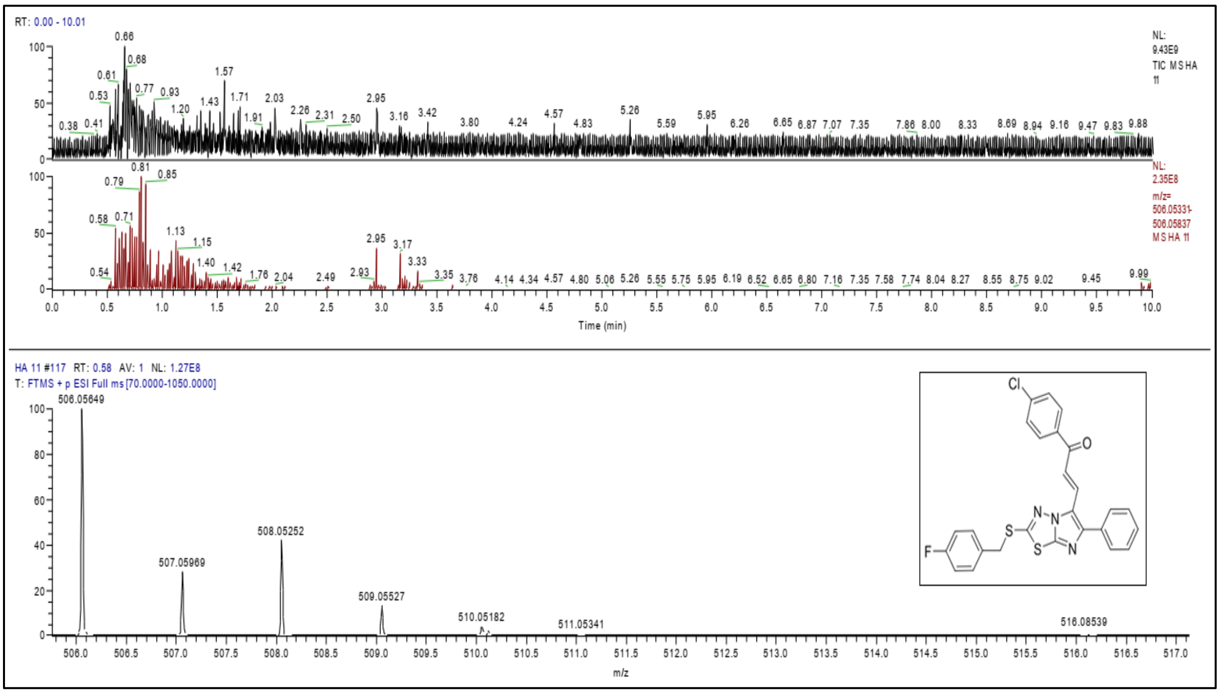

Figure S40. Mass Spectrum **(8d).**

```
CLUSTAL O(1.2.4) multiple sequence alignment


sp|P22303|ACES_HUMAN      MRPPQCLLHTPSLASPLLLLLLWLLGGGVGAEGREDAELLVTVRGGRLRGIRLKTPG-GP 59
sp|O42275|ACES_ELEEL      ------MKILDALLFPVIFIMFFI-H---LSIAQTDPELTIMTRLGQVQGTRLPVPDRSH 50
                                :    :*  *::::::::      : .: * ** : .* *:::* ** .*. .

sp|P22303|ACES_HUMAN      VSAFLGIPFAEPPMGPRRFLPPEPKQPWSGVVDATTFQSVCYQYVDTLYPGFEGTEMWNP 119
sp|O42275|ACES_ELEEL      VIAFLGIPFAEPPLGKMRFKPPEPKKPWNDVFDARDYPSACYQYVDTSYPGFSGTEMWNP 110
                          * ***********:*  ** *****:**..*.**  : *.******* ****.*******

sp|P22303|ACES_HUMAN      NRELSEDCLYLNVWTPYPRPTSPTPVLVWIYGGGFYSGASSLDVYDGRFLVQAERTVLVS 179
sp|O42275|ACES_ELEEL      NRMMSEDCLYLNVWVPATPRPHNLTVMVWIYGGGFYSGSSSLDVYDGRYLAHSEKVVVVS 170
                          ** :**********.*         *:***********:*********:*.::*:.*:**

sp|P22303|ACES_HUMAN      MNYRVGAFGFLALPGSREAPGNVGLLDQRLALQWVQENVAAFGGDPTSVTLFGESAGAAS 239
sp|O42275|ACES_ELEEL      MNYRVSAFGFLALNGSAEAPGNVGLLDQRLALQWVQDNIHFFGGNPKQVTIFGESAGAAS 230
                          *****.******* ** *******************:*:  ***:*..**:*********

sp|P22303|ACES_HUMAN      VGMHLLSPPSRGLFHRAVLQSGAPNGPWATVGMGEARRRATQLAHLVGCPPGGTGGNDTE 299
sp|O42275|ACES_ELEEL      VGMHLLSPDSRPKFTRAILQSGVPNGPWRTVSFDEARRRAIKLGRLVGCPD----GNDTD 286
                          ******** **  * **:****.***** **.:.****** :*.:*****     ****:

sp|P22303|ACES_HUMAN      LVACLRTRPAQVLVNHEWHVLPQESVFRFSFVPVVDGDFLSDTPEALINAGDFHGLQVLV 359
sp|O42275|ACES_ELEEL      LIDCLRSKQPQDLIDQEWLVLPFSGLFRFSFVPVIDGVVFPDTPEAMLNSGNFKDTQILL 346
                          *: ***::  * *:::** *** ..:********:** .: *****::*:*:*:. *:*:

sp|P22303|ACES_HUMAN      GVVKDEGSYFLVYGAPGFSKDNESLISRAEFLAGVRVGVPQVSDLAAEAVVLHYTDWLHP 419
sp|O42275|ACES_ELEEL      GVNQNEGSYFLIYGAPGFSKDNESLITREDFLQGVKMSVPHANEIGLEAVILQYTDWMDE 406
                          ** ::******:**************:* :** **::.**:..::. ***:*:****:.

sp|P22303|ACES_HUMAN      EDPARLREALSDVVGDHNVVCPVAQLAGRLA----------------------------- 450
sp|O42275|ACES_ELEEL      DNPIKNREAMDDIVGDHNVVCPLQHFAKMYAQYSILQGQTGTASQGNLGWGNSGSASNSG 466
                          ::* : ***:.*:*********: ::*   *

sp|P22303|ACES_HUMAN      AQGARVYAYVFEHRASTLSWPLWMGVPHGYEIEFIFGIPLDPSRNYTAEEKIFAQRLMRY 510
sp|O42275|ACES_ELEEL      NSQVSVYLYMFDHRASNLVWPEWMGVIHGYEIEFVFGLPLEKRLNYTLEEEKLSRRMMKY 526
                           . . ** *:*:****.* ** **** *******:**:**:   *** **: :::*:*:*

sp|P22303|ACES_HUMAN      WANFARTGDPNEPRDP---KAPQWPPYTAGAQQYVSLDLRPLEVRRGLRAQACAFWNRFL 567
sp|O42275|ACES_ELEEL      WANFARTGNPNINVDGSIDSRRRWPVFTSTEQKHVGLNTDSLKVHKGLKSQFCALWNRFL 586
                          ********:**   *    .  :** :*:  *::*.*:   *:*::**::* **:*****

sp|P22303|ACES_HUMAN      PKLLSATDTLDEAERQWKAEFHRWSSYMVHWKNQFDHYSKQDRCSDL 614
sp|O42275|ACES_ELEEL      PRLLNVTENIDDAERQWKAEFHRWSSYMMHWKNQFDHYSKQERCTNL 633
                          *:**..*:.:*:****************:************:**::*
```

Figure S41. Pairwise sequence alignment of human acetylcholinesterase (hAChE, UniProt ID: P22303) and Electrophorus electricus acetylcholinesterase (eAChE, UniProt ID: O42275).

```
CLUSTAL O(1.2.4) multiple sequence alignment


sp|P06276|CHLE_HUMAN      MHSKVTIICIRFLFWFLLLCMLIGKSHTEDDIIIATKNGKVRGMNLTVFGGTVTAFLGIP 60
sp|P81908|CHLE_HORSE      ----------------------------EEDIIITTKNGKVRGMNLPVLGGTVTAFLGIP 32
                                                      *:****:*********** *:***********

sp|P06276|CHLE_HUMAN      YAQPPLGRLRFKKPQSLTKWSDIWNATKYANSCCQNIDQSFPGFHGSEMWNPNTDLSEDC 120
sp|P81908|CHLE_HORSE      YAQPPLGRLRFKKPQSLTKWSNIWNATKYANSCYQNTDQSFPGFLGSEMWNPNTELSEDC 92
                          *********************:*********** ** ******* *********:*****

sp|P06276|CHLE_HUMAN      LYLNVWIPAPKPKNATVLIWIYGGGFQTGTSSLHVYDGKFLARVERVIVVSMNYRVGALG 180
sp|P81908|CHLE_HORSE      LYLNVWIPAPKPKNATVMIWIYGGGFQTGTSSLPVYDGKFLARVERVIVVSMNYRVGALG 152
                          *****************:*************** **************************

sp|P06276|CHLE_HUMAN      FLALPGNPEAPGNMGLFDQQLALQWVQKNIAAFGGNPKSVTLFGESAGAASVSLHLLSPG 240
sp|P81908|CHLE_HORSE      FLALSENPEAPGNMGLFDQQLALQWVQKNIAAFGGNPRSVTLFGESAGAASVSLHLLSPR 212
                          ****  *******************************:*********************

sp|P06276|CHLE_HUMAN      SHSLFTRAILQSGSFNAPWAVTSLYEARNRTLNLAKLTGCSRENETEIIKCLRNKDPQEI 300
sp|P81908|CHLE_HORSE      SQPLFTRAILQSGSSNAPWAVTSLYEARNRTLTLAKRMGCSRDNETEMIKCLRDKDPQEI 272
                          *: *********** *****************.***  ****:****:*****:******

sp|P06276|CHLE_HUMAN      LLNEAFVVPYGTPLSVNFGPTVDGDFLTDMPDILLELGQFKKTQILVGVNKDEGTAFLVY 360
sp|P81908|CHLE_HORSE      LLNEVFVVPYDTLLSVNFGPTVDGDFLTDMPDTLLQLGQFKRTQILVGVNKDEGTAFLVY 332
                          ****.*****.* ******************* **:*****:******************

sp|P06276|CHLE_HUMAN      GAPGFSKDNNSIITRKEFQEGLKIFFPGVSEFGKESILFHYTDWVDDQRPENYREALGDV 420
sp|P81908|CHLE_HORSE      GAPGFSKDNNSIITRKEFQEGLKIFFPRVSEFGRESILFHYMDWLDDQRAENYREALDDV 392
                          *************************** *****:******* **:**** *******.**

sp|P06276|CHLE_HUMAN      VGDYNFICPALEFTKKFSEWGNNAFFYYFEHRSSKLPWPEWMGVMHGYEIEFVFGLPLER 480
sp|P81908|CHLE_HORSE      VGDYNIICPALEFTRKFSELGNDAFFYYFEHRSTKLPWPEWMGVMHGYEIEFVFGLPLER 452
                          *****:********:**** **:**********:**************************

sp|P06276|CHLE_HUMAN      RDNYTKAEEILSRSIVKRWANFAKYGNPNETQNNSTSWPVFKSTEQKYLTLNTESTRIMT 540
sp|P81908|CHLE_HORSE      RVNYTRAEEILSRSIMKRWANFAKYGNPNGTQNNSTRWPVFKSTEQKYLTLNTESPKVYT 512
                          * ***:*********:************* ****** ****************** :: *

sp|P06276|CHLE_HUMAN      KLRAQQCRFWTSFFPKVLEMTGNIDEAEWEWKAGFHRWNNYMMDWKNQFNDYTSKKESCV 600
sp|P81908|CHLE_HORSE      KLRAQQCRFWTLFFPKVLELTGNIDEAEREWKAGFHRWNNYMMDWKNQFNDYTSKKESCS 572
                          *********** *******:******** ******************************

sp|P06276|CHLE_HUMAN      GL 602
sp|P81908|CHLE_HORSE      DF 574
                          .:
```

Figure S42. Pairwise sequence alignment of human butyrylcholinesterase (hBChE, UniProt ID: P06276) and equine butyrylcholinesterase (eBChE, UniProt ID: P81908)

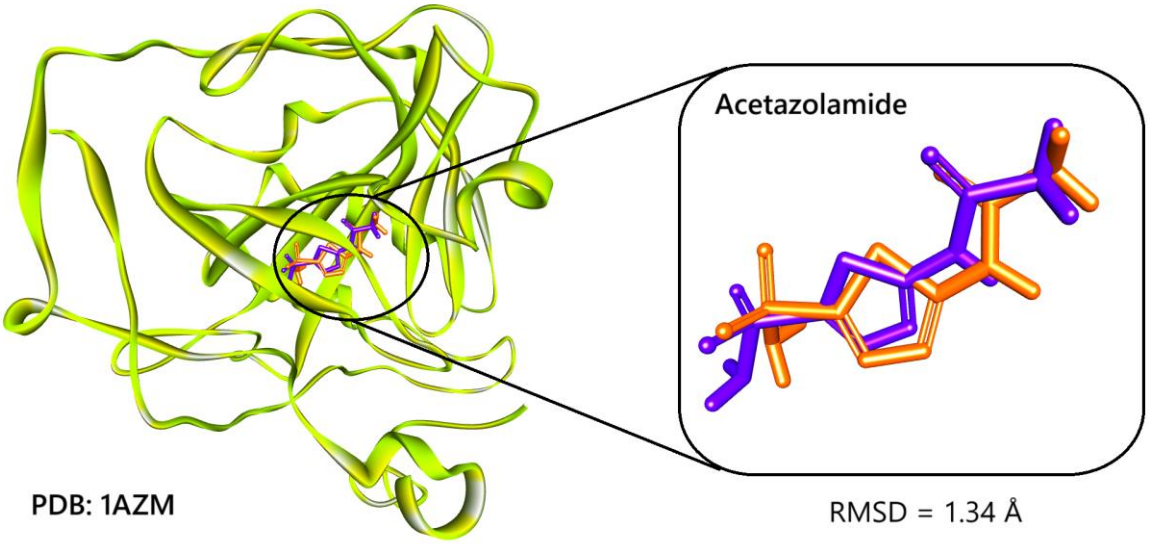

Figure S43. Redocking validation of acetazolamide in the active site of hCA I (PDB: 1AZM). Superposition of the crystallographic pose (orange) and the redocked pose (purple) shows excellent overlap, with an RMSD of 1.34 Å, confirming the accuracy of the docking protocol for hCA I.

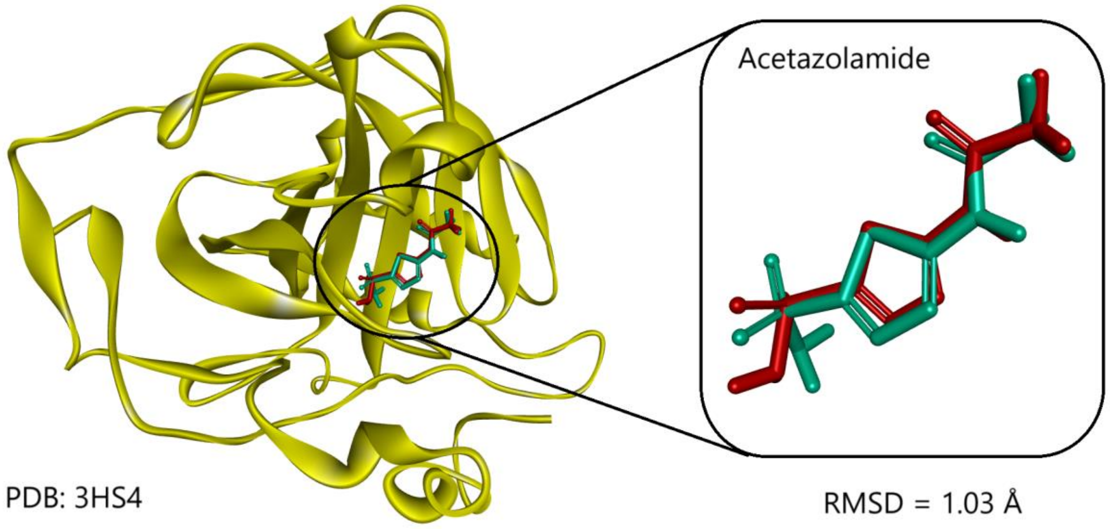

Figure S44. Redocking validation of acetazolamide in the active site of hCA II (PDB: 3HS4). The redocked pose (cyan) closely matches the crystallographic pose (red), yielding an RMSD of 1.03 Å. This supports the reliability of the docking method used for hCA II.

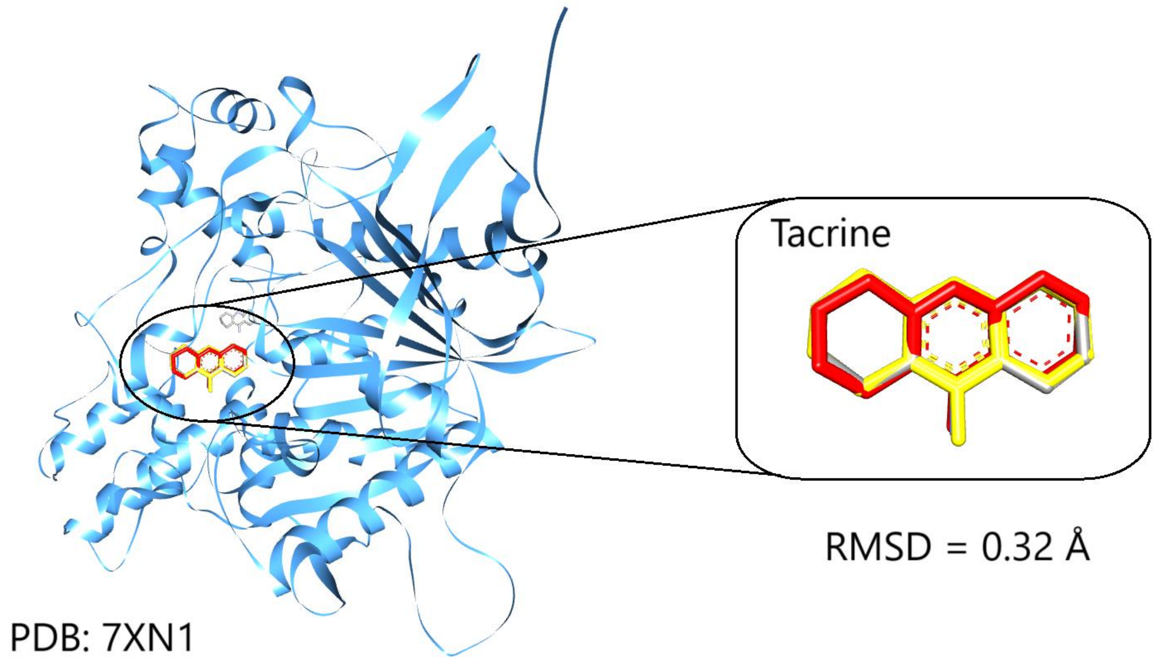

Figure S45. Redocking validation of tacrine in the active site of AChE (PDB: 7XN1). The superposition of the co-crystallized pose (red) and the redocked conformation (yellow) shows an RMSD of 0.32 Å, indicating high consistency between predicted and experimental binding.

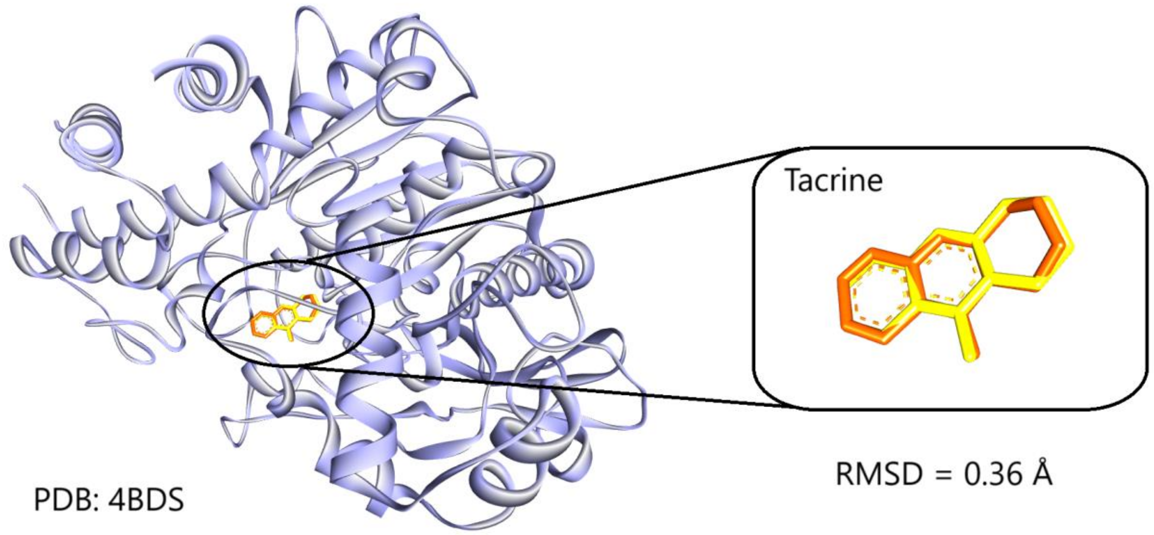

Figure S46. Redocking validation of tacrine in the active site of BChE (PDB: 4BDS). The comparison of the crystallographic (orange) and redocked (yellow) poses reveals an RMSD of 0.36 Å, confirming the suitability of the docking parameters for BChE.
